# Prospects of compounds of herbal plants as anticancer agents: a comprehensive review from molecular pathways

**DOI:** 10.3389/fphar.2024.1387866

**Published:** 2024-07-22

**Authors:** Putri Cahaya Situmorang, Syafruddin Ilyas, Sony Eka Nugraha, Rony Abdi Syahputra, Nik Mohd Afizan Nik Abd Rahman

**Affiliations:** ^1^ Study Program of Biology, Faculty of Mathematics and Natural Sciences, Universitas Sumatera Utara, Medan, Indonesia; ^2^ Department of Pharmaceutical Biology, Faculty of Pharmacy, Universitas Sumatera Utara, Medan, Indonesia; ^3^ Department of Pharmacology, Faculty of Pharmacy, Universitas Sumatera Utara, Medan, Indonesia; ^4^ Department of Cell and Molecular Biology, Faculty of Biotechnology and Biomolecular Sciences, Universiti Putra Malaysia, Serdang, Malaysia

**Keywords:** apoptosis, cancer, herbal, molecular pathway, phytochemicals, plants

## Abstract

Cancer refers to the proliferation and multiplication of aberrant cells inside the human body, characterized by their capacity to proliferate and infiltrate various anatomical regions. Numerous biochemical pathways and signaling molecules have an impact on the cancer auto biogenesis process. The regulation of crucial cellular processes necessary for cell survival and proliferation, which are triggered by phytochemicals, is significantly influenced by signaling pathways. These pathways or components are regulated by phytochemicals. Medicinal plants are a significant reservoir of diverse anticancer medications employed in chemotherapy. The anticancer effects of phytochemicals are mediated by several methods, including induction of apoptosis, cessation of the cell cycle, inhibition of kinases, and prevention of carcinogenic substances. This paper analyzes the phytochemistry of seven prominent plant constituents, namely, alkaloids, tannins, flavonoids, phenols, steroids, terpenoids, and saponins, focusing on the involvement of the MAPK/ERK pathway, TNF signaling, death receptors, p53, p38, and actin dynamics. Hence, this review has examined a range of phytochemicals, encompassing their structural characteristics and potential anticancer mechanisms. It has underscored the significance of plant-derived bioactive compounds in the prevention of cancer, utilizing diverse molecular pathways. In addition, this endeavor also seeks to incentivize scientists to carry out clinical trials on anticancer medications derived from plants.

## 1 Introduction

Cancer is a pathological condition defined by the excessive and uncontrolled growth of abnormal cells within the human body. These abnormal cells exhibit the ability to proliferate and invade any region of the organism (Brown et al., 2023). Cancer is considered the second leading cause of mortality globally, following stroke and heart disease (Sun and Bhaskar, 2022). Cancer is classified based on the cellular genesis of the tumor. Carcinoma, a cancer that arises from the epithelial cells of the breast, prostate, lung, pancreas, and colon, is responsible for 90% of all cancer-related deaths in humans (Łukasiewicz et al., 2021). Lymphoma, on the other hand, is a cancer that affects immune organs like the spleen, white blood cells, and lymph nodes (Weledji and Orock, 2015). Leukemia is a cancer that affects the blood cells that make up the bone marrow (Bispo et al., 2020). Sarcoma is a cancer that affects the fibrous connective tissue of bones, cartilage, fatty tissue, muscles, and neurons (Vodanovich and M Choong, 2018). Lastly, germ cell tumors originate from pluripotent stem cells found in the testes and ovaries (Hong et al., 2021). Cell metabolism is intricately interconnected within a multifaceted biological network that encompasses various metabolites and ubiquitous mechanisms for sensing these compounds. Signal transduction and epigenetics of these pathways can be regulated by either endogenous or exogenous metabolites. Nevertheless, the occurrence of irregular metabolic reprogramming in cancer might result in atypical suppression and stimulation of metabolite sensing, hence playing a substantial role in the advancement of cancer (Schiliro and Firestein, 2021). Timely identification and efficient therapy enhance the likelihood of survival in cancer patients. Hence, it is imperative to develop a comprehensive strategy aimed at enhancing cancer prevention and treatment. Epidemiological and experimental studies have provided evidence supporting the notion that a substantial consumption of fruits, vegetables, or medicinal plants can effectively mitigate the prevalence of chronic degenerative diseases (Cruz et al., 2024). Moreover, the significance of maintaining a well-balanced diet in the context of cancer prevention has garnered considerable scholarly interest.

Medicinal plants have served as a valuable reservoir of diverse anticancer medications that are being employed in chemotherapy. Bioactive compounds, known as phytochemicals, play a crucial role in anticancer treatment (Yuan et al., 2022). These sources are secure, harmless, economical, and easily accessible, spanning from rural to urban areas and underdeveloped to developed nations (Irianti et al., 2020). Hence, there is growing interest in exploring the possible anticancer properties of herbal substances. Chemoprevention refers to the application of both natural and synthetic chemicals for impeding or decelerating the progression of cancer through the inhibition or disruption of specific molecular signaling pathways (G et al., 2021). The investigation of the impact of phytochemicals on cellular signaling is presently a subject of contemporary scholarly research. These substances have distinct modes of action against tumors. Bioactive compounds (phytochemicals) play a crucial role in the field of anticancer treatment (Situmorang et al., 2022a). The gynogenesis movement is impacted by multiple biochemical pathways and signaling molecules. Signaling pathways and molecular networks play a crucial role in the regulation of vital cellular processes that are required for the survival and proliferation of cells. The etiology of cancer necessitates a correlation with distinct biological pathways (Fawaz et al., 2023). Researchers persist in employing this methodology to further the progress of molecular therapy. Various molecular biology methodologies have been developed to detect and treat cancer, including the targeting of cancer stem cell pathways for treatment, utilization of retroviral therapy, suppression of oncogenes, and the alteration of tumor suppressor genes (Simanullang RH. et al., 2022). The regulation of cell proliferation, differentiation, survival, apoptosis, invasion, migration, angiogenesis, and metastatic spread of cancer cells is connected with various signaling pathways, including mTOR, PI3K, protein kinase B (Akt), MAPK/ERK, Wnt, Notch, and Hedgehog (Kciuk et al., 2022). The anticancer activities are induced by phytochemicals through the regulation of certain pathways or components.

Multiple independently conducted investigations have indicated that phytochemicals induce anticancer effects through various pathways (Choudhari et al., 2019). Nevertheless, there is a lack of detailed reporting regarding the phytochemical composition of seven plant components, namely, alkaloids, tannins, flavonoids, phenols, steroids, terpenoids, and saponins, in relation to their potential as anticancer agents (Simanullang et al., 2022b). There are no reports regarding the regulation of cell proliferation and inhibition of angiogenesis and metastasis through the MAPK/ERK pathway, TNF signaling, death receptors, p53, p38, and actin dynamics. Thus, this review has examined different phytochemicals, including their structures and probable anticancer mechanisms. As a result, it offers comprehensive knowledge on the potential of natural anticancer resources.

## 2 Role of phytochemicals and antioxidants in inhibiting the resistance of cancer to therapy

Utilizing phytochemicals for cancer chemoprevention is the preferred method for managing cancer. Investigating novel plant-derived chemicals with anticancer characteristics is a crucial objective in pharmacological research as it enables the discovery of new therapeutic targets (Efferth et al., 2017). This review highlights specific chemicals that exhibit potential as effective chemo-preventive agents for cancer. It is worth noting that the consumption of foods containing these bioactive compounds has demonstrated both protective and therapeutic benefits against different forms of cancer (Bakrim et al., 2022). The efficacy of chemotherapy and radiotherapy is enhanced by chemo-preventive medicines through regulation of various signal transduction pathways. Given the significant involvement of oxidative stress in the development of numerous malignancies, the potential of substances with antioxidant properties as a preventive measure against cancer is worth considering (Qi et al., 2022). Plants are rich in antioxidant phytochemicals, which encompass a diverse range of molecules (Simanullang et al., 2022c). These substances have distinct modes of action against tumors. Bioactive compounds (phytochemicals) play a crucial role in the field of anticancer treatment. Medicinal plants or their derivatives currently constitute over 70% of anticancer chemicals, making them the primary focus in the development of anticancer medications (Talib et al., 2020). The anticancer properties of plants are attributed to seven primary bioactive compounds: alkaloids, tannins, flavonoids, phenols, steroids, terpenoids, and saponins. Alkaloids are significant chemical substances that constitute a plentiful resource for the exploration of new drugs. Several alkaloids derived from medicinal plants and herbs have demonstrated antiproliferative and anticancer properties against a diverse range of malignancies, both in laboratory settings (*in vitro*) and in living organisms (*in vivo*) (Lu et al., 2012). Vinblastine, vinorelbine, vincristine, and vindesine have been effectively formulated as pharmaceutical agents for the treatment of cancer. Tannins demonstrate a wide range of therapeutic advantages, including their ability to combat cancer, act as an antioxidant, reduce inflammation, and protect the nervous system (Dhyani et al., 2022). Flavonoids are widely recognized for their efficacy as antioxidants and their ability to inhibit angiogenesis. Numerous studies have documented the inhibitory effects of flavonoids on the metabolic activation of carcinogens, hence impeding the proliferation of aberrant cells that have the potential to differentiate into malignant cells (Wang et al., 2022). Phenols in foods have multiple functions, including acting as antioxidants to eliminate cancer-causing free radicals, activating cytoprotective enzymes involved in detoxifying foreign substances, and regulating signal transduction systems (Aghajanpour et al., 2017). The involvement of antioxidants in the activation of the Keap1/Nrf2/ARE pathway leads to heightened levels of phase 2 detoxification enzymes and antioxidant enzymes (Mendonca and Soliman, 2020). The anticancer effects of terpenes may be attributed to their capacity to regulate several signaling pathways associated with cellular proliferation, death, and angiogenesis. The anticancer effects of terpenes may also be attributed to their ability to induce oxidative stress and DNA damage in cancer cells (Wróblewska-Łuczka et al., 2023). Steroids encompass a class of naturally occurring organic compounds that have steroidal anticancer properties. This class of chemicals exhibits a wide range of structural molecular diversity and possesses the capacity to interact with diverse biological targets and pathways. Saponins exhibit several anticancer properties, such as inhibiting cell growth, impeding metastasis, inhibiting angiogenesis, and reversing multidrug resistance (MDR) (Elekofehinti et al., 2021). These effects are caused by the initiation of apoptosis, stimulation of cell differentiation, modulation of the immune system, binding of bile acids, and improvement of cell proliferation caused by carcinogens.

Oxidative stress plays a crucial role in the harmful effects of environmental toxicity in cancer development, and reactive oxygen species (ROS) are produced in response to both internal and external triggers (Chang et al., 2015). Reactive oxygen species (ROS) such as superoxide radicals (O_2_−.), hydrogen peroxide (H_2_O_2_), singlet oxygen (1O_2_), and hydroxyl radicals (HO.) are harmful to cells and have been linked to the development of different human diseases, including cancer (Afzal et al., 2023). Several carcinogens exert their effects by generating reactive oxygen species (ROS) during their metabolic process (Afzal et al., 2023). Oxidative DNA damage is a significant factor in the development and advancement of carcinogenesis as it can cause mutations (Martemucci et al., 2022). Hence, the crucial role of antioxidants in counteracting elevated levels of reactive oxygen species (ROS) is significant in the context of numerous disorders, including different forms of cancer (Afzal et al., 2023). Epidemiological studies form the primary basis for establishing the correlation between dietary antioxidants and non-communicable diseases, such as cancer. These studies indicate that plant foods and phytochemicals have the capacity to prevent cancer. Certain phytochemicals have the ability to function as both antioxidants and prooxidants (Situmorang and Ilyas, 2018). They can generate reactive oxygen species (ROS) and induce oxidative stress at high levels, particularly when iron and copper are present. Polyphenols, including quercetin, epicatechin, epigallocatechin-3-gallate (EGCG), and gallic acid, have been found to generate reactive oxygen species (ROS) in cell models due to their prooxidant properties (Yang et al., 2020). Phytochemicals are widely recognized for their antioxidant properties, but they can also display prooxidant activity under specific circumstances, such as when administered in excessive amounts or in the presence of metal ions (Yang et al., 2020). The concentration of phytochemicals plays a crucial role in determining whether they exhibit prooxidant or antioxidant activity (Situmorang et al., 2021a). Studies using cell models have highlighted the prooxidant activity of polyphenols, which are known for their antioxidant properties. Specifically, compounds such as quercetin, epicatechin, and epigallocatechin-3-gallate (EGCG) have been found to demonstrate this prooxidant activity. At elevated concentrations, such as 50 μM, quercetin enhances the generation of superoxide radicals (O_2_−) in isolated mitochondria and cell culture medium (Yang et al., 2020; Safi et al., 2021). Previous research has demonstrated that quercetin can decrease cell viability and thiol content, as well as impair overall antioxidant capacity and the activities of SOD, CAT, and glutathione transferase at higher concentrations (Safi et al., 2021). High amounts of flavonoids can generate reactive oxygen species (ROS) through processes such as autoxidation and redox cycles, as seen in quercetin (Safi et al., 2021).

Dietary phytochemicals can activate many cell signaling pathways, and the specific route triggered by a molecule can vary depending on the type of cell (Hun Lee et al., 2013). Elevated levels of pro-apoptotic p53 and reduced levels of key pro-survival factors, such as epidermal growth factor receptor (EGFR), nuclear factor-kappa B (NF-κB), activator protein 1, signal transducers and activators of transcription (STAT), survivin, metalloproteinases 2 and 9, vascular endothelial growth factor (VEGF), and B-cell leukemia/lymphoma 2 (Bcl-2), are observed under optimal conditions when phytochemicals are administered (Gupta et al., 2012). The effectiveness of phytochemicals in cancer treatment stems from their capacity to influence many signaling pathways concurrently, hence facilitating apoptosis, impeding cellular proliferation and invasion, sensitizing malignant cells, and enhancing immune system functionality (George et al., 2021). The synergistic effect of cytotoxic anticancer drugs and phytochemical inhibitors can synergistically reduce tumor growth (Cheon and Ko, 2022). The phytochemical composition of the seven primary plant constituents, namely, flavonoids, alkaloids, terpenoids, steroids, saponins, phenol, and tannins, as anticancer agents is shown in Supplementary Table S1.

## 3 Role of phytochemicals in the MAPK/ERK pathway

**FIGURE 1:**
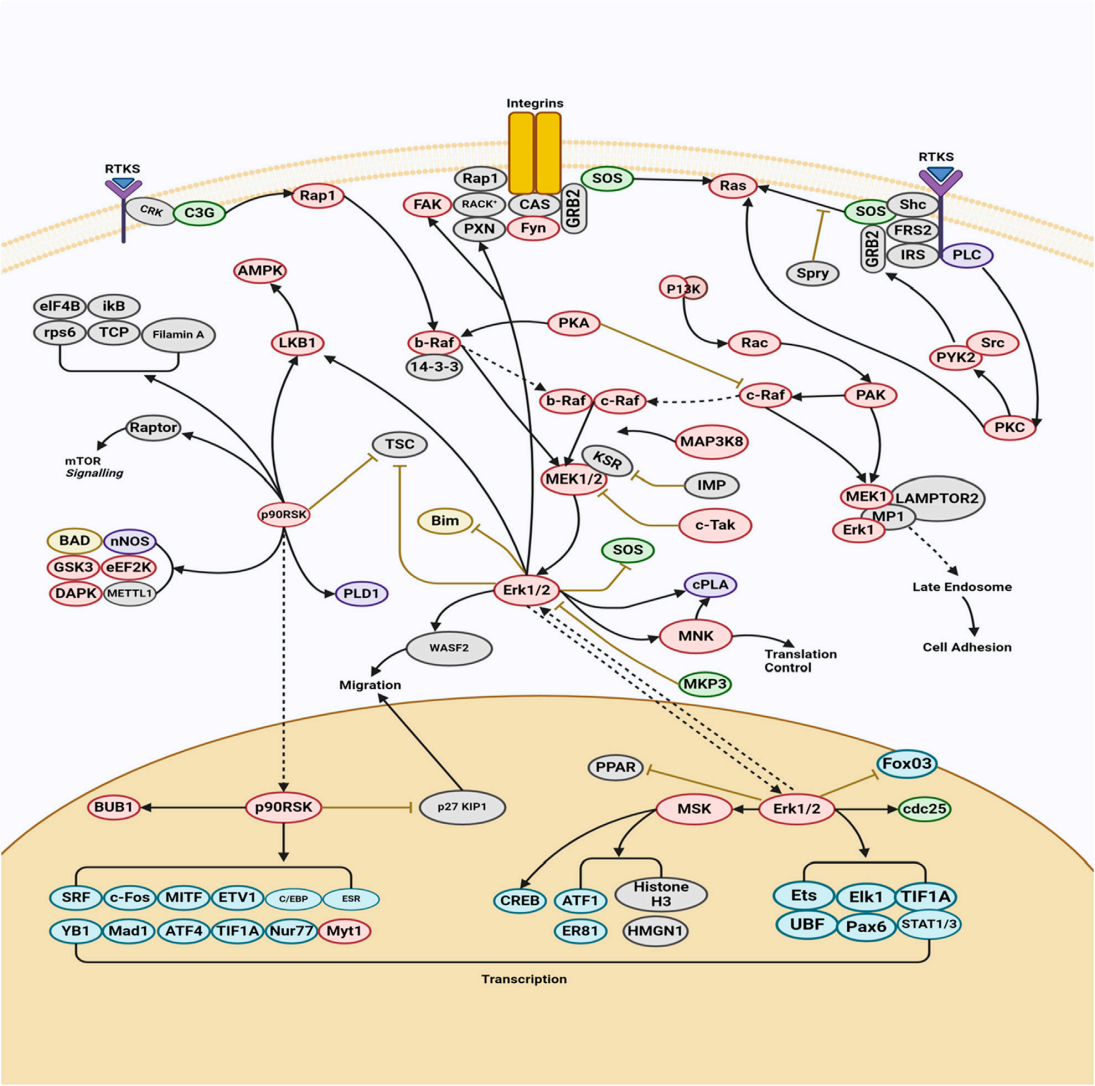
MAPK/ERK pathway in cancer. The activation of MAPK initiates with the stimulation of receptor tyrosine kinase (RTK) in the MAPK/ERK signaling pathway. This leads to the activation of RAF kinase protein kinase activity through Ras activation. Subsequently, MEK (MEK1 and MEK2) is phosphorylated and activated by RAF kinase. Finally, ERK is activated and phosphorylated by MEK (https://www.sinobiological.com/pathways).

The MAPK/ERK pathway is a cellular protein chain responsible for transmitting signals from cell surface receptors to DNA within the cell nucleus. The development of certain human disorders, including as Alzheimer’s disease (AD), Parkinson’s disease (PD), amyotrophic lateral sclerosis (ALS), and several types of cancer, has been associated with deviations from strict regulation of the MAPK signaling pathway (Albert-Gascó et al., 2020). This particular route encompasses a multitude of proteins, including mitogen-activated protein kinase (MAPK, formerly known as ERK). These proteins engage in communication by introducing phosphate groups to adjacent proteins, thus functioning as active or inactive switches. MAPKs represent a group of serine/threonine protein kinases that exhibit a high degree of conservation. These kinases play a crucial role in numerous essential cellular processes, including but not limited to proliferation, differentiation, motility, stress response, apoptosis, and survival (Chen et al., 2021). There have been characterizations of at least three MAPK families, namely, extracellular signal-regulated kinase (ERK), Jun kinase (JNK/SAPK), and p38 MAPK. The aforementioned effects are achieved through the modulation of cell-cycle machinery and other proteins associated with cell proliferation (Min and Lee, 2023). Figure 1 investigates the function of this system in conjunction with other signaling pathways to regulate the proliferation of cancer cells. MAPK activation is initiated in a multistep process through the stimulation of receptor tyrosine kinase (RTK) in the MAPK/ERK signaling pathway. The protein kinase activity of RAF kinase is facilitated by the activation of Ras. The phosphorylation and activation of MEK (MEK1 and MEK2) is facilitated by RAF kinase. ERK is activated and phosphorylated by MEK (Santarpia et al., 2012). The role of the MAPK/ERK pathway in the translation of extracellular signals to cellular responses has been demonstrated to be significant. The protein kinase cascade encompasses the presence of MAP kinase (Cargnello and Roux, 2011). The cascade comprises a minimum of three enzymes that are sequentially activated: MAPK kinase kinase (MAPKKK), MAPK kinase (MAPKK), and MAP kinase (MAPK). The MAPK/ERK pathway is involved in multiple processes associated with cancer, such as proliferation, invasion, metastasis, angiogenesis, and apoptosis inhibition. The MAPK/ERK pathway has a significant impact on encouraging cancer cell proliferation and preventing apoptosis due to its diverse actions (Kciuk et al., 2022). For instance, the compound β-carboline has been observed to impede cell growth and trigger apoptosis in SGC-7901 cells. This disruption of the PTEN and ERK balance leads to the inhibition of the MAPK/ERK signaling pathway, ultimately resulting in apoptosis in SGC-7901 cells (Qin et al., 2022). Berberine has the ability to block the EGFR/Raf/MEK/ERK pathway, hence suppressing the aging process in human glioblastoma cells. Sinomenine suppresses the growth of many cancer cells (Liu et al., 2015). The phosphorylation of ERK1/2 and p38 is enhanced by the presence of sinomenine hydrochloride (SH). The phosphorylation of ERK1/2 was considerably increased by the benzo alkaloid chelerythrine chloride, while the phosphorylation of Akt was lowered in a dose-dependent manner (Peng et al., 2023). Table 1 presents the mechanism of action of various phytochemical compounds in the MAPK/ERK pathway in cancer.

**TABLE 1:** Mechanism of action of various phytochemical compounds in the MAPK/ERK pathway in cancer.

Target	Compounds	Types of study	Mechanism of action	Cell line(s)/animal model(s)	References
ERK1/2 MAPK	Astaxanthin	*In vivo*	Astaxanthin reduces NF-κB and Wnt signaling by reducing IKKβ and GSK-3β activity. Analysis of gene expression and docking interactions showed that astaxanthin may block these pathways by inactivating Erk/Akt	Male Syrian hamsters	Kavitha et al. (2013)
ERK1/2 MAPK	α-Mangostin	*In vitro*	α-Mangostin inhibits the activation of extracellular signal-regulated kinases 1 and 2 (ERK1/2), which are involved in the downregulation of enzyme activity, protein, and messenger RNA levels of MMP-2 and MMP-9	MCF-7 human breast adenocarcinoma cells	Hafeez et al. (2014)
ERK1/2 MAPK	Arctigenin	*In vitro*	Arctigenin decreased MMP-9 activity and COX-2 and MMP-3 protein expression and also reduced the mRNA expression of metastatic factors such as MMP-9, MMP-3, and COX-2 via the mitogen-activated protein MAPK/AP1 signaling pathway, which was examined to determine its anti-metastatic mechanism	4T-1 mouse breast cancer cells	Lee et al. (2020b)
ERK1/2 MAPK	Baicalein	*In vitro* and *in vivo*	Baicalein decreases the levels of phosphorylated MEK1 and ERK1/2, but MEK1 overexpression partially limits its anti-metastatic action, decreasing the expression of MMP-2, MMP-9, and u-PA and increasing the expression of TIMP-1 and TIMP-2	The human HCC cell line MHCC97H, an orthotopic transplanted nude mouse model of HCC metastasis	Chen et al. (2013b)
ERK1/2 MAPK	Curcumin	*In vitro*	Curcumin induces mitochondrial membrane depolarization via MAPK, which regulates anticancer effects by activating ERK1/2, SAPK/JNK, P90RSK, and c-Jun	JAR and JEG3 cells (human placental choriocarcinoma cells)	Lim et al. (2016)
ERK1/2 MAPK	Cinnamaldehyde	*In vitro*	Activated macrophages treated with cinnamaldehyde showed lower mRNA expression and secretion of IL-1β, IL-6, and TNF-α, leading to anti-inflammatory effects by decreasing ERK, JNK, and p38 MPAK phosphorylation	The RAW 264.7 murine macrophage cell line	Kim et al. (2018)
ERK1/2 MAPK	Damnacanthal	*In vitro*	Treatment with caspase inhibitors and soluble death receptors that activate p38 MAPK can decrease apoptosis induced by damnacanthal, which is mediated via TRAIL and TNF- α	SKHep 1 cells	Lin et al. (2011)
ERK1/2 MAPK	Diosgenin	*In vitro* and *in vivo*	Diosgenin suppressed the Raf/MEK/ERK pathway, a downstream target of Akt, in ER+ but not ER− BCa cells. Diosgenin inhibits cell proliferation and induces apoptosis in ER+ and ER− BCa cells by downregulating cyclin D1, cdk-2, and cdk-4 expression, causing G1 cell-cycle arrest	MCF-7 (ER+), MDA 231 (ER−), and MCF-10A; female nude mice as xenograft study models	Srinivasan et al. (2009)
ERK1/2 MAPK	(−)-Epigallocatechin-3-gallate	*In vitro*	Berberine may increase cisplastin sensitivity by reducing drug transporter expression (MDR1 and MRP1), increasing apoptosis, and suppressing PI3K/AKT/mTOR and ERK/MAPK signaling	BGC-823 and SGC-7901 cells	Wu et al. (2019)
ERK1/2 MAPK	Licochalcone A	*In vitro*	The expression of TRAIL was stimulated by licochalcone A through the activation of both the ERK1/2 and p38 MAPK signaling pathways	Normal human oral keratinocytes (hNOKs)	Park et al. (2015)
ERK1/2 MAPK	Paclitaxel (Taxol)	*In Vivo*	ERK1/2 is activated in spinal cord and dorsal root ganglion (DRG) neurons, glia, and active brain areas, and paclitaxel has been shown to increase DRG ERK1/2 activation	Adult C57/BL6J mice	Kim et al. (2023)
ERK1/2 MAPK	Quercetin	*In vitro*	Quercetin has little effect on the ERK/MAPK pathway; even ERK1/2 levels increased after docetaxel treatment. PKB, ERK1/2, and STAT3 are proliferation and signaling mediators and survival signals	MDA-MB-231 breast cancer cell line	Safi et al. (2021)
ERK1/2 MAPK	Resveratrol	*In vivo*	Resveratrol affects SOD, catalase, and GPx in hyperalgesia and rat paw skin and spinal cord. Resveratrol affected ERK signaling but not TNFR1	Charles Foster strain rats	Singh and Vinayak (2017)
ERK1/2 MAPK	Silibinin	*In vitro* and *in vivo*	Inhibition of the ERK protein by silibinin significantly decreased the mitochondrial membrane potential, releasing cytochrome C. Cholangiocarcinoma cells died once downstream apoptotic mechanisms were activated	BALB/c nude mice and HuCCT-1 and CCLP-1, two human cholangiocarcinoma cell lines	Bai et al. (2022)

## 4 Role of phytochemicals in the TNF-signaling pathway

**FIGURE 2:**
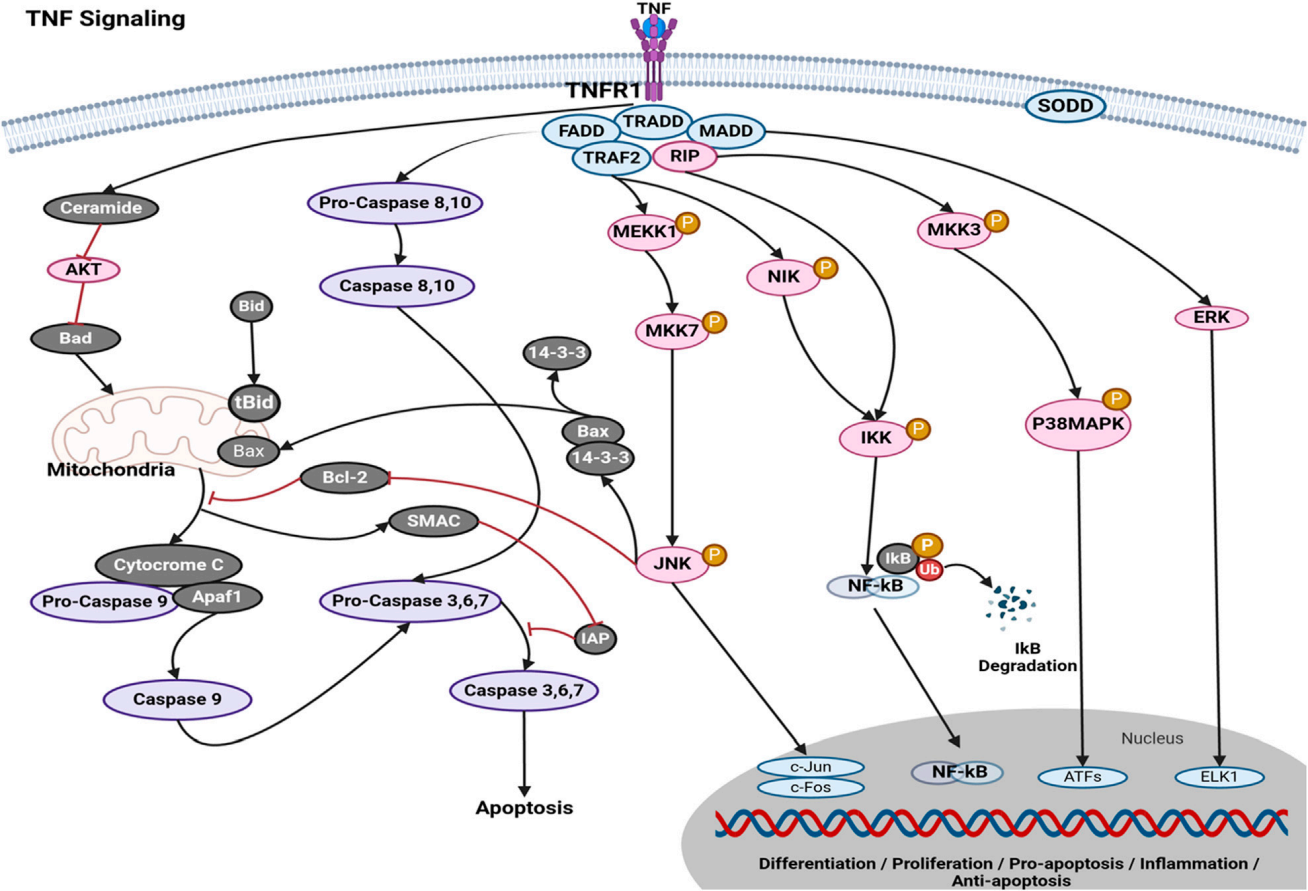
TNF-signaling pathways in cancer. Tumor necrosis factor (TNF) exerts its actions through the tumor necrosis factor receptor (TNFR), hence engaging in the extrinsic pathway to promote apoptosis. The interaction between the TNFR and procaspases is facilitated by adapter proteins, such as FADD and TRADD. These adapter proteins possess the capability to cleave inactive procaspases, hence activating the caspase cascade. This series of events ultimately results in the irreversible initiation of cell death (https://www.sinobiological.com/pathways).

The activation of signaling pathways for cell survival, death, and differentiation is facilitated by the tumor necrosis factor (TNF) superfamily of cytokines. TNF signaling has witnessed a growing trend in the therapeutic management of individuals afflicted with inflammatory bowel disease (IBD), including ulcerative colitis (UC) and Crohn’s disease (CD), as well as rheumatologic and dermatological conditions such as rheumatoid arthritis (RA), juvenile idiopathic arthritis, and cancer (Evangelatos et al., 2022). The activation of signal transduction pathways that promote apoptosis can occur in cancer illnesses by the recruitment of death domains (DDs) containing adapters, such as the Fas-associated death domain (FADD) and TNFR-associated DD (TRADD), via TNF signaling (Mak and Yeh, 2002). The activation of transcription factors, such as NF-kappa B and JNK, can be facilitated by the recruitment of TRAF family proteins. This activation promotes cell survival and differentiation, as well as immunological and inflammatory responses. Tumor necrosis factor ligands and receptors were included based on their sequence and structure. The TNF-related ligand is classified as a type II transmembrane protein, characterized by an internal N terminus and an external C terminus, known as the “TNF homology domain” (THD). An essential characteristic of the receptor is the presence of a cysteine-rich domain (CRD), which is composed of three disulfide connections encircling the core motif CXXCXXC, resulting in the formation of an elongated molecule (Suo et al., 2022). Figure 2 illustrates the mechanism by which tumor necrosis factor (TNF) exerts its effects via the tumor necrosis factor receptor (TNFR), hence participating in the extrinsic pathway for induction of apoptosis. The association between the TNFR and procaspases is mediated by adapter proteins such as FADD and TRADD. These adapter proteins have the ability to cleave dormant procaspases, thus initiating the caspase cascade. This cascade ultimately leads to irreversible induction of death in cells (Elmore, 2007). Studies on the apoptotic pathway have identified a malfunction in the breakdown of lysosomal DNA, which triggers macrophages to generate cytokines like IFN^2^ and TNF, without relying on Toll-like receptors (TLRs) (Brouckaert et al., 2004). The interaction between tumor necrosis factor and tumor cells initiates cytolysis, which is the process of cell death. The inflammatory response can be enhanced by tumor necrosis factor (Li and Beg, 2000). The mechanism of action of various phytochemical compounds in the TNF-signaling pathway in cancer is shown in Table 2.

**TABLE 2:** Mechanism of action of various phytochemical compounds in TNF signaling in cancer.

Target	Compound	Types of study	Mechanism of action	Cell line(s)/animal model (s)	References
TNF signaling	Anacardic acid	*In vitro*	Anacardic acid resulted in decreased HaCaT cell viability and increased cell apoptosis and also limited TNF-α-mediated inflammatory responses and downregulated the NF-κB signaling axis	HaCaT cells	Liu et al. (2024)
TNF signaling	Apigenin	*In vitro*	Apigenin prevents SCC25 and A431 cell growth and induces cell cycle arrest in the G2/M phase. It also induces cell apoptosis via TNF-R-, TRAIL-R-, and Bcl-2-mediated caspase in SCC25 cells	SCC25 cell	Chan et al. (2012)
TNF signaling	Butein	*In vitro*	The impact of butein on TNF-α-induced adhesion molecule production in human lung epithelial cells and its molecular mechanism. Butein decreased TNF-α-induced ICAM-1 and VCAM-1 expression, monocyte adhesion, and ROS generation via inhibiting NF-κB, MAPK, and Akt signaling pathways	Human lung epithelial A549 cells and human monocyte leukemia U937 cells	Jang et al. (2012)
TNF signaling	Carnosol	*In vitro*	TNF-α-induced protein production of ICAM-1, VCAM-1, and E-selectin was reduced by carnosol	Human umbilical vein endothelial cells (HUVECs)	Yao et al. (2014)
TNF signaling	Catechin	*In vitro*	Catechin exhibits potential as a therapeutic agent by mitigating the inflammatory response induced by TNF-κ via signaling pathways implicated in inflammation and cytokine activity	T3-L1 preadipocytes	Cheng et al. (2019)
TNF signaling	Gallic acid	*In vitro*	Gallic acid induces aHSC necroptosis via TNF signaling, and oxidative stress can lead to TNF-α generation, leading to necroptosis signaling and necrosome formation (RIP1, RIP3, and caspase-8 inactivation)	Primary hepatic cells (HCs) and hepatic stellate cells (HSCs)	Chang et al. (2015)
TNF signaling	Genistein	*In vitro*	The apoptotic effects of genistein on tumor necrosis factor-α (TNF-α)-induced proliferation in human aortic smooth muscle cells (HASMCs)	Human aortic smooth muscle cells (HASMCs)	Kim et al. (2010)
TNF signaling	Hesperitin	*In vivo*	Hesperetin suppresses NF-kB activation, which drives inflammation and produces pro-inflammatory cytokines like TNF-α, IL-1, and IL-6, leading to anti-tissue injury activity	Male Swiss mice	Zaafar et al. (2022)
TNF signaling	Luteolin	*In vitro*	Luteolin inhibits TNFα-induced apoptosis by inhibiting NF-κB activation, suppressing activation of antiapoptotic genes like A20 and c-IAP1, and enhancing and prolonging JNK activation	Colorectal cancer COLO205 and HCT116 cells and cervical cancer HeLa cells	Shi et al. (2004)
TNF signaling	Piceatannol	*In vitro*	Piceatannol prevented TNF-induced IκBα phosphorylation, p65 phosphorylation, p65 nuclear translocation, and IκBα kinase activity, but did not affect IκBα degradation	Leukemic cell line KBM-5	Ashikawa et al. (2002)
TNF signaling	Quercetin	*In vitro*	Quercetin partially inhibited extracellular regulated kinase, c-jun amino-terminal kinase, and reactive oxygen species, reducing COX-2 levels	The human hepatoma cell line (HepG2)	Granado-Serrano et al. (2012)
TNF signaling	Resveratrol	*In vitro*	Resveratrol, like BMS-345541, inhibited TNF-β-induced NF-κB-mediated gene biomarkers for proliferation, apoptosis, and invasion	The human colon cancer cell line (HCT116)	Buhrmann et al. (2019)
TNF signaling	Xanthohumol	*In vitro*	Xanthohumol boosts TRAIL’s apoptosis and cytotoxicity in prostate LNCaP cancer cells and may induce apoptosis by activating caspases-3, -8, -9, Bid, Bax, Bcl-xL, and mitochondrial potential in LNCaP cells	Human prostate cancer LNCaP cell line	Kłósek et al. (2016)

## 5 Role of phytochemicals in the death receptor pathways

**FIGURE 3:**
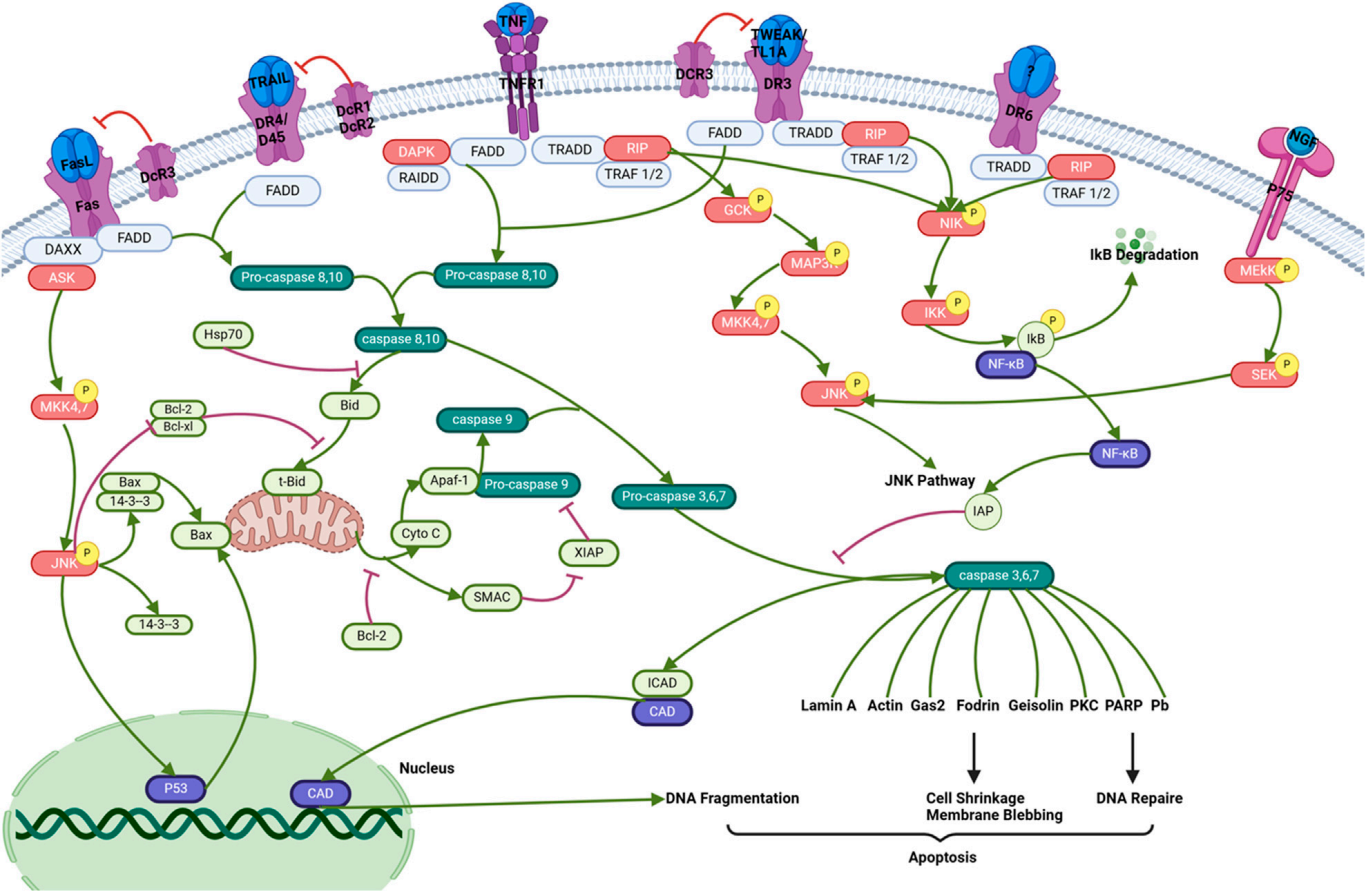
Death receptor pathways in cancer. Activation of these receptors by specific ligands triggers the recruitment of multiple molecules to the death domain, which then initiates a signaling cascade. There are two distinct forms of death receptor signaling complexes. The first group comprises death-inducing signaling complexes (DISCs) that activate caspase-8, a crucial component in apoptotic signaling transduction. DISCs are formed at the CD95, TRAILR1, or TRAILR2 receptors. These processes involve the recruitment of various molecules that facilitate apoptotic and survival signal transduction (https://www.sinobiological.com/pathways).

Death receptors are receptors located on the surface of cells that send signals triggering apoptosis. The involvement of death receptors in the process of apoptosis of inflammatory cells, both *in vivo* and *in vitro*, is of significant importance in various disorders, including inflammation, hypertension, and cancer (Green, 2022). In certain cases ,T lymphocytes and macrophages exhibit the presence of several TNFR/ligands within plaques (Situmorang et al., 2022b). The TNFR pathway has been linked to the death of T cells and macrophages (Situmorang P. C. et al., 2024). These receptors, like TNFR, are stimulated by specific ligands and play a critical role in instructive apoptosis (Siegmund et al., 2016). Death receptors are classified under the superfamily of tumor necrosis factor receptor (TNFR) genes (Hehlgans and Pfeffer, 2005). So far, eight members of the death receptor family have been identified: TNFR1 (also referred to as DR1, CD120a, p55, and p60), CD95 (also referred to as DR2, APO-1, and Fas), DR3 (also referred to as APO-3, LARD, TRAMP, and WSL1), TRAILR1 (also referred to as DR4 and APO-2), TRAILR2 (also referred to as DR5, KILLER, and TRICK2), DR6, ectodysplasin A receptor (EDAR), and nerve growth factor receptor (NGFR). The death receptors can be identified by the presence of a cytoplasmic region known as the death domain (DD), which has around 80 residues (Hoffmann et al., 2009). In the context of cancer (Figure 3), the activation of these receptors by certain ligands leads to the recruitment of several molecules to the death domain, subsequently initiating a signaling cascade. There are two distinct forms of death receptor signaling complexes. The first group comprises death-inducing signaling complexes (DISCs) that lead to the activation of caspase-8, a pivotal component in the signaling transduction pathways that mediate apoptosis (Gnesutta and Minden, 2003). DISCs are generated on the CD95, TRAILR1, or TRAILR2 receptors. The second category consists of TNFR1, DR3, DR6, and EDAR. The process entails the recruitment of several molecules that assist the translation of signals related to apoptosis and cell survival (Schneider-Brachert et al., 2013). The mechanism of action of various phytochemical compounds in the death receptor pathway in cancer is shown in Table 3.

**TABLE 3:** Mechanism of action of phytochemical compounds in the death receptor pathways in cancer.

Target	Compound	Types of study	Mechanism of action	Cell line(s)/animal model(s)	References
Death receptor pathways	Anthraquinone	*In vitro*	Anthraquinone can downregulate various cell survival proteins and induce cell surface expression of both TRAIL receptors, death receptors (DR) 4 and 5, as well as inhibit X-linked apoptotic proteins by siRNA	Human HCC cell line HepG2	Subramaniam et al. (2013)
Death receptor pathways	Allicin	*In vitro*	Allicin reduced cell viability, proliferation, and migration in A549 cells and induced apoptosis and autophagy via ROS buildup and S/G2-M phase arrest in normoxia and hypoxia	Human NSCLC cell lines A549 (adenocarcinoma) and NCI-H460 (large-cell carcinoma)	Pandey et al. (2020)
Death receptor pathways	Aspalathin	*In vitro* and *in vivo*	Aspalathin impacts lipid metabolism, insulin resistance, inflammation, and apoptosis by modulating key regulators (Adipoq, Apob, CD36, Cpt1, Pparγ, Srebf1/2, Scd1, Vldlr, Igf1, Akt1, Pde3, and Map2k1)	Male C57BLKS/J homozygous Leprdb/db mice and embryonic ventricular rat heart-derived H9c2 cardiomyoblasts	Johnson et al. (2017)
Death receptor pathways	Arctigenin	*In vitro*	Arctigenin inhibits the mTOR pathway in ERα-positive human breast cancer cells MCF-7, resulting in autophagy-induced cell death and downregulation of ERα expression	MCF-7 and MDA-MB-231 human breast cancer	Maxwell et al. (2018)
Death receptor pathways	Baicalein	*In vitro*	Baicalein inhibits cell growth and death. Baicalein decreased cyclin B1 and phospho-CDC2 (Thr161) and enhanced G2-M. CDC2 kinase or CDC25 phosphatase inhibitors increased baicalein-induced cytotoxicity	TSGH8301 and BFTC905 cells	Chao et al. (2007)
Death receptor pathways	Bis-eugenol B	*In vitro*	Bis-eugenol B signals cell death by downregulating Bcl-2 and upregulating Bax, which regulates MMP and releases cytochrome c from the mitochondria to the cytoplasm	Prostate cancer cells (PC3) and normal prostate cells (RWPE-)	Abbaspour Babaei et al. (2017)
Death receptor pathways	Britanin	*In vitro* and *in vivo*	The migration ability of tumor cells was significantly weakened after treatment with britanin through inhibiting p65 protein expression and decreasing the Bcl-2/Bax ratio	EL 7402 and HepG2 cells; a BEL 7402-luc subcutaneous tumor model	Li et al. (2020)
Death receptor pathways	Curcumin	*In vitro*	The extrinsic death receptor pathway is activated by curcumin, leading to cell apoptosis in chondrosarcoma. This process is mediated by the actions of curcumin, which result in an increase in p53 expression	The human chondrosarcoma cell line JJ012	Lee et al. (2012)
Death receptor pathways	Celastrol	*In vitro*	Celastrol increases Fas, death domain-associated Fas, TNRSF 1A, and 10B, and death domain-associated TNFRSF1A and reduces the mitochondrial membrane potential dose-dependently	Human NPC cell lines HONE-1 and NPC-039	Lin et al. (2017)
Death receptor pathways	Casticin	*In vitro*	Casticin can induce apoptosis through the activation of caspase-3, -8 and -9; moreover, casticin inhibits the growth of HCC cells regardless of the p53 status	PLC/PRF/5 (p53 mutant) and Hep G2 (p53 wild-type) human HCC cells	Yang et al. (2011)
Death receptor pathways	Dehydrocostus lactone	*In vitro*	Causes G2/M cell-cycle arrest and morphological alterations; increases caspase-3/7, cleaved caspase-3, and PARP function; and decreases ABCB1/MDR1 and ABCG2/BCRP1 expression	SW-985, SW-872, and TE-671	Kretschmer et al. (2012)
Death receptor pathways	Eupafolin	*In vitro*	Eupafolin dose-dependently caused apoptosis, as shown by DNA fragmentation and annexin V-positive cells. Eupafolin also activated caspases-3, -6, -7, -8, and -9 and cleaved their substrates, such as poly (ADP-ribose) polymerase and lamin A/C	Cervical adenocarcinoma HeLa cells	Chung et al. (2010)
Death receptor pathways	Epigallocatechin gallate (EGCG)	*In vitro*	EGCG promotes poly (adenosine diphosphate-ribose) polymerase (PARP) cleavage and induces caspase-8 activation by increasing the expression of death receptor 5 (DR5) at the protein and mRNA levels	SW480 and HCT116 cells	Kwon et al. (2020)
Death receptor pathways	Evodiamine	*In vitro*	Evodiamine increased cyclin B1 and decreased B-cell lymphoma/lewkmia-2 (Bcl-2) and increased Bacx to cause G2/M arrest and cell death	Human ovarian cancer cells HO-8910PM	Wei et al. (2016)
Death receptor pathways	Fisetin	*In vitro*	Fisetin stops autophagic cell death from occurring by blocking mTORC1 expression	Human CaP cell lines PC3, DU145. and LNCaP	Jia et al. (2019)
Death receptor pathways	Kaempferol	*In vitro*	Kaempferol-induced apoptosis and p53 upregulation did not involve Chk2. Extrinsic apoptosis was induced by kaempferol via death receptors/FADD/caspase-8	Human ovarian cancer A2780/CP70 cells	Gao et al. (2018)
Death receptor pathways	Luteolin	*In vitro*	Luteolin significantly increased DR5, Bcl-2-interacting domain cleavage, and caspase-8, -10, -9, and -3 activation. Reducing DR5 expression with siRNA also reduced luteolin-induced caspase activation and apoptosis	Human malignant tumor cells	You et al. (2019)
Death receptor pathways	Rhodomyrtone	*In vitro*	Rhodomyrtone suppressed FAK and serine/threonine AKT, Ras, RhoA, Rac1, and Cdc42 phosphorylation and lowered MMP-2 and MMP-9 protein and enzyme activity in SW1353 cells	Human chondrosarcoma SW1353 cells	Tayeh and Watanapokasin (2020)
Death receptor pathways	Shikonin	*In vitro* and *in vivo*	Cells treated with 3-MA and shikonin showed enhanced expression of cleaved PARP, caspase-3, and RIP1, suggesting that autophagy protects cells	A549 human lung cancer cells; 5–6-week-old BALB/c athymic nude mice	Kim et al. (2017)
Death receptor pathways	Thiosulfinates	*In vitro*	Thiosulfinates increase Bid cleavage, showing that caspase-8-mediated apoptosis activates caspase-9. Thiosulfinates decreased Bcl-2 expression and increased Bax expression	HT-29 human colon cancer cells	Guillamón et al. (2023)
Death receptor pathways	Xanthone	*In vitro*	Xanthone extract or nanoemulsion can halt the cell cycle at the S phase in HepG2 cells, causing a larger proportion of late apoptotic cells and increased caspase-3, caspase-8, and caspase-9 activity	HepG2 cells	Li et al. (2023)

## 6 Role of phytochemicals in the p53 pathway

**FIGURE 4:**
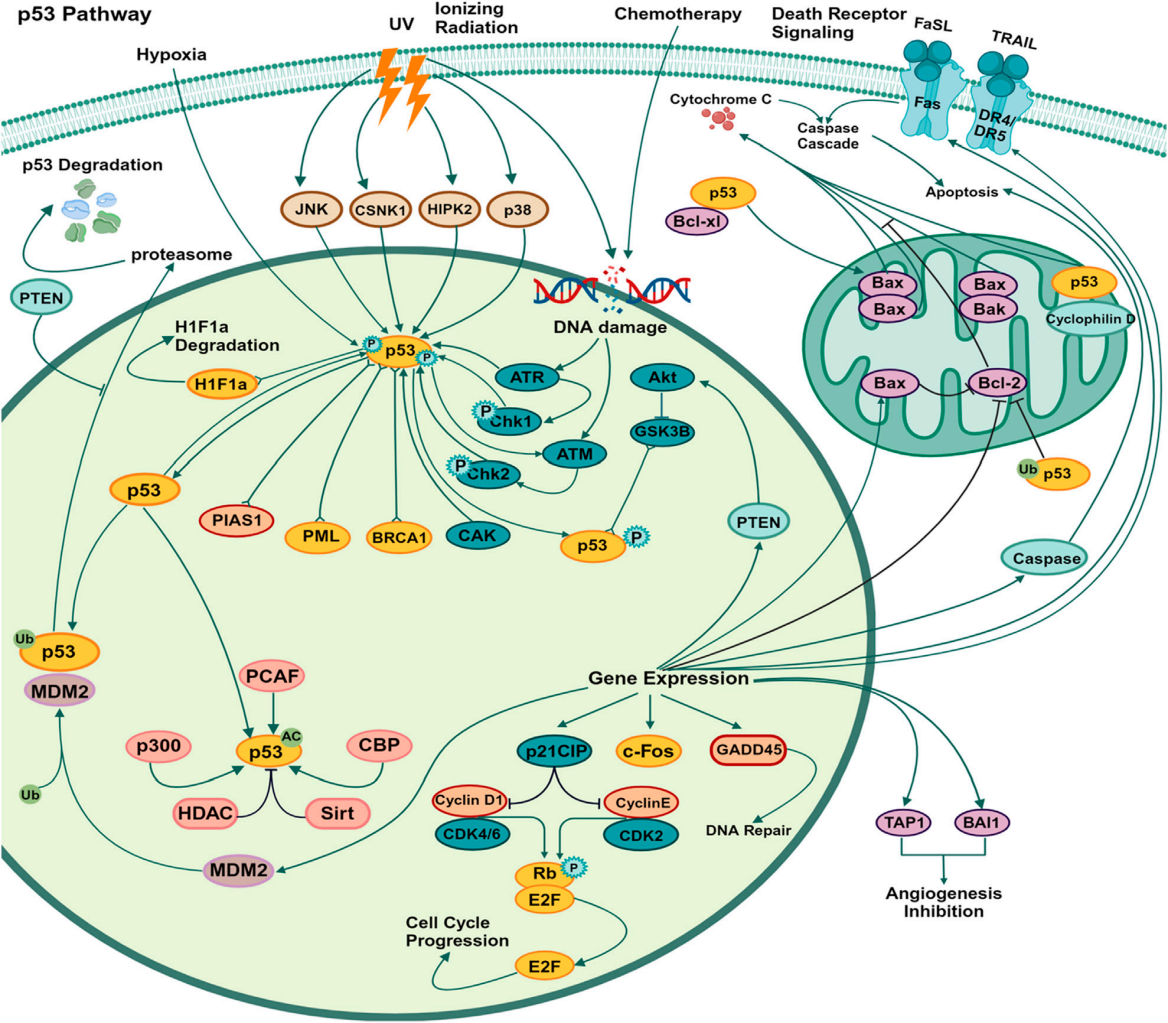
p53 pathways in cancer. The p53 mechanism occurs due to various stress stimuli, such as DNA damage, nucleolar stress, metabolic stress, and oncogenic stress. p53 protein levels show stability. Proteasome-dependent degradation of p53 is facilitated by E3 ligases, which enhance ubiquitination, and cytoplasmic connections between p53 and Bcl-2 family proteins have also been observed to promote apoptosis (https://www.sinobiological.com/pathways).

The p53 tumor suppressor is a prominent mechanism involved in apoptosis signaling. Cell loss in various neurological illnesses, such as Alzheimer’s disease, Parkinson’s disease, hypertension, stroke, and cancer, may be attributed to p53-related apoptosis, which is a frequently observed process (Wolfrum et al., 2022). In response to genotoxic or cellular stress, the p53 protein functions as a nuclear transcription factor, exerting regulatory control over the expression of several genes associated with apoptosis, growth arrest, or senescence (Mijit et al., 2020). E3 ubiquitin ligases, such as MDM2, exert a negative regulatory effect on p53 protein levels in cancer (Figure 4). The proteasome-dependent degradation of p53 is facilitated by the E3 ligase, which enhances ubiquitination. In response to various stress stimuli, such as DNA damage, nucleolar stress, metabolic stress, and oncogenic stress, the levels of p53 protein exhibit stability. The cytoplasmic connections between P53 and Bcl-2 family proteins have been observed to facilitate the process of apoptosis. It was demonstrated in the late 1980s and early 1990s that the introduction of the wild-type p53 gene into different human tumor cells resulted in the induction of apoptosis and suppression of cell growth (Dhokia et al., 2022). A curative response was observed in a mouse model system where the function of p53 was precisely reactivated in the tumor. The activation of p53 triggers cell-cycle arrest, facilitating DNA repair and/or apoptosis to inhibit the proliferation of cells with significant DNA damage (Chen, 2016). This is achieved via transactivating target genes that are involved in the initiation of cell cycle arrest and/or apoptosis. The mechanism of action of various phytochemical compounds in the p53 pathway in cancer is shown in Table 4.

**TABLE 4:** Mechanism of action of phytochemical compounds in the p53 pathway in cancer.

Target	Compound	Types of study	Mechanism of action	Cell line(s)/animal model (s)	References
P53	Ascochlorin	*In vitro*	The inhibition of mitochondrial respiration is a consequence of the activation of p53 by ascochlorin, a phenomenon that is corroborated by the fact that respiratory inhibitors elicit p53 activation in a way akin to that of ascochlorin	U2OS (human osteosarcoma)	Jeong et al. (2009)
P53	Apigenin	*In vitro*	Apigenin decreased the tyrosine phosphorylation of HER2 and increased the levels of p53, phospho-p53, and p21 in both MCF-7 vec and MCF-7 HER2 cells. Furthermore, apigenin was found to cause apoptosis through the p53-dependent pathway	Her2 (breast cancer)	Rahmani et al. (2022)
P53	Butein	*In vitro*	The process of apoptosis induced by butein is facilitated by the p53 protein, causes the arrest of KBM5 cells in the S phase, and changes the levels of specific cyclins and downstream targets of p53, namely, MDM2 and p21	The KBM5 and K562 cell lines	Woo et al. (2016)
P53	Chalcones	*In vitro*	Chalcone increased p53 expression in MCF-7 cells, suggesting that this chemical had the capability to stimulate and maintain the stability of p53 protein expression	The MCF-7 line represents estrogen receptor-positive breast cancer cells, while the MDA-MB-231 line represents triple-negative breast cancer (TNBC) cells	Dos Santos et al. (2019)
P53	Curcumin	*In vitro*	Curcumin stimulates the P53 signaling pathway and inhibits the PI3K signaling pathway to promote gastric cell death and autophagy by upregulating P53 and P21	Two cell lines, SGC-7901 and BGC-823 (gastric cells)	Fu et al. (2018)
P53	Epigallocatechin-3-gallate (EGCG)	*In vitro* and *In vivo*	EGCG decreases β-catenin mRNA and transcriptional activity in cells via the p53 pathway and increases ubiquitin-mediated 26S proteasomal degradation	Human HNC cell lines (oral cavity squamous cell carcinoma and a syngeneic mouse model	Kciuk et al. (2023)
P53	Formononetin	*In vitro*	Formononetin dose- and time-dependently reduced A549 cell growth and caused apoptosis. miR-27a-3p suppressed HIPK2 3′UTR expression. In formononetin-treated A549 cells, miR-27a-3p expression decreased, that of HIPK2 increased, and p53 decreased	Human non-small-cell lung cancer (NSCLC)	Hu and He (2021)
P53	Garcinol	*In vitro*	Garcinol blocked CBP/p300-mediated acetylation of the p53’s C-terminal activation domain but boosted p53K120 acetylation and cytoplasmic p53 accumulation. Moreover, DNA damage signaling markers such as γH2A.X, H3K56Ac, p53, and TIP60 were upregulated	MCF7 cells	Collins et al. (2013)
P53	Honokiol	*In vitro*	Honokiol treatment of human H4 neuroglioma cells caused cell death and downregulated cyclin B1, CDC2, and cdc25C expression but increased p-CDC2 and p-cdc25c expression. Honokiol also elevated p53, p21, and Bax/Bcl-2 expression. The molecular mechanism involves activating p53 signaling and arresting the cell cycle	H4 human neuroglioma cells	Guo et al. (2015)
P53	Resveratrol	*In vitro*	Resveratrol activates the tumor suppressor p53 and exhibits p53-independent apoptosis by reducing the expression of phosphorylated Akt-mediated NF-κB suppression, as also evidenced by the downregulation of antiapoptotic factors Bcl-2 and Bcl-xl	A549 and HCC-15 cells	Jang et al. (2022)
P53	Silibinin	*In vitro* and *in vivo*	Silibinin effectively repaired UVB-induced DNA damage in p53+/+ mice but was less effective in p53−/− animals. Activating p53 helps silibinin protect against UVB-induced photodamage, inflammation, and photocarcinogenesis by inhibiting skin tissue indicators of UVB-induced inflammation	Non-melanoma skin cancers (NMSC), p53−/− male breeders of the C57BL/6 strain and female breeders of the SKH-1 hairless strain	Rigby et al. (2017)
P53	Quercetin	*In vitro*	Quercetin induces p53-dependent G2/M phase cell-cycle arrest and mitochondrial apoptosis to reduce HeLa cell viability	Human cervical cancer (HeLa)	Son and Kim (2023)

## 7 Role of phytochemicals in the p38 pathway

**FIGURE 5:**
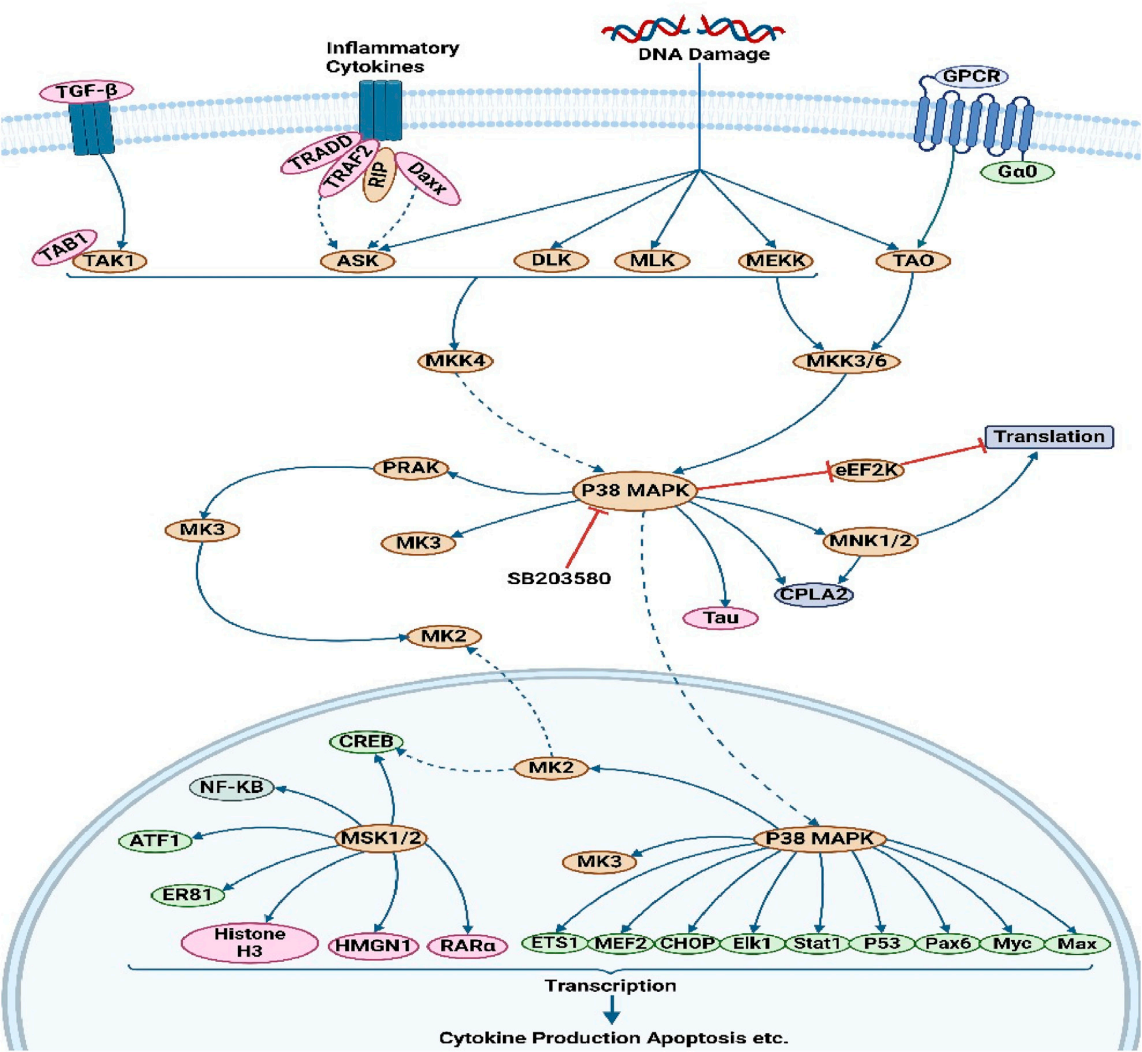
p38 pathways in cancer. p38 MAPK governs the regulation of downstream targets, including different kinases, transcription factors, and cytosolic proteins. The kinases included in this study are MAPKAPK2, MAPKAPK3, PRAK, MSK1, and MNK ½. P38 phosphorylates various important transcription factors, including the tumor suppressor protein p53, CHOP (C/EBP homologous protein), STAT1 (signal transducer and activator of transcription-1), CREB (cAMP response element-binding protein), and Max/Myc complex. The p38 MAPK pathway is essential for controlling the synthesis of pro-inflammatory cytokines at both the transcriptional and translational stages (https://www.sinobiological.com/pathways).

The involvement of p38 MAP kinase (MAPK) in signaling cascades governing cellular reactions to cytokines and stress has been observed (Falcicchia et al., 2020). Four p38 MAP kinases have been found in mammals, namely, p38-α (MAPK14), p38-β (MAPK11), p38-γ (MAPK12/ERK6), and p38-ι (MAPK13/SAPK4). Just like the SAPK/JNK pathway, the activation of p38 MAP kinases occurs in response to several cellular stressors and various illnesses, including osmotic shock, inflammatory cytokines, lipopolysaccharide (LPS), UV light, growth factors, hypertension, and cancer (Asih et al., 2020). Furthermore, the activation of p38 is indirectly induced by oxidative stress and GPCR stimulation (Situmorang et al., 2023). The regulation of downstream targets, such as various kinases, transcription factors, and cytosolic proteins, is governed by p38 MAPK in the context of cancer (Huang et al., 2024). The kinases considered in this study are MAPKAPK2, MAPKAPK3, PRAK, MSK1, and MNK ½ (Figure 5). P38 phosphorylates several crucial transcription factors, such as the tumor suppressor protein p53, CHOP (C/EBP homologous protein), STAT1 (signal transducer and activator of transcription-1), CREB (cAMP response element-binding protein), and Max/Myc complex (Koul et al., 2013). The p38 MAPK pathway plays a crucial role in regulating the production of pro-inflammatory cytokines at both the transcriptional and translational levels. Consequently, several elements within this system hold promise as possible therapeutic targets for autoimmune and inflammatory disorders (Ganguly et al., 2023). The p38 pathway plays a crucial role in controlling cell growth and suppressing tumor growth by influencing many regulators of the cell cycle. The tumor-suppressing role of the p38 pathway suggests that several elements of the p38 pathway hold promise as possible targets for innovative cancer treatments (Martínez-Limón et al., 2020). The mechanism of action of various phytochemical compounds in the p38 pathway in cancer is shown in Table 5.

**TABLE 5:** Mechanism of action of phytochemical compounds in the p38 pathway in cancer.

Target	Compound	Types of study	Mechanism of action	Cell line(s)/animal model (s)	References
P38	Astaxanthin	*In vitro*	Astaxanthin-induced downregulation of p38 MAPK XPC enhances erlotinib-induced cytotoxicity in A549 and H1975 cells	Non-small-cell lung cancer (NSCLC) cell	Chen et al. (2018)
P38	α-Mangostin	*In vitro* and *in vivo*	α-Mangostin inhibited tumor growth in cervical cancer mouse xenograft models by increasing p-ASK1, p-p38, cleaved-PARP, and cleaved-caspase-3 and inhibiting cell viability. This led to loss of mitochondrial membrane potential (MMP), release of cytochrome C, increase in Bax, decrease in Bcl-2, and activation of the caspase	Human cervical cancer cell line SiHa (ATCC HTB35); female nude mice (BALB/c nu/nu) as xenograft animal models	Lee et al. (2017)
P38	Baicalein	*In vitro*	Apoptosis, induced by caspase activation, downregulation of bcl-2, and overexpression of bax or p53 via the ERK/p38 MAPK pathway inhibits growth	Human breast cancer MCF-7 and MDA-MB-231	Zhou et al. (2009)
P38	Cardamonin	*In vitro*	The impact of cardamonin on the proliferation and death of normal cells was not readily apparent. In addition, it inhibited the proliferation of OS cells in a xenograft mice model and elevated the phosphorylation threshold of P38 and JNK.	Human OS cell lines 143B and MG63, as well as human normal brain glial cell HEB, human normal bone marrow stromal cell HS5, and human normal fetal hepatocyte LO2	Zhang et al. (2021a)
P38	Casticin	*In vitro*	Casticin decreases cell viability and arrests the G2/M cell cycle via activating caspases 8, 9, and 3 and activating endogenous p38 MAPK in untreated cells based on phospho-MAPK expression levels	HL-60 cells	Kikuchi et al. (2013)
P38	Curcumin	*In vitro*	Inhibitors that downregulate ERK and p38 MAPK phosphorylation did not have any impact on curcumin-induced apoptosis. However, the use of shRNA to knock down p38 MAPK dramatically decreased curcumin-induced apoptosis	U0126 and SB203580 (lung cancer)	Wu et al. (2022b)
P38	Epigallocatechin-3-gallate (EGCG)	*In vitro*	EGCG inhibited proliferation and migration of OVCAR-3 cells by reducing p38 phosphorylation potentially mediated through the activation of p38 MAPK and downregulation of MMP2 protein expression	The OVCAR-3 human ovarian adenocarcinoma cell line	Wang et al. (2014b)
P38	Formononetin	*In vitro*	Formononetin contributes to a decrease in Bcl-2 protein levels and an increase in Bax expression in PC-3 cells, thereby resulting in an increase in the Bax/Bcl-2 ratio and regulating the p38/Akt pathway, thereby triggering apoptosis in tumor cells	Prostatic adenocarcinoma (PC-3) and human prostate epithelial cells (RWPE1)	Almatroodi et al. (2023)
P38	Garcinol	*In vitro*	The p38-MAPK inhibitor and garcinol synergistically increase the expression of cyclin E, p21Waf1/Cip1, and p27Kip1 and induce G1 cell cycle arrest and apoptosis in lung cancer cells	H1299 lung cancer	Pai et al. (2021)
P38	Honokiol	*In vitro*	Honokiol induces excessive ROS and thus does not affect Lip-HNK-induced apoptosis, but is also associated with inhibition of the ERK/p38-MAPK signaling pathway	D283, DAOY, BV2, and HT22	Li et al. (2022)
P38	Resveratrol	*In vitro*	Resveratrol upregulated SIRT1 and inhibited Akt/mTOR while activating p38-MAPK in NSCLC cells dose-dependently, possibly causing autophagy. Activating the Akt/mTOR pathway with IGF-1 or blocking the p-38-MAPK pathway greatly reduces cell proliferation and increases apoptosis	Non-small-cell lung cancer (NSCLC)	Wang et al. (2018)
P38	Silibinin	*In vitro*	Two ROS scavengers reduced p38/p-p38 expression and NF-κB transposition from the cytoplasm to the nucleus, while p38 and NF-κB inhibitors and H2O2 scavengers jointly reduced ROS production and silibinin-induced autophagy	The human fibrosarcoma HT1080 cells	Duan et al. (2011)
P38	Quercetine	*In vitro*	Quercetin’s apoptotic effects involve the ROS/AMPKα1/ASK1/p38 signaling pathway, with AMPKα1 playing a crucial role in ASK1-induced apoptosis. Blocking AMPKα1 activity with compound C, synthetic inhibitors, or siRNA prevented quercetin-activated ASK1 from stimulating p38 activity	MCF-7 cells (breast cancer)	Biswas et al. (2022)
P38	Vitisin A	*In vitro*	Vitisin A reduced LPS-induced ERK1/2 and p38 phosphorylation and NF-κB activation. Vitisin A may decrease NO generation by inhibiting ERK1/2, p38, and NF-κB signaling pathways	RAW 264.7 cells	Chang et al. (2017)

## 8 Role of phytochemicals in the actin dynamics signaling pathways

**FIGURE 6:**
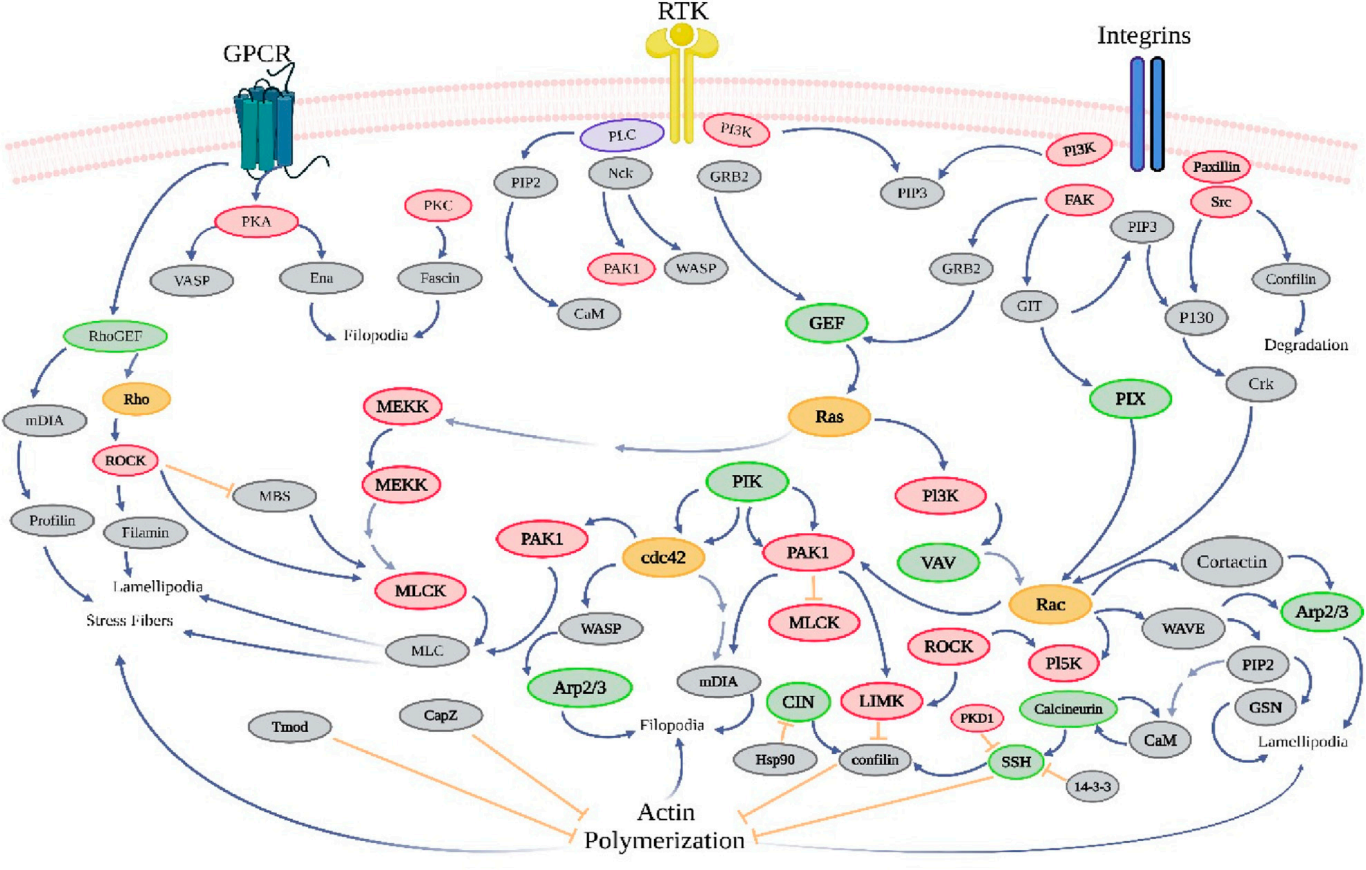
Actin dynamics signaling pathways in cancer. The large protein tyrosine kinase family includes receptor tyrosine kinases (RTKs). Tyrosine kinase receptors, such as EGFR, PDGFR, MCSFR, IGF1R, INSR, NGFR, FGFR, VEGFR, and HGFR, modulate intracellular signals that control the cell’s response to external stimulus and are part of the Ras superfamily of tiny GTP-binding proteins that are involved in many biological processes (https://www.sinobiological.com/pathways).

G protein-coupled receptors (GPCRs), integrins, and receptor tyrosine kinases (RTKs) are involved in the regulation of actin dynamics by extracellular signals. G protein-coupled receptors (GPCRs) encompass a diverse group of protein receptors that detect extracellular chemicals and initiate intracellular signal transduction pathways, ultimately leading to physiological responses (Cheng et al., 2023). The formation of aberrant thin filaments and the disruption of muscular contraction might result from dysfunctional actin–ATP binding, hence causing muscle weakening and various manifestations of actin accumulation myopathy (Dowling et al., 2021). No ACTA1 gene mutation has been detected in certain individuals with actin accumulation myopathy. In addition to muscular issues, actin dynamics signaling pathways also contribute to the development of cancer. Transmembrane receptors known as integrins serve as intermediaries between cell–cell interactions and the extracellular matrix (ECM) of a cell. In cancer, integrins initiate signaling transduction pathways that affect the cell interior, including the chemical composition and mechanical state of the extracellular matrix (ECM) (Jo et al., 2020). This leads to transcriptional activation, which in turn regulates several cellular processes such as cell cycle regulation, cell shape, the cell’s ability to move, and the addition of additional receptors to the cell membrane (Pang et al., 2023). RTKs, or receptor tyrosine kinases, belong to the extensive group of protein tyrosine kinases. Tyrosine kinase receptors encompass a diverse array of proteins, including EGFR, PDGFR, MCSFR, IGF1R, INSR, NGFR, FGFR, VEGFR, and HGFR (Figure 6). Rho is responsible for modulating intracellular signals that control the cell’s response to external stimulus. Rho is a constituent of the Ras superfamily of tiny GTP-binding proteins that has significant involvement in various biological processes, including the organization of the actin cytoskeleton, dynamics of microtubules, transcription of genes, transformation of cancer cells, development of the cell cycle, adhesion, and epithelial wound repair (Zubor et al., 2020). The activation of GEF (guanine nucleotide exchange factor) is seen (Ilyas et al., 2022). The protein kinase effectors ROCK and PAK are located downstream. The occurrence of immunological diseases, developmental abnormalities, and cancer often involves the disruption of cytoskeletal signaling, leading to the cessation of production of extracellular stimuli and cellular responses (Miller and Zachary, 2017). The mechanism of action of various phytochemical compounds in actin dynamics signaling pathways in cancer is shown in Table 6.

**TABLE 6:** Mechanism of action of phytochemical compounds in actin dynamics signaling in cancer.

Target	Compound	Types of study	Mechanism of action	Cell line(s)/animal model (s)	References
Actin dynamics pathways	Curcumin	*In vitro*	Curcumin inhibits lung cancer cell migration and invasion by inhibiting the Rac1/PAK1 signaling pathway and MMP-2 and MMP-9 expression, providing new molecular insights	Human lung cancer cells	Chen et al. (2014a)
Actin dynamics pathways	Epi-gallocathechin-3-gallate	*In vitro*	EGCG reduced Rho A activation in dominant-negative Rho A N19 cells and constitutively active Rho A Q63E cells and inhibited the invasive ability of the cells	Oral squamous-cell carcinoma	Kciuk et al. (2023)
Actin dynamics pathways	Fisetin	*In vitro*	Fisetin suppresses the survival and formation of colonies and reduces the expression of P-gp in the NCI/ADR-RES multidrug-resistant cancer cell line, as well as inhibits cell proliferation, invasion, and migration	Prostate cancer cells	Mukhtar et al. (2015)
Actin dynamics pathways	Migrastatin	*In vitro*	Metastasis in a mice breast tumor model and a human small-cell lung carcinoma model	A human small-cell lung carcinoma (SCLC)	Lecomte et al. (2011)
Actin dynamics pathways	Resveratrol	*In vitro*	Resveratrol promotes Rac activity in breast cancer cells by expressing dominant-negative Cdc42 or Rac and preserving filopodia responsiveness, so Rac and Cdc42 may regulate actin cytoskeleton signaling differently at low and high concentrations	Breast cancer cells	Azios et al. (2007)
Actin dynamics pathways	silibinin	*In vitro*	Silibinin impairs mitochondrial dynamics and biogenesis, thereby reducing migration and invasion of MDA-MB-231 breast cancer cells	MDA-MB-231 and MCF-7 cells	Si et al. (2020)
Actin dynamics pathways	Quercetin	*In vitro*	Quercetin targets and pathways are seven proteins (HMOX1, ACE, MYC, MMP9, PLAU, MMP3, and MMP1) that can influence the JNK pathway, glycolysis, and epithelial–mesenchymal transition (EMT) that can regulate MMP9 expression	LoVo human colon cancer cells	Zhou et al. (2023)
Actin dynamics pathways	Wiskostatin	*In vitro*	Integrin-dependent migration of NK cells toward CXCL12/SDF-1and CX3CL1/fractalkine	β1+/+, β1–GFP, β1−/−, and β3−/− cells	King et al. (2011)

## 9 Role of phytochemicals in the autophagy pathways

**FIGURE 7:**
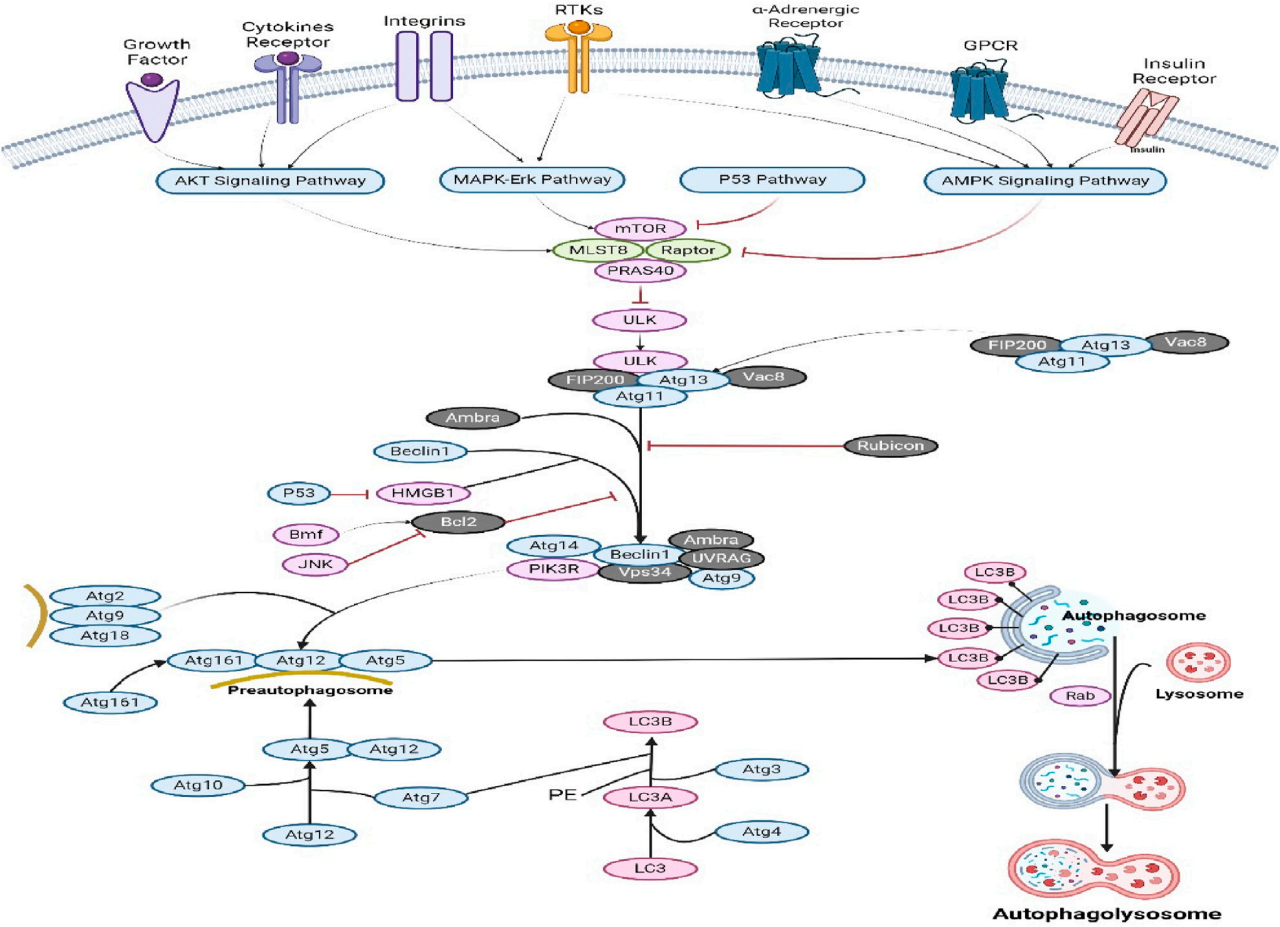
Autophagy pathways in cancer. Activating mTOR (Akt and MAPK signaling) in cancer suppresses autophagy, while negative regulation (AMK and p53 signaling) enhances it. Both autophagy and apoptosis have beneficial and negative effects, and they communicate. Under nutritional constraint, autophagy aids survival. Other pro-apoptotic signals including TNF, TRAIL, and FADD trigger autophagy (https://www.sinobiological.com/pathways).

Autophagy is a cellular recycling mechanism characterized by the dynamic destruction of cytoplasmic contents, aberrant protein aggregates, and excess or damaged organelles through the autophagosome–lysosome process (Gómez-Virgilio et al., 2022). Impaired autophagy function is linked to NAFLD, diabetes, AKD/CKD, heart failure, IBD, and neurodegenerative disorders. These models provide comprehensive data on initial effectiveness, toxicity, pharmacokinetics, and safety, which aids in determining whether a molecule should be further developed for the purpose of conducting clinical studies (Situmorang et al., 2021b; Situmorang et al., 2021c). Nevertheless, in the context of cancer, both the inhibition and augmentation of autophagy significantly contribute to the advancement of the disease via several mechanisms. The activation of mTOR (Akt and MAPK signaling) in cancer leads to the suppression of autophagy, while the negative regulation of mTOR (AMK and p53 signaling) increases autophagy (Verma et al., 2021). ULK functions in a manner comparable to that of yeast Atg1, operating in the downstream region of the mTOR complex. The formation of a substantial complex between ULK, Atg13, and the scaffolding protein FIP200 is observed. The induction of autophagy necessitates the presence of the class III PI3K complex, which comprises hVps34, beclin 1 (the mammalian counterpart of yeast Atg6), p150 (the mammalian counterpart of yeast Vps15), and Atg14-like protein (Atg14L or Barkor) or ultraviolet irradiation resistance-associated gene (UVRAG) (Tran et al., 2021). Rubicon exerts inhibitory effects on the activity of PI3K class III lipid kinase and counteracts the effects of Atg14L, a protein that enhances class III PI3K activity. Autophagosome formation is regulated by the Atg12-Atg5 and LC3-II (Atg8-II) complexes, which are controlled by the Atg gene. Atg12 undergoes conjugation with Atg5 through an ubiquitin-like process, which necessitates the involvement of Atg7 and Atg10 (enzymes with E1-and E2-like properties, respectively). The Atg12–Atg5 conjugate subsequently has a noncovalent interaction with Atg16, resulting in the formation of a substantial complex (Hu and Reggiori, 2022). The protease Atg4 cleaves the C terminus of the second complex, LC3/Atg8, resulting in the formation of cytosolic LC3-I. The process of conjugating LC3-I to phosphatidylethanolamine (PE) involves a ubiquitin-like reaction that necessitates the involvement of Atg7 and Atg3, which are enzymes with E1-and E2-like properties, respectively (Agrotis et al., 2019). LC3-II, a lipidated variant of LC3, adheres to the membrane of autophagosomes (Figure 7). Autophagy and apoptosis have both positive and negative associations, with significant intercommunication observed between these two biological processes. The process of autophagy is a survival strategy that is activated when nutrients are scarce (Xi et al., 2022). However, an excessive amount of autophagy can result in cell death, which is a separate process from apoptosis in terms of its morphology. Autophagy is also induced by other pro-apoptotic signals, including TNF, TRAIL, and FADD. Furthermore, Bcl-2 exerts inhibitory effects on beclin 1-dependent autophagy (Ilyas et al., 2021), thus serving as a dual regulator of both pro-survival and anti-autophagic processes. The mechanism of action of various phytochemical compounds in autophagy pathways in cancer is shown in Table 7.

**TABLE 7:** Mechanism of action of phytochemical compounds in autophagy pathways in cancer.

Target	Compound	Types of study	Mechanism of action	Cell line(s)/animal model (s)	References
Autophagy	Ampelopsin	*In vitro*	Ampelopsin can inhibit cell viability and metastasis in RCC in a dose-dependent manner by negatively regulating the PI3K/AKT signaling pathway	Normal kidney cells HK-2 and human RCC cells 786-O	Zhao et al. (2021)
Autophagy	Acacetin	*In vitro*	Acacetin exerts inhibitory effects on the invasion, migration, and epithelial–mesenchymal transition (EMT) of gastric cancer cells via the PI3K/Akt/Snail pathway	MKN45 and MGC803 cells	Zhang et al. (2022)
Autophagy	Berberine	*In vitro*	Berberine had a substantial inhibitory effect on the PI3K/AKT/mTOR signaling pathway in BGC-823/DDP and SGC-7901/DDP cells that were subjected to cisplastin treatment	BGC-823 and SGC-7901 cells	Kou et al. (2020)
Autophagy	Baicalein	*In vitro*	The cell cycle modulation and ER activation of gastric cancer cells by baicalein inhibited cell growth, induced G0/G1 arrest, and apoptosis. Baicalein inhibited the PI3K/AKT pathway by activating BTG3, causing ER and apoptosis	GC cells (HGC-27 and AGS)	Shen et al. (2023)
Autophagy	Cyanidin	*In vitro*	The proliferation of cervical cancer cells is inhibited, and the PI3K/AKT/mTOR pathway is downregulated by cyanidin and cisplatin	HeLa cells	Li et al. (2021)
Autophagy	Curcumin	*In vitro*	The expression of critical genes and proteins in the PI3K–AKT–mTOR signaling pathway was downregulated by curcumin in inhibiting the growth and progression of hepatocellular carcinoma (HNC) and in modulating the PI3K–AKT–mTOR pathway	Hypopharynx carcinoma (FaDu), tongue carcinoma (SCC-9), and keratinocyte (HaCaT) cell lines	Borges et al. (2020)
Autophagy	EGCG	*In vitro* and *in vivo*	The efficacy of EGCG in suppressing the proliferation and migration of T24 and 5637 cells was demonstrated, providing evidence that EGCG exerted its inhibitory effects on cell proliferation and tumor formation through the regulation of the PI3K/AKT pathway	Female BALB/c mice and T24 or 5637 cells	Luo et al. (2018)
Autophagy	Isorhamnetin	*In vitro*	The proliferation of cells from all three cell lines was inhibited by isorhamnetin, which also induced cell cycle arrest at the G2/M phase. This suppression of cell proliferation was achieved through the inhibition of the PI3K Akt mTOR pathway	The human CRC cell lines, HT-29, HCT116, and SW480	Li et al. (2014)
Autophagy	Luteolin	*In vitro*	The pretreatment of luteolin decreased the activation of the PI3K-Akt pathway, which is responsible for the reduction of E-cadherin produced by TGF-β1	The lung adenocarcinoma A549	Chen et al. (2013a)
Autophagy	Lupiwighteone	*In vitro*	Lupiwighteone caused caspase-dependent apoptosis (upregulation of caspase-3, -7, -8, -9, PARP, and Bax) and caspase-independent apoptosis which inhibited the PI3K/Akt/mTOR signaling pathway (downregulation of PI3K, p-Akt, and p-mTOR)	MCF-7, an estrogen receptor (ER)-positive human breast cancer cell, and MDA-MB-231, a triple-negative human breast cancer cell	Shiau et al. (2022)
Autophagy	Kaempferol	*In vitro*	Kaempferol resulted in a reduction in cell viability and the initiation of cellular apoptosis and aging by downregulating the PI3K/AKT and hTERT pathways	HeLa cell	Kashafi et al. (2017)
Autophagy	Myricetin	*In vitro*	Myricetin inhibits the proliferation of four colon cancer cell lines by inhibiting the PI3K/Akt/mTOR signaling pathway, which induces cell death and autophagy. Additionally, 3 MA suppresses autophagy, which induces apoptosis in colon cancer cells treated with myricetin	Four human colorectal cancer cell lines, HT-29, HCT116, SW480, and SW620	Zhu et al. (2020)
Autophagy	Matrine	*In vitro*	Matrine suppressed the proliferation of MCF-7/ADR cells, triggered apoptosis, and counteracted the development of multidrug resistance in breast cancer cells. This was achieved via modulating the downstream apoptotic components of the PI3K/AKT signaling pathway, resulting in a reduction in the phosphorylation of AKT levels in the cells	MCF-7/ADR cell	Zhou et al. (2018)
Autophagy	Parthenolide	*In vitro* and *in vivo*	Parthenolide has the potential to impede the growth of lung cancer cells by blocking the PI3K/Akt/FoxO3γ signaling pathway mediated by IGF-1R	Human NSCLC cell lines A549 and H1299 cells; mouse subcutaneous xenografts (male BALB/c nude mice)	Sun et al. (2020)
Autophagy	Pelargonidin	*In vitro* and *in vivo*	Pelargonidin inhibits the PI3K/AKT/mTOR pathway and decreases MMP2, MMP9, N-cadherin, and VEGF protein expression and also inhibits the PI3K/AKT/mTOR pathway, preventing glioma vascularization and metastasis	Adult male Sprague–Dawley rats, the rat glioma cell line C6 (FH0406), and HUVEC (FH1122)	Tian et al. (2022)
Autophagy	Silymarin	*In vitro*	Si-SeNPs increased Bax/Bcl-2, cytochrome c, and caspase protein cleavage in AGS cells, which is linked to mitochondria-mediated apoptosis, and inhibited PI3K/AKT/mTOR pathways were substantially linked with autophagy and apoptotic signaling in AGS cells	AGS gastric cancer cells	Mi et al. (2022)
Autophagy	Silibinin	*In vitro*	Silibinin demonstrates anticancer properties via downregulating the actin cytoskeleton and PI3K/Akt pathways, hence inhibiting the growth and progression of bladder cancer	T24 and UM-UC-3 human bladder cancer cells	Imai-Sumida et al. (2017)
Autophagy	Triptolide	*In vitro*	Triptolide decreased osteoclast bone resorption and RANKL-induced osteoclastogenesis, and PI3K-AKT-NFATc1 is a key downstream pathway of RANKL-induced osteogenesis. NFATc1 overexpression and AKT phosphorylation can mitigate this impact	Bone marrow mononuclear cells (BMMCs)	Cui et al. (2020)
Autophagy	Tocopherol	*In vitro*	Tocopherol-associated protein suppressed tumors by downregulating PI3K/Akt signaling, not cell-cycle arrest or androgen receptor signaling, but lowered Akt activity by inhibiting PI3K subunit interaction, p110-p85	LNCaP, PC-3, DU-145, and RWPE-1 cells	Ni et al. (2005)
Autophagy	Resveratrol	*In vitro*	Resveratrol decreased the protein expression levels of cyclin D1, cyclin E2, and BCL2 apoptosis regulator, while increasing BCL2-associated X and tumor protein p53, which regulate the cell cycle and apoptosis	The human colon cancer cell lines DLD1 and HCT15	Li et al. (2019)
Autophagy	Quercetin	*In vitro*	The bioflavonoid quercetin inhibits the PI3K-Akt/PKB pathway in PTEN-null cancer cells at pharmacologically safe concentrations, suggesting it may treat carcinogenesis and progression	HCC 1937, which exhibits a homozygous deletion of the PTEN gene, and T47D, which possesses an intact PTEN gene	Gulati et al. (2006)
Autophagy	Withanolides	*In vitro*	Withanolides decrease the activity of kinases like PI3K, PKB, mTOR, ERK1/2, and ARAF while increasing DNA repair kinases and modulating oncogenic pro-survival factors	The AML cell lines that were used are HL60, Kasumi-1, and P31/FUJ.	Akhtar et al. (2020)

## 10 Phytochemicals in clinical trials as anticancer agents

**FIGURE 8:**
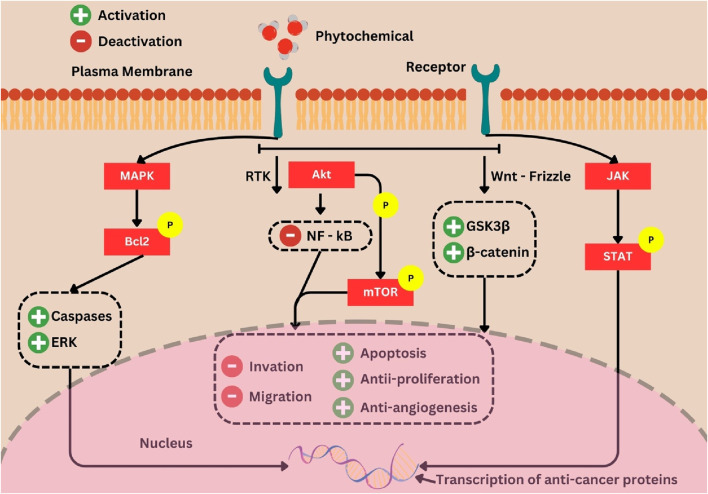
Phytochemicals as chemo-preventives and signaling molecule modulators. In the presence of phytochemicals that increase p53, decrease AKT, and cause cellular apoptosis and cell cycle arrest, tumor suppressor proteins activate the apoptotic cascade. Crosstalk between the p53, MAPK, and JNK pathways also contributes, where the AKT and mTOR pathways are downregulated in cancer cells, causing autophagy, but the presence of herbal phytochemicals can control inflammation, angiogenesis, invasion, and metastasis (Ahmed et al., 2022).

During the practical process of drug development, employing meticulous preclinical screening models can generate promising lead compounds for the development of anticancer drugs. These models provide comprehensive data on initial effectiveness, toxicity, pharmacokinetics, and safety, which aids in determining whether a molecule should be further developed for the purpose of conducting clinical studies (Situmorang et al., 2021b). In the present review, a substantial body of evidence pertaining to the effectiveness of several phytochemicals has been amassed. Clinical research has demonstrated that *P. ginseng* effectively decreases the occurrence of cancer and exhibits advantageous benefits in individuals with cancer (Chen S. et al., 2014). Previous research has demonstrated that the consumption of fresh ginseng slices, juice, or tea has been associated with a reduced likelihood of developing many types of cancer, such as those affecting the pharynx, larynx, esophagus, stomach, colorectal, pancreatic, liver, lung, and ovarian regions (Lim et al., 2016). The presence of resveratrol in grape powder does not exhibit the ability to inhibit the Wnt pathway in colon cancer. However, it does demonstrate the capacity to inhibit the expression of Wnt target genes in the normal colonic mucosa of individuals diagnosed with colorectal cancer. This implies that resveratrol has advantageous properties in the onset and spread of colon cancer due to its modulation of Wnts and their downstream effectors, which play a crucial role in regulating various processes associated with tumor initiation, tumor growth, cell death, and metastasis (Jin et al., 2016). Furthermore, empirical investigations have demonstrated that flavonoids exhibit antiproliferative and apoptotic properties against diverse tumor cell lines, such as human lymphoma, breast cancer, osteosarcoma, and transformed hepatoma cells. A study including 250 urine samples obtained from Chinese women residing in Shanghai demonstrated a correlation between elevated excretion of total isoflavonoids and total lignans and a decreased likelihood of developing breast cancer (Situmorang PC. et al., 2024). Research has demonstrated that the incorporation of phytosterols into one’s diet can effectively mitigate the likelihood of developing obesity, diabetes, and cancer risk factors. The efficacy of phytosterols as anticancer agents has been demonstrated in several *in vivo* investigations, employing diverse cancer cell lines and animal models (Dai et al., 2002). Based on the above explanation, it is evident that numerous phytochemical constituents have been subjected to research and have progressed to the clinical trial phase. Herbal phytochemicals promote autophagy, a process by which cells undergo halting of aberrant growth and development. Autophagy is induced by the downregulation of the AKT and mTOR pathways in cancer cells (Ahmed et al., 2022). Hence, phytochemicals modulate antitumor effects in tissues via controlling inflammation, angiogenesis, invasion, and metastasis. The signaling pathways that phytochemicals use to halt carcinogenesis are illustrated in Figure 8.

## 11 Nanoformulation and green synthesis improve cancer-fighting natural chemicals

Various drug delivery technologies, such as synthetic polymers, microcapsules, and liposomes, possess the characteristic of enhancing medication bioavailability and promoting drug accumulation at the intended location. Herbal administration of anticancer medications is crucial since it minimizes or eliminates any adverse effect on healthy tissues. The utilization of nanoformulations is prevalent in the development of sunscreens, antibacterial medications, cholesterol biosensors, and dietary modulators for the purpose of managing diabetes and hyperlipidemia (Khafaga et al., 2023). Furthermore, it is imperative for the drug to possess functional groups that facilitate further alterations aimed at regulating drug release or binding to the target unit. The potential application of silver nanoparticle-based nanosystems as carriers for several therapeutic chemicals, including those possessing anti-inflammatory, antioxidant, antibacterial, and anticancer characteristics, has been the subject of investigation (Burdusel et al., 2018).

The present methodology for synthesizing nanoparticles in an environmentally sustainable manner is distinctive and time-efficient. Extensive research has been carried out on silver nanoparticles (AgNPs) due to their potential anticancer properties, as evidenced by multiple studies on human cancer. AgNPs exhibit significant potential as a highly efficient mechanism for delivering antitumor drugs. AgNPs address these limitations by mitigating adverse effects and enhancing the efficacy of cancer treatment. AgNPs have great potential as pharmaceuticals, as evidenced by the favorable outcomes of prior research and their affordability (Takáč et al., 2023). Several previously documented phytochemicals have demonstrated the potential of phytochemicals as cancer medicines because of their ability to form ionic or covalent connections with gold nanoparticles (AuNPs) and undergoing physical absorption. A hybrid system was formed by covalently linking polyacrylamide/dextran nano-hydrogel to silver nanoparticles, resulting in an *in vitro* release of 98.5%. Nevertheless, the utilization of AgNPs as therapeutic agents is hindered by certain inherent challenges pertaining to toxicity (Yusuf et al., 2023). In order to surmount these challenges and facilitate their application in preclinical trials involving people or other organisms, it is imperative that AgNPs exhibit biocompatibility, non-toxicity, and the absence of adverse effects. The selection of various mechanistic pathways, such as mitochondrial disruption, DNA fragmentation, cell membrane disruption, cellular signaling pathway disruption, enzyme activity changes, and reactive oxygen species (ROS), by AgNPs holds significant promise for therapeutic applications due to their ability to control the size, shape, and function of the corona surface. Utilizing phytochemicals found in plant extracts, AgNPs were produced by green synthesis for potential cancer treatment. Furthermore, this study also presents recent advancements and accomplishments in the application of silver nanoparticles (AgNPs) produced by green synthesis in the field of cancer therapy (Ratan et al., 2020). It also suggests probable mechanisms through which these nanoparticles may exhibit anticancer and cytotoxic properties. The comprehension of the molecular mechanisms behind the therapeutic effectiveness of AgNPs holds promise for the development of targeted therapies and treatments for cancer, hence presenting a promising avenue for cancer treatment (Wahi et al., 2023).

## 12 Conclusion

Cancer is characterized by abnormal cell metabolism, which is considered a biological indicator. The preferred approach for cancer treatment is the use of plant compounds for chemoprevention. The observed preventive and therapeutic advantages of these bioactive chemicals against different types of cancer can be attributed to their ability to modulate signal transduction pathways. The consideration of compounds possessing antioxidant properties as a preventive intervention against cancer is warranted due to the substantial role of oxidative stress in the pathogenesis of diverse malignant illnesses. Seven primary bioactive chemicals, namely, alkaloids, tannins, flavonoids, phenols, steroids, terpenoids, and saponins, found in plants have anticancer activities. These compounds play a significant role in modulating cell growth. The process of angiogenesis and metastasis inhibition in cancer is mediated by various mechanisms, including the MAPK/ERK pathway, TNF signaling, death receptors, p53, p38, and actin dynamics. In the context of combating cancer resistance, novel molecular processes necessitate the exploration of novel multitargets. Phytochemical substances, whether transformed into natural small molecules or nanostructured formulations, exhibit considerable potential as multitarget agents due to their reduced side effects and enhanced efficacy. This phenomenon not only exerts an impact on the proliferation of malignant cells but also offers practical insights for the investigation of therapeutic agents that specifically target tumors, hence paving the way for novel avenues in the realm of cancer prevention and therapy.

## References

[B1] Abbaspour Babaei M. Zaman Huri H. Kamalidehghan B. Yeap S. K. Ahmadipour F. (2017). Apoptotic induction and inhibition of NF-κB signaling pathway in human prostatic cancer PC3 cells by natural compound 2,2’-oxybis (4-allyl-1-methoxybenzene), biseugenol B, from Litsea costalis: an *in vitro* study. OncoTargets Ther. 10, 277–294. 10.2147/OTT.S102894 PMC5237594 28138251

[B2] Afzal S. Abdul Manap A. S. Attiq A. Albokhadaim I. Kandeel M. Alhojaily S. M. (2023). From imbalance to impairment: the central role of reactive oxygen species in oxidative stress-induced disorders and therapeutic exploration. Front. Pharmacol. 14, 1269581. 10.3389/fphar.2023.1269581 37927596 PMC10622810

[B3] Aghajanpour M. Nazer M. R. Obeidavi Z. Akbari M. Ezati P. Kor N. M. (2017). Functional foods and their role in cancer prevention and health promotion: a comprehensive review. Am. J. cancer Res. 7, 740–769. 28469951 PMC5411786

[B4] Agrotis A. von Chamier L. Oliver H. Kiso K. Singh T. Ketteler R. (2019). Human ATG4 autophagy proteases counteract attachment of ubiquitin-like LC3/GABARAP proteins to other cellular proteins. J. Biol. Chem. 294, 12610–12621. 10.1074/jbc.AC119.009977 31315929 PMC6709618

[B5] Ahmed M. B. Islam S. U. Alghamdi A. A. A. Kamran M. Ahsan H. Lee Y. S. (2022). Phytochemicals as chemo-preventive agents and signaling molecule modulators: current role in cancer therapeutics and inflammation. Int. J. Mol. Sci. 23 (24), 15765. 10.3390/ijms232415765 36555406 PMC9779495

[B6] Akhtar N. Baig M. W. Haq I.-U. Rajeeve V. Cutillas P. R. (2020). Withanolide metabolites inhibit PI3K/AKT and MAPK pro-survival pathways and induce apoptosis in acute myeloid leukemia cells. Biomedicines 8, 333. 10.3390/biomedicines8090333 32899914 PMC7555989

[B7] Albert-Gascó H. Ros-Bernal F. Castillo-Gómez E. Olucha-Bordonau F. E. (2020). MAP/ERK signaling in developing cognitive and emotional function and its effect on pathological and neurodegenerative processes. Int. J. Mol. Sci. 21, 4471. 10.3390/ijms21124471 32586047 PMC7352860

[B8] Almatroodi S. A. Almatroudi A. Alsahli M. A. Khan A. A. Rahmani A. H. (2020). Thymoquinone, an active compound of nigella sativa: role in prevention and treatment of cancer. Curr. Pharm. Biotechnol. 21, 1028–1041. 10.2174/1389201021666200416092743 32297580

[B9] Almatroodi S. A. Almatroudi A. Khan A. A. Rahmani A. H. (2023). Potential therapeutic targets of formononetin, a type of methoxylated isoflavone, and its role in cancer therapy through the modulation of signal transduction pathways. Int. J. Mol. Sci. 24, 9719. 10.3390/ijms24119719 37298670 PMC10253309

[B10] Asgharian P. Tazekand A. P. Hosseini K. Forouhandeh H. Ghasemnejad T. Ranjbar M. (2022). Potential mechanisms of quercetin in cancer prevention: focus on cellular and molecular targets. Cancer Cell Int. 22, 257. 10.1186/s12935-022-02677-w 35971151 PMC9380290

[B11] Ashikawa K. Majumdar S. Banerjee S. Bharti A. C. Shishodia S. Aggarwal B. B. (2002). Piceatannol inhibits TNF-induced NF-kappaB activation and NF-kappaB-mediated gene expression through suppression of IkappaBalpha kinase and p65 phosphorylation. J. Immunol. 169, 6490–6497. 10.4049/jimmunol.169.11.6490 12444159

[B12] Asih P. R. Prikas E. Stefanoska K. Tan A. R. P. Ahel H. I. Ittner A. (2020). Functions of p38 MAP kinases in the central nervous system. Front. Mol. Neurosci. 13, 570586. 10.3389/fnmol.2020.570586 33013322 PMC7509416

[B13] Azios N. G. Krishnamoorthy L. Harris M. Cubano L. A. Cammer M. Dharmawardhane S. F. (2007). Estrogen and resveratrol regulate Rac and Cdc42 signaling to the actin cytoskeleton of metastatic breast cancer cells. Neoplasia (New York, N.Y.) 9, 147–158. 10.1593/neo.06778 17356711 PMC1813930

[B14] Bai Y. Chen J. Hu W. Wang L. Wu Y. Yu S. (2022). Silibinin therapy improves cholangiocarcinoma outcomes by regulating ERK/mitochondrial pathway. Front. Pharmacol. 13, 847905. 10.3389/fphar.2022.847905 35401195 PMC8983842

[B15] Bakrim S. El Omari N. El Hachlafi N. Bakri Y. Lee L.-H. Bouyahya A. (2022). Dietary phenolic compounds as anticancer natural drugs: recent update on molecular mechanisms and clinical trials. Foods 11, 3323. 10.3390/foods11213323 36359936 PMC9657352

[B16] Balaji C. Muthukumaran J. Nalini N. (2014). Chemopreventive effect of sinapic acid on 1,2-dimethylhydrazine-induced experimental rat colon carcinogenesis. Hum. Exp. Toxicol. 33, 1253–1268. 10.1177/0960327114522501 24532707

[B17] Balakrishna P. George S. Hatoum H. Mukherjee S. (2021). Serotonin pathway in cancer. Int. J. Mol. Sci. 22, 1268. 10.3390/ijms22031268 33525332 PMC7865972

[B18] Bhat S. S. Prasad S. K. Shivamallu C. Prasad K. S. Syed A. Reddy P. (2021). Genistein: a potent anti-breast cancer agent. Curr. issues Mol. Biol. 43, 1502–1517. 10.3390/cimb43030106 34698063 PMC8929066

[B19] Bispo J. A. B. Pinheiro P. S. Kobetz E. K. (2020). Epidemiology and etiology of leukemia and lymphoma. Cold Spring Harb. Perspect. Med. 10, a034819. 10.1101/cshperspect.a034819 31727680 PMC7263093

[B20] Biswas P. Dey D. Biswas P. K. Rahaman T. I. Saha S. Parvez A. (2022). A comprehensive analysis and anti-cancer activities of quercetin in ROS-mediated cancer and cancer stem cells. Int. J. Mol. Sci. 23, 11746. 10.3390/ijms231911746 36233051 PMC9569933

[B21] Borges G. A. Elias S. T. Amorim B. de Lima C. L. Coletta R. D. Castilho R. M. (2020). Curcumin downregulates the PI3K-AKT-mTOR pathway and inhibits growth and progression in head and neck cancer cells. Phytotherapy Res. 34, 3311–3324. 10.1002/ptr.6780 32628350

[B22] Brouckaert G. Kalai M. Krysko D. V. Saelens X. Vercammen D. Ndlovu M. N. (2004). Phagocytosis of necrotic cells by macrophages is phosphatidylserine dependent and does not induce inflammatory cytokine production. Mol. Biol. Cell 15, 1089–1100. 10.1091/mbc.e03-09-0668 14668480 PMC363082

[B23] Brown J. S. Amend S. R. Austin R. H. Gatenby R. A. Hammarlund E. U. Pienta K. J. (2023). Updating the definition of cancer. Mol. cancer Res. MCR 21, 1142–1147. 10.1158/1541-7786.MCR-23-0411 37409952 PMC10618731

[B24] Buhrmann C. Yazdi M. Popper B. Shayan P. Goel A. Aggarwal B. B. (2019). Evidence that TNF-β induces proliferation in colorectal cancer cells and resveratrol can down-modulate it. Exp. Biol. Med. (Maywood, N.J.) 244, 1–12. 10.1177/1535370218824538 PMC6362535 30661394

[B25] Burdusel A. C. Gherasim O. Grumezescu A. Mogoantă L. Ficai A. Andronescu E. (2018). Biomedical applications of silver nanoparticles: an up-to-date overview. Nanomaterials 8, 681. 10.3390/nano8090681 30200373 PMC6163202

[B26] Cargnello M. Roux P. P. (2011). Activation and function of the MAPKs and their substrates, the MAPK-activated protein kinases. Microbiol. Mol. Biol. Rev. 75, 50–83. 10.1128/MMBR.00031-10 21372320 PMC3063353

[B27] Chan L.-P. Chou T.-H. Ding H.-Y. Chen P.-R. Chiang F.-Y. Kuo P.-L. (2012). Apigenin induces apoptosis via tumor necrosis factor receptor- and Bcl-2-mediated pathway and enhances susceptibility of head and neck squamous cell carcinoma to 5-fluorouracil and cisplatin. Biochimica biophysica acta 1820, 1081–1091. 10.1016/j.bbagen.2012.04.013 22554915

[B28] Chang C.-I. Chien W.-C. Huang K.-X. Hsu J.-L. (2017). Anti-inflammatory effects of vitisinol A and four other oligostilbenes from ampelopsis brevipedunculata var. Hancei. Molecules 22, 1195. 10.3390/molecules22071195 28714918 PMC6152071

[B29] Chang Y. J. Hsu S. L. Liu Y. T. Lin Y. H. Lin M. H. Huang S. J. (2015). Gallic acid induces necroptosis via TNF-α signaling pathway in activated hepatic stellate cells. PloS one 10, e0120713. 10.1371/journal.pone.0120713 25816210 PMC4376672

[B30] Chao J.-I. Su W.-C. Liu H.-F. (2007). Baicalein induces cancer cell death and proliferation retardation by the inhibition of CDC2 kinase and survivin associated with opposite role of p38 mitogen-activated protein kinase and AKT. Mol. cancer Ther. 6, 3039–3048. 10.1158/1535-7163.MCT-07-0281 18025287

[B31] Chebet J. J. Ehiri J. E. McClelland D. J. Taren D. Hakim I. A. (2021). Effect of d-limonene and its derivatives on breast cancer in human trials: a scoping review and narrative synthesis. BMC cancer 21, 902. 10.1186/s12885-021-08639-1 34362338 PMC8349000

[B32] Chen J. (2016). The cell-cycle arrest and apoptotic functions of p53 in tumor initiation and progression. Cold Spring Harb. Perspect. Med. 6, a026104. 10.1101/cshperspect.a026104 26931810 PMC4772082

[B33] Chen J. Ye C. Wan C. Li G. Peng L. Peng Y. (2021). The roles of c-jun N-terminal kinase (JNK) in infectious diseases. Int. J. Mol. Sci. 22, 9640. 10.3390/ijms22179640 34502556 PMC8431791

[B34] Chen J.-C. Wu C.-H. Peng Y.-S. Zheng H.-Y. Lin Y.-C. Ma P.-F. (2018). Astaxanthin enhances erlotinib-induced cytotoxicity by p38 MAPK mediated xeroderma pigmentosum complementation group C (XPC) down-regulation in human lung cancer cells. Toxicol. Res. 7, 1247–1256. 10.1039/c7tx00292k PMC6247823 30555679

[B35] Chen K. Zhang S. Ji Y. Li J. An P. Ren H. (2013b). Baicalein inhibits the invasion and metastatic capabilities of hepatocellular carcinoma cells via down-regulation of the ERK pathway. PloS one 8, e72927. 10.1371/journal.pone.0072927 24039823 PMC3765161

[B36] Chen K.-C. Chen C.-Y. Lin C.-R. Yang T.-Y. Chen T.-H. Wu L.-C. (2013a). Luteolin attenuates TGF-β1-induced epithelial-mesenchymal transition of lung cancer cells by interfering in the PI3K/Akt-NF-κB-Snail pathway. Life Sci. 93, 924–933. 10.1016/j.lfs.2013.10.004 24140887

[B37] Chen Q. Zheng Y. Jiao D. Chen F. Hu H. Wu Y. (2014a). Curcumin inhibits lung cancer cell migration and invasion through Rac1-dependent signaling pathway. J. Nutr. Biochem. 25, 177–185. 10.1016/j.jnutbio.2013.10.004 24445042

[B38] Chen S. Wang Z. Huang Y. O’Barr S. A. Wong R. A. Yeung S. (2014b). Ginseng and anticancer drug combination to improve cancer chemotherapy: a critical review. Evidence-based complementary Altern. Med. 2014, 168940. 10.1155/2014/168940 PMC4021740 24876866

[B39] Cheng A.-W. Tan X. Sun J.-Y. Gu C.-M. Liu C. Guo X. (2019). Catechin attenuates TNF-α induced inflammatory response via AMPK-SIRT1 pathway in 3T3-L1 adipocytes. PloS one 14, e0217090. 10.1371/journal.pone.0217090 31100089 PMC6524818

[B40] Cheng L. Xia F. Li Z. Shen C. Yang Z. Hou H. (2023). Structure, function and drug discovery of GPCR signaling. Mol. Biomed. 4, 46. 10.1186/s43556-023-00156-w 38047990 PMC10695916

[B41] Cheon C. Ko S.-G. (2022). Synergistic effects of natural products in combination with anticancer agents in prostate cancer: a scoping review. Front. Pharmacol. 13, 963317. 10.3389/fphar.2022.963317 36172195 PMC9510769

[B42] Choudhari A. S. Mandave P. C. Deshpande M. Ranjekar P. Prakash O. (2019). Phytochemicals in cancer treatment: from preclinical studies to clinical practice. Front. Pharmacol. 10, 1614. 10.3389/fphar.2019.01614 32116665 PMC7025531

[B43] Chung K.-S. Choi J.-H. Back N.-I. Choi M.-S. Kang E.-K. Chung H.-G. (2010). Eupafolin, a flavonoid isolated from Artemisia princeps, induced apoptosis in human cervical adenocarcinoma HeLa cells. Mol. Nutr. food Res. 54, 1318–1328. 10.1002/mnfr.200900305 20397191

[B44] Collins H. M. Abdelghany M. K. Messmer M. Yue B. Deeves S. E. Kindle K. B. (2013). Differential effects of garcinol and curcumin on histone and p53 modifications in tumour cells. BMC cancer 13, 37. 10.1186/1471-2407-13-37 23356739 PMC3583671

[B45] Cruz M. A. A. S. Coimbra P. P. S. Araújo-Lima C. F. Freitas-Silva O. Teodoro A. J. (2024). Hybrid fruits for improving health-A comprehensive review. Foods 13, 219. 10.3390/foods13020219 38254523 PMC10814314

[B46] Cui J. Li X. Wang S. Su Y. Chen X. Cao L. (2020). Triptolide prevents bone loss via suppressing osteoclastogenesis through inhibiting PI3K-AKT-NFATc1 pathway. J. Cell. Mol. Med. 24, 6149–6161. 10.1111/jcmm.15229 32347017 PMC7294126

[B47] Dai Q. Franke A. A. Jin F. Shu X.-O. Hebert J. R. Custer L. J. (2002). Urinary excretion of phytoestrogens and risk of breast cancer among Chinese women in Shanghai. Cancer Epidemiol. biomarkers Prev. 11, 815–821. 12223424

[B48] Dembitsky V. M. Gloriozova T. A. Poroikov V. V. (2021). Antitumor profile of carbon-bridged steroids (CBS) and triterpenoids. Mar. drugs 19, 324. 10.3390/md19060324 34205074 PMC8228860

[B49] Dhokia V. Moss J. A. Y. Macip S. Fox J. L. (2022). At the crossroads of life and death: the proteins that influence cell fate decisions. Cancers 14, 2745. 10.3390/cancers14112745 35681725 PMC9179324

[B50] Dhyani P. Quispe C. Sharma E. Bahukhandi A. Sati P. Attri D. C. (2022). Anticancer potential of alkaloids: a key emphasis to colchicine, vinblastine, vincristine, vindesine, vinorelbine and vincamine. Cancer Cell Int. 22, 206. 10.1186/s12935-022-02624-9 35655306 PMC9161525

[B51] Djavan B. Nasu Y. (2001). Prostate cancer gene therapy-what have we learned and where are we going? Rev. urology 3, 179–186. PMC1476064 16985716

[B52] Dos Santos M. B. Bertholin Anselmo D. de Oliveira J. G. Jardim-Perassi B. V. Alves Monteiro D. Silva G. (2019). Antiproliferative activity and p53 upregulation effects of chalcones on human breast cancer cells. J. enzyme inhibition Med. Chem. 34, 1093–1099. 10.1080/14756366.2019.1615485 PMC6534249 31117836

[B53] Dowling J. J. Weihl C. C. Spencer M. J. (2021). Molecular and cellular basis of genetically inherited skeletal muscle disorders. Nat. Rev. Mol. Cell Biol. 22, 713–732. 10.1038/s41580-021-00389-z 34257452 PMC9686310

[B54] Duan W.-J. Li Q.-S. Xia M.-Y. Tashiro S.-I. Onodera S. Ikejima T. (2011). Silibinin activated ROS-p38-NF-κB positive feedback and induced autophagic death in human fibrosarcoma HT1080 cells. J. Asian Nat. Prod. Res. 13, 27–35. 10.1080/10286020.2010.540757 21253947

[B55] Efferth T. Saeed M. E. M. Mirghani E. Alim A. Yassin Z. Saeed E. (2017). Integration of phytochemicals and phytotherapy into cancer precision medicine. Oncotarget 8, 50284–50304. 10.18632/oncotarget.17466 28514737 PMC5564849

[B56] Elekofehinti O. O. Iwaloye O. Olawale F. Ariyo E. O. (2021). Saponins in cancer treatment: current progress and future prospects. Pathophysiol. official J. Int. Soc. Pathophysiol. 28, 250–272. 10.3390/pathophysiology28020017 PMC8830467 35366261

[B57] Elmore S. (2007). Apoptosis: a review of programmed cell death. Toxicol. Pathol. 35, 495–516. 10.1080/01926230701320337 17562483 PMC2117903

[B58] Evangelatos G. Bamias G. Kitas G. D. Kollias G. Sfikakis P. P. (2022). The second decade of anti-TNF-a therapy in clinical practice: new lessons and future directions in the COVID-19 era. Rheumatol. Int. 42, 1493–1511. 10.1007/s00296-022-05136-x 35503130 PMC9063259

[B59] Falcicchia C. Tozzi F. Arancio O. Watterson D. M. Origlia N. (2020). Involvement of p38 MAPK in synaptic function and dysfunction. Int. J. Mol. Sci. 21, 5624. 10.3390/ijms21165624 32781522 PMC7460549

[B60] Fawaz A. Ferraresi A. Isidoro C. (2023). Systems biology in cancer diagnosis integrating omics technologies and artificial intelligence to support physician decision making. J. personalized Med. 13, 1590. 10.3390/jpm13111590 PMC10672164 38003905

[B61] Fu H. Wang C. Yang D. Wei Z. Xu J. Hu Z. (2018). Curcumin regulates proliferation, autophagy, and apoptosis in gastric cancer cells by affecting PI3K and P53 signaling. J. Cell. physiology 233, 4634–4642. 10.1002/jcp.26190 28926094

[B62] G M. S. Swetha M. Keerthana C. K. Rayginia T. P. Anto R. J. (2021). Cancer chemoprevention: a strategic approach using phytochemicals. Front. Pharmacol. 12, 809308. 10.3389/fphar.2021.809308 35095521 PMC8793885

[B63] Gan D. He W. Yin H. Gou X. (2020). β-elemene enhances cisplatin-induced apoptosis in bladder cancer cells through the ROS-AMPK signaling pathway. Oncol. Lett. 19, 291–300. 10.3892/ol.2019.11103 31897141 PMC6924103

[B64] Ganguly P. Macleod T. Wong C. Harland M. McGonagle D. (2023). Revisiting p38 mitogen-activated protein kinases (MAPK) in inflammatory arthritis: a narrative of the emergence of MAPK-activated protein kinase inhibitors (MK2i). Pharm. (Basel) 16, 1286. 10.3390/ph16091286 PMC10537904 37765094

[B65] Gao Y. Yin J. Rankin G. O. Chen Y. C. (2018). Kaempferol induces G2/M cell cycle arrest via checkpoint kinase 2 and promotes apoptosis via death receptors in human ovarian carcinoma A2780/CP70 cells. Molecules 23, 1095. 10.3390/molecules23051095 29734760 PMC6065264

[B66] George B. P. Chandran R. Abrahamse H. (2021). Role of phytochemicals in cancer chemoprevention: insights. Antioxidants (Basel) 10, 1455. 10.3390/antiox10091455 34573087 PMC8466984

[B67] Gnesutta N. Minden A. (2003). Death receptor-induced activation of initiator caspase 8 is antagonized by serine/threonine kinase PAK4. Mol. Cell. Biol. 23, 7838–7848. 10.1128/MCB.23.21.7838-7848.2003 14560027 PMC207651

[B68] Gómez-Virgilio L. Silva-Lucero M.-D.-C. Flores-Morelos D.-S. Gallardo-Nieto J. Lopez-Toledo G. Abarca-Fernandez A.-M. (2022). Autophagy: a key regulator of homeostasis and disease: an overview of molecular mechanisms and modulators. Cells 11, 2262. 10.3390/cells11152262 35892559 PMC9329718

[B69] Granado-Serrano A. B. Martín M. Á. Bravo L. Goya L. Ramos S. (2012). Quercetin attenuates TNF-induced inflammation in hepatic cells by inhibiting the NF-κB pathway. Nutr. cancer 64, 588–598. 10.1080/01635581.2012.661513 22452660

[B70] Green D. R. (2022). The death receptor pathway of apoptosis. Cold Spring Harb. Perspect. Biol. 14, a041053. 10.1101/cshperspect.a041053 35105716 PMC8805650

[B71] Guillamón E. Mut-Salud N. Rodríguez-Sojo M. J. Ruiz-Malagón A. J. Cuberos-Escobar A. Martínez-Férez A. (2023). *In vitro* antitumor and anti-inflammatory activities of allium-derived compounds propyl propane thiosulfonate (PTSO) and propyl propane thiosulfinate (PTS). Nutrients 15, 1363. 10.3390/nu15061363 36986093 PMC10058678

[B72] Gulati N. Laudet B. Zohrabian V. M. Murali R. Jhanwar-Uniyal M. (2006). The antiproliferative effect of Quercetin in cancer cells is mediated via inhibition of the PI3K-Akt/PKB pathway. Anticancer Res. 26, 1177–1181. 16619521

[B73] Guo Y.-B. Bao X.-J. Xu S.-B. Zhang X.-D. Liu H.-Y. (2015). Honokiol induces cell cycle arrest and apoptosis via p53 activation in H4 human neuroglioma cells. Int. J. Clin. Exp. Med. 8, 7168–7175. 26221255 PMC4509200

[B74] Gupta S. C. Patchva S. Koh W. Aggarwal B. B. (2012). Discovery of curcumin, a component of golden spice, and its miraculous biological activities. Clin. Exp. Pharmacol. physiology 39, 283–299. 10.1111/j.1440-1681.2011.05648.x PMC3288651 22118895

[B75] Gv V. Ranganathan P. J. Palati S. (2023). Tangeretin’s anti-apoptotic signaling mechanisms in oral cancer cells: *in vitro* anti-cancer activity. Cureus 15, e47452. 10.7759/cureus.47452 38022093 PMC10660419

[B76] Hafeez B. B. Mustafa A. Fischer J. W. Singh A. Zhong W. Shekhani M. O. (2014). α-Mangostin: a dietary antioxidant derived from the pericarp of Garcinia mangostana L. inhibits pancreatic tumor growth in xenograft mouse model. Antioxidants redox Signal. 21, 682–699. 10.1089/ars.2013.5212 PMC4104617 24295217

[B77] Hehlgans T. Pfeffer K. (2005). The intriguing biology of the tumour necrosis factor/tumour necrosis factor receptor superfamily: players, rules and the games. Immunology 115, 1–20. 10.1111/j.1365-2567.2005.02143.x 15819693 PMC1782125

[B78] Ho J. F. V. Yaakup H. Low G. S. H. Wong S. L. Tho L. M. Tan S. B. (2020). Morphine use for cancer pain: a strong analgesic used only at the end of life? A qualitative study on attitudes and perceptions of morphine in patients with advanced cancer and their caregivers. Palliat. Med. 34, 619–629. 10.1177/0269216320904905 32103707 PMC7238510

[B79] Hoffmann J. C. Pappa A. Krammer P. H. Lavrik I. N. (2009). A new C-terminal cleavage product of procaspase-8, p30, defines an alternative pathway of procaspase-8 activation. Mol. Cell. Biol. 29, 4431–4440. 10.1128/MCB.02261-07 19528225 PMC2725745

[B80] Hong T.-K. Song J.-H. Lee S.-B. Do J.-T. (2021). Germ cell derivation from pluripotent stem cells for understanding *in vitro* gametogenesis. Cells 10, 1889. 10.3390/cells10081889 34440657 PMC8394365

[B81] Hu C. He Y. (2021). Formononetin inhibits non-small cell lung cancer proliferation via regulation of mir-27a-3p through p53 pathway. Oncologie 23, 241–250. 10.32604/Oncologie.2021.015828

[B82] Hu Y. Reggiori F. (2022). Molecular regulation of autophagosome formation. Biochem. Soc. Trans. 50, 55–69. 10.1042/BST20210819 35076688 PMC9022990

[B83] Huang Y. Wang G. Zhang N. Zeng X. (2024). MAP3K4 kinase action and dual role in cancer. Discov. Oncol. 15, 99. 10.1007/s12672-024-00961-x 38568424 PMC10992237

[B84] Hun Lee J. Shu L. Fuentes F. Su Z.-Y. Tony Kong A.-N. (2013). Cancer chemoprevention by traditional Chinese herbal medicine and dietary phytochemicals: targeting nrf2-mediated oxidative stress/anti-inflammatory responses, epigenetics, and cancer stem cells. J. traditional complementary Med. 3, 69–79. 10.4103/2225-4110.107700 PMC3924975 24716158

[B85] Hussain Y. Cui J. H. Khan H. Aschner M. Batiha G. E.-S. Jeandet P. (2021). Luteolin and cancer metastasis suppression: focus on the role of epithelial to mesenchymal transition. Med. Oncol. N. Lond. Engl. 38, 66. 10.1007/s12032-021-01508-8 33950369

[B86] Ilyas S. Simanullang R. H. Hutahaean S. Rosidah R. Situmorang P. C. (2021). Effect of *Zanthoxylum acanthopodium* methanol extract on CDK4 expression to cervical cancer. Res. J Pharm. Technol. 14 (11), 5647–5652. 10.52711/0974-360X.2021.00982

[B87] Ilyas S. Simanullang R. H. Hutahaean S. Rosidah R. Situmorang P. C. (2022). Correlation of myc expression with Wee1 expression by *Zanthoxylum acanthopodium* in cervical carcinoma histology. Pak J. Biol. Sci. 25 (11), 1014–1020. 10.3923/pjbs.2022.1014.1020 36591933

[B88] Imai-Sumida M. Chiyomaru T. Majid S. Saini S. Nip H. Dahiya R. (2017). Silibinin suppresses bladder cancer through down-regulation of actin cytoskeleton and PI3K/Akt signaling pathways. Oncotarget 8, 92032–92042. 10.18632/oncotarget.20734 29190895 PMC5696161

[B89] Irianti E. Ilyas S. Hutahaean S. Rosidah Situmorang P. C. (2020). Placental histological on preeclamptic rats *(Rattus norvegicus*) after administration of nanoherbal haramonting (*Rhodomyrtus tomentosa)* . Res. J. Pharm. Tech. 13 (8), 3879–3882. 10.5958/0974-360X.2020.00686.1

[B90] Jang J. H. Yang E. S. Min K.-J. Kwon T. K. (2012). Inhibitory effect of butein on tumor necrosis factor-α-induced expression of cell adhesion molecules in human lung epithelial cells via inhibition of reactive oxygen species generation, NF-κB activation and Akt phosphorylation. Int. J. Mol. Med. 30, 1357–1364. 10.3892/ijmm.2012.1158 23064245

[B91] Jang J. Y. Im E. Kim N. D. (2022). Mechanism of resveratrol-induced programmed cell death and new drug discovery against cancer: a review. Int. J. Mol. Sci. 23, 13689. 10.3390/ijms232213689 36430164 PMC9697740

[B92] Jeong J.-H. Nakajima H. Magae J. Furukawa C. Taki K. Otsuka K. (2009). Ascochlorin activates p53 in a manner distinct from DNA damaging agents. Int. J. cancer 124, 2797–2803. 10.1002/ijc.24259 19253369

[B93] Jia S. Xu X. Zhou S. Chen Y. Ding G. Cao L. (2019). Fisetin induces autophagy in pancreatic cancer cells via endoplasmic reticulum stress- and mitochondrial stress-dependent pathways. Cell death Dis. 10, 142. 10.1038/s41419-019-1366-y 30760707 PMC6374379

[B94] Jin X. Che D.-B. Zhang Z.-H. Yan H.-M. Jia Z.-Y. Jia X.-B. (2016). Ginseng consumption and risk of cancer: a meta-analysis. J. ginseng Res. 40, 269–277. 10.1016/j.jgr.2015.08.007 27616903 PMC5005362

[B95] Jo J. Abdi Nansa S. Kim D.-H. (2020). Molecular regulators of cellular mechanoadaptation at cell-material interfaces. Front. Bioeng. Biotechnol. 8, 608569. 10.3389/fbioe.2020.608569 33364232 PMC7753015

[B96] Johnson R. Dludla P. V. Muller C. J. F. Huisamen B. Essop M. F. Louw J. (2017). The transcription profile unveils the cardioprotective effect of aspalathin against lipid toxicity in an *in vitro* H9c2 model. Molecules 22, 219. 10.3390/molecules22020219 28146135 PMC6155936

[B97] Jung J. E. Kim H. S. Lee C. S. Park D.-H. Kim Y.-N. Lee M.-J. (2007). Caffeic acid and its synthetic derivative CADPE suppress tumor angiogenesis by blocking STAT3-mediated VEGF expression in human renal carcinoma cells. Carcinogenesis 28, 1780–1787. 10.1093/carcin/bgm130 17557905

[B98] Kang S. H. Bak D.-H. Chung B. Y. Bai H.-W. Kang B. S. (2020). Delphinidin enhances radio-therapeutic effects via autophagy induction and JNK/MAPK pathway activation in non-small cell lung cancer. Korean J. physiology Pharmacol. 24, 413–422. 10.4196/kjpp.2020.24.5.413 PMC7445475 32830148

[B99] Kanwal N. Rasul A. Hussain G. Anwar H. Shah M. A. Sarfraz I. (2020). Oleandrin: a bioactive phytochemical and potential cancer killer via multiple cellular signaling pathways. Food Chem. Toxicol. 143, 111570. 10.1016/j.fct.2020.111570 32640345

[B100] Kashafi E. Moradzadeh M. Mohamadkhani A. Erfanian S. (2017). Kaempferol increases apoptosis in human cervical cancer HeLa cells via PI3K/AKT and telomerase pathways. Biomed. Pharmacother. 89, 573–577. 10.1016/j.biopha.2017.02.061 28258039

[B101] Kast R. E. (2003). Ribavirin in cancer immunotherapies: controlling nitric oxide augments cytotoxic lymphocyte function. Neoplasia (New York, N.Y.) 5, 3–8. 10.1016/s1476-5586(03)80011-8 12659664 PMC1502126

[B102] Kavitha K. Kowshik J. Kishore T. K. K. Baba A. B. Nagini S. (2013). Astaxanthin inhibits NF-κB and Wnt/β-catenin signaling pathways via inactivation of Erk/MAPK and PI3K/Akt to induce intrinsic apoptosis in a hamster model of oral cancer. Biochimica biophysica acta 1830, 4433–4444. 10.1016/j.bbagen.2013.05.032 23726989

[B103] Kciuk M. Alam M. Ali N. Rashid S. Głowacka P. Sundaraj R. (2023). Epigallocatechin-3-Gallate therapeutic potential in cancer: mechanism of action and clinical implications. Molecules 28, 5246. 10.3390/molecules28135246 37446908 PMC10343677

[B104] Kciuk M. Gielecińska A. Budzinska A. Mojzych M. Kontek R. (2022). Metastasis and MAPK pathways. Int. J. Mol. Sci. 23, 3847. 10.3390/ijms23073847 35409206 PMC8998814

[B105] Khafaga D. S. R. El-Khawaga A. M. Elfattah Mohammed R. A. Abdelhakim H. K. (2023). Green synthesis of nano-based drug delivery systems developed for hepatocellular carcinoma treatment: a review. Mol. Biol. Rep. 50, 10351–10364. 10.1007/s11033-023-08823-5 37817020 PMC10676320

[B106] Kikuchi H. Yuan B. Yuhara E. Takagi N. Toyoda H. (2013). Involvement of histone H3 phosphorylation through p38 MAPK pathway activation in casticin-induced cytocidal effects against the human promyelocytic cell line HL-60. Int. J. Oncol. 43, 2046–2056. 10.3892/ijo.2013.2106 24064676

[B107] Kim H. Lee M.-J. Kim J.-E. Park S.-D. Moon H.-I. Park W.-H. (2010). Genistein suppresses tumor necrosis factor-alpha-induced proliferation via the apoptotic signaling pathway in human aortic smooth muscle cells. J. Agric. food Chem. 58, 2015–2019. 10.1021/jf903802v 20067268

[B108] Kim H.-J. Hwang K.-E. Park D.-S. Oh S.-H. Jun H. Y. Yoon K.-H. (2017). Shikonin-induced necroptosis is enhanced by the inhibition of autophagy in non-small cell lung cancer cells. J. Transl. Med. 15, 123. 10.1186/s12967-017-1223-7 28569199 PMC5452303

[B109] Kim M. E. Na J. Y. Lee J. S. (2018). Anti-inflammatory effects of trans-cinnamaldehyde on lipopolysaccharide-stimulated macrophage activation via MAPKs pathway regulation. Immunopharmacol. Immunotoxicol. 40, 219–224. 10.1080/08923973.2018.1424902 29355056

[B110] Kim M. S. Kang H. J. Moon A. (2001). Inhibition of invasion and induction of apoptosis by curcumin in H-ras-transformed MCF10A human breast epithelial cells. Archives pharmacal Res. 24, 349–354. 10.1007/BF02975105 11534770

[B111] Kim N. Chung G. Son S.-R. Park J. H. Lee Y. H. Park K.-T. (2023). Magnolin inhibits paclitaxel-induced cold allodynia and ERK1/2 activation in mice. Plants 12, 2283. 10.3390/plants12122283 37375908 PMC10303305

[B112] King S. J. Worth D. C. Scales T. M. E. Monypenny J. Jones G. E. Parsons M. (2011). β1 integrins regulate fibroblast chemotaxis through control of N-WASP stability. EMBO J. 30, 1705–1718. 10.1038/emboj.2011.82 21427700 PMC3101992

[B113] Kłósek M. Mertas A. Król W. Jaworska D. Szymszal J. Szliszka E. (2016). Tumor necrosis factor-related apoptosis-inducing ligand-induced apoptosis in prostate cancer cells after treatment with xanthohumol-A natural compound present in humulus lupulus L. Int. J. Mol. Sci. 17, 837. 10.3390/ijms17060837 27338375 PMC4926371

[B114] Ko A. M.-S. Tu H.-P. Ko Y.-C. (2023). Systematic review of roles of arecoline and arecoline N-oxide in oral cancer and strategies to block carcinogenesis. Cells 12, 1208. 10.3390/cells12081208 37190117 PMC10137008

[B115] Kou Y. Tong B. Wu W. Liao X. Zhao M. (2020). Berberine improves chemo-sensitivity to cisplatin by enhancing cell apoptosis and repressing PI3K/AKT/mTOR signaling pathway in gastric cancer. Front. Pharmacol. 11, 616251. 10.3389/fphar.2020.616251 33362566 PMC7756080

[B116] Koul H. K. Pal M. Koul S. (2013). Role of p38 MAP kinase signal transduction in solid tumors. Genes & cancer 4, 342–359. 10.1177/1947601913507951 24349632 PMC3863344

[B117] Kretschmer N. Rinner B. Stuendl N. Kaltenegger H. Wolf E. Kunert O. (2012). Effect of costunolide and dehydrocostus lactone on cell cycle, apoptosis, and ABC transporter expression in human soft tissue sarcoma cells. Planta medica. 78, 1749–1756. 10.1055/s-0032-1315385 23047249

[B118] Kurniasih N. Supriadin A. Harneti D. Abdulah R. Azmi M. Supratman U. (2021). Ergosterol peroxide and stigmasterol from the stembark of aglaia simplicifolia (meliaceae) and their cytotoxic against HeLa cervical cancer cell lines. J. Kim. Val. 1, 46–51. 10.15408/jkv.v1i1.20068

[B119] Kwon O. S. Jung J. H. Shin E. A. Park J. E. Park W. Y. Kim S.-H. (2020). Epigallocatechin-3-Gallate induces apoptosis as a TRAIL sensitizer via activation of caspase 8 and death receptor 5 in human colon cancer cells. Biomedicines 8, 84. 10.3390/biomedicines8040084 32283836 PMC7235876

[B120] Lecomte N. Njardarson J. T. Nagorny P. Yang G. Downey R. Ouerfelli O. (2011). Emergence of potent inhibitors of metastasis in lung cancer via syntheses based on migrastatin. Proc. Natl. Acad. Sci. U. S. A. 108, 15074–15078. 10.1073/pnas.1015247108 21808037 PMC3174672

[B121] Lee C.-H. Ying T.-H. Chiou H.-L. Hsieh S.-C. Wen S.-H. Chou R.-H. (2017). Alpha-mangostin induces apoptosis through activation of reactive oxygen species and ASK1/p38 signaling pathway in cervical cancer cells. Oncotarget 8, 47425–47439. 10.18632/oncotarget.17659 28537893 PMC5564576

[B122] Lee D.-Y. Yun S.-M. Song M.-Y. Jung K. Kim E.-H. (2020a). Cyanidin chloride induces apoptosis by inhibiting NF-κB signaling through activation of Nrf2 in colorectal cancer cells. Antioxidants (Basel) 9, 285. 10.3390/antiox9040285 32230772 PMC7222181

[B123] Lee H.-P. Li T.-M. Tsao J.-Y. Fong Y.-C. Tang C.-H. (2012). Curcumin induces cell apoptosis in human chondrosarcoma through extrinsic death receptor pathway. Int. Immunopharmacol. 13, 163–169. 10.1016/j.intimp.2012.04.002 22522053

[B124] Lee M.-G. Lee K.-S. Nam K.-S. (2020b). Anti-metastatic effects of arctigenin are regulated by MAPK/AP-1 signaling in 4T-1 mouse breast cancer cells. Mol. Med. Rep. 21, 1374–1382. 10.3892/mmr.2020.10937 32016480

[B125] Lee M.-S. Yuet-Wa J. C. Kong S.-K. Yu B. Eng-Choon V. O. Nai-Ching H. W. (2005). Effects of polyphyllin D, a steroidal saponin in Paris polyphylla, in growth inhibition of human breast cancer cells and in xenograft. Cancer Biol. Ther. 4, 1248–1254. 10.4161/cbt.4.11.2136 16258257

[B126] Li C. Yang X. Chen C. Cai S. Hu J. (2014). Isorhamnetin suppresses colon cancer cell growth through the PI3K-Akt-mTOR pathway. Mol. Med. Rep. 9, 935–940. 10.3892/mmr.2014.1886 24398569

[B127] Li D. Wang G. Jin G. Yao K. Zhao Z. Bie L. (2019). Resveratrol suppresses colon cancer growth by targeting the AKT/STAT3 signaling pathway. Int. J. Mol. Med. 43, 630–640. 10.3892/ijmm.2018.3969 30387805

[B128] Li H. Du G. Yang L. Pang L. Zhan Y. (2020). The antitumor effects of britanin on hepatocellular carcinoma cells and its real-time evaluation by *in vivo* bioluminescence imaging. Anti-cancer agents Med. Chem. 20, 1147–1156. 10.2174/1871520620666200227092623 32106805

[B129] Li M. Beg A. A. (2000). Induction of necrotic-like cell death by tumor necrosis factor alpha and caspase inhibitors: novel mechanism for killing virus-infected cells. J. virology 74, 7470–7477. 10.1128/jvi.74.16.7470-7477.2000 10906200 PMC112267

[B130] Li R. Inbaraj B. S. Chen B.-H. (2023). Quantification of xanthone and anthocyanin in mangosteen peel by UPLC-MS/MS and preparation of nanoemulsions for studying their inhibition effects on liver cancer cells. Int. J. Mol. Sci. 24, 3934. 10.3390/ijms24043934 36835343 PMC9965517

[B131] Li S. Chen J. Fan Y. Wang C. Wang C. Zheng X. (2022). Liposomal Honokiol induces ROS-mediated apoptosis via regulation of ERK/p38-MAPK signaling and autophagic inhibition in human medulloblastoma. Signal Transduct. Target. Ther. 7, 49. 10.1038/s41392-021-00869-w 35185151 PMC8858958

[B132] Li X. Zhao J. Yan T. Mu J. Lin Y. Chen J. (2021). Cyanidin-3-O-glucoside and cisplatin inhibit proliferation and downregulate the PI3K/AKT/mTOR pathway in cervical cancer cells. J. food Sci. 86, 2700–2712. 10.1111/1750-3841.15740 33908630

[B133] Liang F. Lv Y. Qiao X. Zhang S. Shen S. Wang C. (2023). Cinchonine-induced cell death in pancreatic cancer cells by downregulating RRP15. Cell Biol. Int. 47, 907–919. 10.1002/cbin.11987 36682038

[B134] Liang X. Wang P. Yang C. Huang F. Wu H. Shi H. (2021). Galangin inhibits gastric cancer growth through enhancing STAT3 mediated ROS production. Front. Pharmacol. 12, 646628. 10.3389/fphar.2021.646628 33981228 PMC8109028

[B135] Lim W. Jeong M. Bazer F. W. Song G. (2016). Curcumin suppresses proliferation and migration and induces apoptosis on human placental choriocarcinoma cells via ERK1/2 and SAPK/JNK MAPK signaling pathways. Biol. reproduction 95, 83. 10.1095/biolreprod.116.141630 27580989

[B136] Lin F.-L. Hsu J.-L. Chou C.-H. Wu W.-J. Chang C.-I. Liu H.-J. (2011). Activation of p38 MAPK by damnacanthal mediates apoptosis in SKHep 1 cells through the DR5/TRAIL and TNFR1/TNF-α and p53 pathways. Eur. J. Pharmacol. 650, 120–129. 10.1016/j.ejphar.2010.10.005 20951126

[B137] Lin H.-F. Hsieh M.-J. Hsi Y.-T. Lo Y.-S. Chuang Y.-C. Chen M.-K. (2017). Celastrol-induced apoptosis in human nasopharyngeal carcinoma is associated with the activation of the death receptor and the mitochondrial pathway. Oncol. Lett. 14, 1683–1690. 10.3892/ol.2017.6346 28789395 PMC5529953

[B138] Liu M.-J. Wang Z. Ju Y. Wong R. N.-S. Wu Q.-Y. (2005). Diosgenin induces cell cycle arrest and apoptosis in human leukemia K562 cells with the disruption of Ca2+ homeostasis. Cancer Chemother. Pharmacol. 55, 79–90. 10.1007/s00280-004-0849-3 15372201

[B139] Liu Q. Xu X. Zhao M. Wei Z. Li X. Zhang X. (2015). Berberine induces senescence of human glioblastoma cells by downregulating the EGFR-MEK-ERK signaling pathway. Mol. cancer Ther. 14, 355–363. 10.1158/1535-7163.MCT-14-0634 25504754

[B140] Liu T. He Y. Liao Y. (2024). Anacardic acid inhibits the proliferation and inflammation of HaCaT cells induced by TNF- α via the regulation of NF - κB pathway. Trop. J. Pharm. Res. 23, 251–256. 10.4314/tjpr.v23i2.3

[B141] Lu J.-J. Bao J.-L. Chen X.-P. Huang M. Wang Y.-T. (2012). Alkaloids isolated from natural herbs as the anticancer agents. Evidence-based complementary Altern. Med. 2012, 485042. 10.1155/2012/485042 PMC3440018 22988474

[B142] Łukasiewicz S. Czeczelewski M. Forma A. Baj J. Sitarz R. Stanisławek A. (2021). Breast cancer-epidemiology, risk factors, classification, prognostic markers, and current treatment strategies-an updated review. Cancers 13, 4287. 10.3390/cancers13174287 34503097 PMC8428369

[B143] Luo K. W. Lung W. Y. Chun-Xie Luo X. L. Huang W. R. (2018). EGCG inhibited bladder cancer T24 and 5637 cell proliferation and migration via PI3K/AKT pathway. Oncotarget 9, 12261–12272. 10.18632/oncotarget.24301 29552308 PMC5844744

[B144] Madureira M. B. Concato V. M. Cruz E. M. S. Bitencourt de Morais J. M. Inoue F. S. R. Concimo Santos N. (2023). Naringenin and hesperidin as promising alternatives for prevention and Co-adjuvant therapy for breast cancer. Antioxidants (Basel) 12, 586. 10.3390/antiox12030586 36978836 PMC10045673

[B145] Mahmoud M. A. Okda T. M. Omran G. A. Abd-Alhaseeb M. M. (2021). Rosmarinic acid suppresses inflammation, angiogenesis, and improves paclitaxel induced apoptosis in a breast cancer model via NF3 κB-p53-caspase-3 pathways modulation. J. Appl. Biomed. 19, 202–209. 10.32725/jab.2021.024 34907739

[B146] Mak T. W. Yeh W.-C. (2002). Signaling for survival and apoptosis in the immune system. Arthritis Res. 4 (Suppl. 3), S243–S252. 10.1186/ar569 12110144 PMC3240145

[B147] Mari A. Mani G. Nagabhishek S. N. Balaraman G. Subramanian N. Mirza F. B. (2021). Carvacrol promotes cell cycle arrest and apoptosis through PI3K/AKT signaling pathway in MCF-7 breast cancer cells. Chin. J. Integr. Med. 27, 680–687. 10.1007/s11655-020-3193-5 32572774

[B148] Martemucci G. Costagliola C. Mariano M. D’andrea L. Napolitano P. D’Alessandro A. G. (2022). Free radical properties, source and targets, antioxidant consumption and health. Oxygen 2, 48–78. 10.3390/oxygen2020006

[B149] Martínez-Limón A. Joaquin M. Caballero M. Posas F. de Nadal E. (2020). The p38 pathway: from biology to cancer therapy. Int. J. Mol. Sci. 21, 1913. 10.3390/ijms21061913 32168915 PMC7139330

[B150] Maxwell T. Lee K. S. Kim S. Nam K.-S. (2018). Arctigenin inhibits the activation of the mTOR pathway, resulting in autophagic cell death and decreased ER expression in ER-positive human breast cancer cells. Int. J. Oncol. 52, 1339–1349. 10.3892/ijo.2018.4271 29436614

[B151] Mendonca P. Soliman K. F. A. (2020). Flavonoids activation of the transcription factor Nrf2 as a hypothesis approach for the prevention and modulation of SARS-CoV-2 infection severity. Antioxidants (Basel) 9, 659. 10.3390/antiox9080659 32722164 PMC7463602

[B152] Meng X. Shao Z. (2021). Ambrosin exerts strong anticancer effects on human breast cancer cells via activation of caspase and inhibition of the Wnt/β-catenin pathway. Trop. J. Pharm. Res. 20, 809–814. 10.4314/tjpr.v20i4.22

[B153] Mi X.-J. Choi H. S. Perumalsamy H. Shanmugam R. Thangavelu L. Balusamy S. R. (2022). Biosynthesis and cytotoxic effect of silymarin-functionalized selenium nanoparticles induced autophagy mediated cellular apoptosis via downregulation of PI3K/Akt/mTOR pathway in gastric cancer. Phytomedicine 99, 154014. 10.1016/j.phymed.2022.154014 35247670

[B154] Miatmoko A. Mianing E. A. Sari R. Hendradi E. (2021). Nanoparticles use for delivering ursolic acid in cancer therapy: a scoping review. Front. Pharmacol. 12, 787226. 10.3389/fphar.2021.787226 35002719 PMC8740088

[B155] Mijit M. Caracciolo V. Melillo A. Amicarelli F. Giordano A. (2020). Role of p53 in the regulation of cellular senescence. Biomolecules 10, 420. 10.3390/biom10030420 32182711 PMC7175209

[B156] Miller M. A. Zachary J. F. (2017). “Mechanisms and morphology of cellular injury, adaptation, and death,” in Pathologic basis of veterinary disease. Editor Zachary J. F. (Elsevier), 2–43 e19. 10.1016/B978-0-323-35775-3.00001-1

[B157] Min H.-Y. Lee H.-Y. (2023). Cellular dormancy in cancer: mechanisms and potential targeting strategies. Cancer Res. Treat. 55, 720–736. 10.4143/crt.2023.468 36960624 PMC10372609

[B158] Mukhtar E. Adhami V. M. Sechi M. Mukhtar H. (2015). Dietary flavonoid fisetin binds to β-tubulin and disrupts microtubule dynamics in prostate cancer cells. Cancer Lett. 367, 173–183. 10.1016/j.canlet.2015.07.030 26235140 PMC4570246

[B159] Musial C. Kuban-Jankowska A. Gorska-Ponikowska M. (2020). Beneficial properties of green tea catechins. Int. J. Mol. Sci. 21, 1744. 10.3390/ijms21051744 32143309 PMC7084675

[B160] Ni J. Wen X. Yao J. Chang H.-C. Yin Y. Zhang M. (2005). Tocopherol-associated protein suppresses prostate cancer cell growth by inhibition of the phosphoinositide 3-kinase pathway. Cancer Res. 65, 9807–9816. 10.1158/0008-5472.CAN-05-1334 16267002

[B161] Noori-Daloii M. R. Momeny M. Yousefi M. Shirazi F. G. Yaseri M. Motamed N. (2011). Multifaceted preventive effects of single agent quercetin on a human prostate adenocarcinoma cell line (PC-3): implications for nutritional transcriptomics and multi-target therapy. Med. Oncol. N. Lond. Engl. 28, 1395–1404. 10.1007/s12032-010-9603-3 20596804

[B162] Nozhat Z. Heydarzadeh S. Memariani Z. Ahmadi A. (2021). Chemoprotective and chemosensitizing effects of apigenin on cancer therapy. Cancer Cell Int. 21, 574. 10.1186/s12935-021-02282-3 34715860 PMC8555304

[B163] Olofinsan K. Abrahamse H. George B. P. (2023). Therapeutic role of alkaloids and alkaloid derivatives in cancer management. Molecules 28, 5578. 10.3390/molecules28145578 37513450 PMC10386240

[B164] Pai J.-T. Hsu M.-W. Leu Y.-L. Chang K.-T. Weng M.-S. (2021). Induction of G2/M cell cycle arrest via p38/p21(Waf1/Cip1)-Dependent signaling pathway activation by bavachinin in non-small-cell lung cancer cells. Molecules 26, 5161. 10.3390/molecules26175161 34500594 PMC8434044

[B165] Pandey N. Tyagi G. Kaur P. Pradhan S. Rajam M. V. Srivastava T. (2020). Allicin overcomes hypoxia mediated cisplatin resistance in lung cancer cells through ROS mediated cell death pathway and by suppressing hypoxia inducible factors. Cell. physiology Biochem. 54, 748–766. 10.33594/000000253 32809300

[B166] Pang X. He X. Qiu Z. Zhang H. Xie R. Liu Z. (2023). Targeting integrin pathways: mechanisms and advances in therapy. Signal Transduct. Target. Ther. 8, 1. 10.1038/s41392-022-01259-6 36588107 PMC9805914

[B167] Park C. Jeong J.-W. Han M. H. Lee H. Kim G.-Y. Jin S. (2021). The anti-cancer effect of betulinic acid in u937 human leukemia cells is mediated through ROS-dependent cell cycle arrest and apoptosis. Animal cells Syst. 25, 119–127. 10.1080/19768354.2021.1915380 PMC8118407 34234893

[B168] Park M.-R. Kim S.-G. Cho I.-A. Oh D. Kang K.-R. Lee S.-Y. (2015). Licochalcone-A induces intrinsic and extrinsic apoptosis via ERK1/2 and p38 phosphorylation-mediated TRAIL expression in head and neck squamous carcinoma FaDu cells. Food Chem. Toxicol. 77, 34–43. 10.1016/j.fct.2014.12.013 25572524 PMC4522946

[B169] Patel K. Patel D. (2021). Biological activity of ascaridole for the treatment of cancers: phytopharmaceutical importance with molecular study. Ann. Hepato-Biliary-Pancreatic Surg. 25, S294. 10.14701/ahbps.EP-93

[B170] Peng R. Xu M. Xie B. Min Q. Hui S. Du Z. (2023). Insights on antitumor activity and mechanism of natural benzophenanthridine alkaloids. Molecules 28, 6588. 10.3390/molecules28186588 37764364 PMC10535962

[B171] Pirpour Tazehkand A. Salehi R. Velaei K. Samadi N. (2020). The potential impact of trigonelline loaded micelles on Nrf2 suppression to overcome oxaliplatin resistance in colon cancer cells. Mol. Biol. Rep. 47, 5817–5829. 10.1007/s11033-020-05650-w 32661875

[B172] Pizzi A. (2021). Tannins medical/pharmacological and related applications: a critical review. Sustain. Chem. Pharm. 22, 100481. 10.1016/j.scp.2021.100481

[B173] Qi X. Jha S. K. Jha N. K. Dewanjee S. Dey A. Deka R. (2022). Antioxidants in brain tumors: current therapeutic significance and future prospects. Mol. cancer 21, 204. 10.1186/s12943-022-01668-9 36307808 PMC9615186

[B174] Qin R. You F.-M. Zhao Q. Xie X. Peng C. Zhan G. (2022). Naturally derived indole alkaloids targeting regulated cell death (RCD) for cancer therapy: from molecular mechanisms to potential therapeutic targets. J. Hematol. Oncol. 15, 133. 10.1186/s13045-022-01350-z 36104717 PMC9471064

[B175] Quideau S. Jourdes M. Lefeuvre D. Montaudon D. Saucier C. Glories Y. (2005). The chemistry of wine polyphenolic C-glycosidic ellagitannins targeting human topoisomerase II. Chem. 11, 6503–6513. 10.1002/chem.200500428 16110520

[B176] Rahmani A. H. Almatroudi A. Allemailem K. S. Alwanian W. M. Alharbi B. F. Alrumaihi F. (2023). Myricetin: a significant emphasis on its anticancer potential via the modulation of inflammation and signal transduction pathways. Int. J. Mol. Sci. 24, 9665. 10.3390/ijms24119665 37298616 PMC10253333

[B177] Rahmani A. H. Alsahli M. A. Almatroudi A. Almogbel M. A. Khan A. A. Anwar S. (2022). The potential role of apigenin in cancer prevention and treatment. Molecules 27, 6051. 10.3390/molecules27186051 36144783 PMC9505045

[B178] Ratan Z. A. Haidere M. F. Nurunnabi M. Shahriar S. M. Ahammad A. J. S. Shim Y. Y. (2020). Green chemistry synthesis of silver nanoparticles and their potential anticancer effects. Cancers 12, 855. 10.3390/cancers12040855 32244822 PMC7226404

[B179] Ray R. Saha S. Paul S. (2022). Two novel compounds, ergosterol and ergosta-5,8-dien-3-ol, from Termitomyces heimii Natarajan demonstrate promising anti-hepatocarcinoma activity. J. Traditional Chin. Med. Sci. 9, 443–453. 10.1016/j.jtcms.2022.09.006

[B180] Reddy D. Kumavath R. Barh D. Azevedo V. Ghosh P. (2020). Anticancer and antiviral properties of cardiac glycosides: a review to explore the mechanism of actions. Molecules 25, 3596. 10.3390/molecules25163596 32784680 PMC7465415

[B181] Rigby C. M. Roy S. Deep G. Guillermo-Lagae R. Jain A. K. Dhar D. (2017). Role of p53 in silibinin-mediated inhibition of ultraviolet B radiation-induced DNA damage, inflammation and skin carcinogenesis. Carcinogenesis 38, 40–50. 10.1093/carcin/bgw106 27729375 PMC5219048

[B182] Safi A. Heidarian E. Ahmadi R. (2021). Quercetin synergistically enhances the anticancer efficacy of docetaxel through induction of apoptosis and modulation of PI3K/AKT, MAPK/ERK, and JAK/STAT3 signaling pathways in MDA-MB-231 breast cancer cell line. Int. J. Mol. Cell. Med. 10, 11–22. 10.22088/IJMCM.BUMS.10.1.11 34268250 PMC8256834

[B183] Sali V. K. Mani S. Meenaloshani G. Velmurugan Ilavarasi A. Vasanthi H. R. (2020). Type 5 17-hydroxysteroid dehydrogenase/prostaglandin F synthase (AKR1C3) inhibition and potential anti-proliferative activity of cholest-4-ene-3,6-dione in MCF-7 breast cancer cells. Steroids 159, 108638. 10.1016/j.steroids.2020.108638 32209376

[B184] Sanchez-Martin V. Plaza-Calonge M. D. C. Soriano-Lerma A. Ortiz-Gonzalez M. Linde-Rodriguez A. Perez-Carrasco V. (2022). Gallic acid: a natural phenolic compound exerting antitumoral activities in colorectal cancer via interaction with G-quadruplexes. Cancers 14, 2648. 10.3390/cancers14112648 35681628 PMC9179882

[B185] Santarpia L. Lippman S. M. El-Naggar A. K. (2012). Targeting the MAPK-RAS-RAF signaling pathway in cancer therapy. Expert Opin. Ther. targets 16, 103–119. 10.1517/14728222.2011.645805 22239440 PMC3457779

[B186] Schiliro C. Firestein B. L. (2021). Mechanisms of metabolic reprogramming in cancer cells supporting enhanced growth and proliferation. Cells 10, 1056. 10.3390/cells10051056 33946927 PMC8146072

[B187] Schneider-Brachert W. Heigl U. Ehrenschwender M. (2013). Membrane trafficking of death receptors: implications on signalling. Int. J. Mol. Sci. 14, 14475–14503. 10.3390/ijms140714475 23852022 PMC3742255

[B188] Senthamizh N. Vidhyavathi R. Raj E. Lobo V. Ramu A. (2020). Reserpine subdued non-small cell lung cancer cells via ROS-mediated apoptosis. J. Conventional Knowl. Holist. Health 4, 1–4. 10.53517/JCKHH.2581-3331.422020206

[B189] Shen J. Yang Z. Wu X. Yao G. Hou M. (2023). Baicalein facilitates gastric cancer cell apoptosis by triggering endoplasmic reticulum stress via repression of the PI3K/AKT pathway. Appl. Biol. Chem. 66, 10. 10.1186/s13765-022-00759-x 36815904 PMC9924871

[B190] Shi R.-X. Ong C.-N. Shen H.-M. (2004). Luteolin sensitizes tumor necrosis factor-alpha-induced apoptosis in human tumor cells. Oncogene 23, 7712–7721. 10.1038/sj.onc.1208046 15334063

[B191] Shiau J.-P. Chuang Y.-T. Tang J.-Y. Yang K.-H. Chang F.-R. Hou M.-F. (2022). The impact of oxidative stress and AKT pathway on cancer cell functions and its application to natural products. Antioxidants 11, 1845. 10.3390/antiox11091845 36139919 PMC9495789

[B192] Si L. Fu J. Liu W. Hayashi T. Nie Y. Mizuno K. (2020). Silibinin inhibits migration and invasion of breast cancer MDA-MB-231 cells through induction of mitochondrial fusion. Mol. Cell. Biochem. 463, 189–201. 10.1007/s11010-019-03640-6 31612353

[B193] Siegmund D. Kums J. Ehrenschwender M. Wajant H. (2016). Activation of TNFR2 sensitizes macrophages for TNFR1-mediated necroptosis. Cell death Dis. 7, e2375. 10.1038/cddis.2016.285 27899821 PMC5059883

[B194] Silva L. C. Borgato G. B. Wagner V. P. Martins M. D. Rocha G. Z. Lopes M. A. (2022). Cephaeline is an inductor of histone H3 acetylation and inhibitor of mucoepidermoid carcinoma cancer stem cells. J. oral pathology Med. 51, 553–562. 10.1111/jop.13252 PMC9013730 34661317

[B195] Simanullang R. H. Situmorang P. C. Herlina M. Noradina S. B. Manurung S. S. (2022a). Histological changes of cervical tumours following *Zanthoxylum acanthopodium* DC treatment, and its impact on cytokine expression. Saudi J. Biol. Sci. 29 (4), 2706–2718. 10.1016/j.sjbs.2021.12.065 35531208 PMC9073070

[B196] Simanullang R. H. Situmorang P. C. Herlina M. N. Silalahi B. (2022c). Cytochrome c expression by andaliman (*Zanthoxylum acanthopodium*) on cervical cancer histology. Pak J. Biol. Sci. 25 (1), 49–55. 10.3923/pjbs.2022.49.55 35001575

[B197] Simanullang R. H. Situmorang P. C. Siahaan J. M. Widjaja S. S. Mutiara M. (2022b). Effects of *Zanthoxylum acanthopodium* on MMP-9 and GLUT-1 expression and histology changes in rats with cervical carcinoma. Pharmacia 69 (4), 911–920. 10.3897/pharmacia.69.e89368

[B198] Singh A. K. Vinayak M. (2017). Resveratrol alleviates inflammatory hyperalgesia by modulation of reactive oxygen species (ROS), antioxidant enzymes and ERK activation. Inflamm. Res. 66, 911–921. 10.1007/s00011-017-1072-0 28647835

[B199] Situmorang P. C. Ilyas S. (2018). Review: germinal cell apoptosis by herbal medicine. Asian J Pharm. Clin. Res. 11 (9), 24–31. 10.22159/ajpcr.2018.v11i9.26400

[B200] Situmorang P. C. Ilyas S. Hutahaean S. Rosidah R. (2021a). Components and acute toxicity of nano herbal haramonting (Rhodomyrtus tomentosa). J. Herbmed Pharmacol. 10, 139–148. 10.34172/jhp.2021.15

[B201] Situmorang P. C. Ilyas S. Hutahaean S. Rosidah R. (2021b). Effect of nano herbal andaliman (*Zanthoxylum acanthopodium*) fruits in NOTCH1 and Hes1 expressions to human placental trophoblasts. Pak J. Biol. Sci. 24 (1), 165–171. 10.3923/pjbs.2021.165.171 33683044

[B202] Situmorang P. C. Ilyas S. Hutahaean S. Rosidah R. (2021c). Histological changes in placental rat apoptosis via FasL and cytochrome c by the nano-herbal *Zanthoxylum acanthopodium* . Saudi J. Bio Sci. 28 (5), 3060–3068. 10.1016/j.sjbs.2021.02.047 34025182 PMC8117027

[B203] Situmorang P. C. Ilyas S. Siahaan D. A. S. Restuati M. Sari E. R. Chairunisa C. (2022b). Effect of *Rhodomyrtus tomentosa* Hassk. on HIF1α and VEGF expressions on hypertension placental. J. Pharm. Pharmacogn. Res. 10 (6), 1076–1086. 10.56499/jppres22.1517_10.6.1076

[B204] Situmorang P. C. Ilyas S. Syahputra R. A. Nugraha A. P. Putri M. S. S. Rumahorbo C. G. P. (2024a). Rhodomyrtus tomentosa (Aiton) Hassk. (haramonting) protects against allethrin-exposed pulmo damage in rats:mechanistic interleukins. Front. Pharmacol. 15, 1343936. 10.3389/fphar.2024.1343936 38379903 PMC10877004

[B205] Situmorang P. C. Ilyas S. Syahputra R. A. Sari R. M. Nugraha A. P. Ibrahim A. (2024b). *Rhodomyrtus tomentosa* as a new anticancer molecular strategy in breast histology via Her2, IL33, EGFR, and MUC1. Front. Pharmacol. 15, 1345645. 10.3389/fphar.2024.1345645 38476328 PMC10927741

[B206] Situmorang P. C. Simanullang R. H. Syahputra R. A. Hutahaean M. M. Sembiring H. Nisfa L. (2023). Histological analysis of TGFβ1 and VEGFR expression in cervical carcinoma treated with *Rhodomyrtus tomentosa* . Pharmacia 70 (1), 217–223. 10.3897/pharmacia.70.e96811

[B207] Situmorang P. C. Syahputra R A. Simanullang R. H. (2022a). EGFL7 and HIF-1*a* expression on human trophoblast placental by *Rhodomyrtus tomentosa* and *Zanthoxylum acanthopodium* . Pak J. Biol. Sci. 25 (2), 123–130. 10.3923/pjbs.2022.123.130 35234000

[B208] Škubník J. Pavlíčková V. S. Ruml T. Rimpelová S. (2021). Vincristine in combination therapy of cancer: emerging trends in clinics. Biology 10, 849. 10.3390/biology10090849 34571726 PMC8468923

[B209] Sohel M. Sultana H. Sultana T. Al Amin M. Aktar S. Ali M. C. (2022). Chemotherapeutic potential of hesperetin for cancer treatment, with mechanistic insights: a comprehensive review. Heliyon 8, e08815. 10.1016/j.heliyon.2022.e08815 35128104 PMC8810372

[B210] Son H.-K. Kim D. (2023). Quercetin induces cell cycle arrest and apoptosis in YD10B and YD38 oral squamous cell carcinoma cells. Asian Pac. J. cancer Prev. APJCP 24, 283–289. 10.31557/APJCP.2023.24.1.283 36708578 PMC10152863

[B211] Srinivasan S. Koduru S. Kumar R. Venguswamy G. Kyprianou N. Damodaran C. (2009). Diosgenin targets Akt-mediated prosurvival signaling in human breast cancer cells. Int. J. cancer 125, 961–967. 10.1002/ijc.24419 19384950

[B212] Subramaniam A. Loo S. Y. Rajendran P. Manu K. A. Perumal E. Li F. (2013). An anthraquinone derivative, emodin sensitizes hepatocellular carcinoma cells to TRAIL induced apoptosis through the induction of death receptors and downregulation of cell survival proteins. Apoptosis Int. J. Program. Cell death 18, 1175–1187. 10.1007/s10495-013-0851-5 23700228

[B213] Sun L. Yuan W. Wen G. Yu B. Xu F. Gan X. (2020). Parthenolide inhibits human lung cancer cell growth by modulating the IGF-1R/PI3K/Akt signaling pathway. Oncol. Rep. 44, 1184–1193. 10.3892/or.2020.7649 32705224

[B214] Sun M.-Y. Bhaskar S. M. M. (2022). When two maladies meet: disease burden and pathophysiology of stroke in cancer. Int. J. Mol. Sci. 23, 15769. 10.3390/ijms232415769 36555410 PMC9779017

[B215] Suo F. Zhou X. Setroikromo R. Quax W. J. (2022). Receptor specificity engineering of TNF superfamily ligands. Pharmaceutics 14, 181. 10.3390/pharmaceutics14010181 35057080 PMC8781899

[B216] Takáč P. Michalková R. Čižmáriková M. Bedlovičová Z. Balážová Ľ. Takáčová G. (2023). The role of silver nanoparticles in the diagnosis and treatment of cancer: are there any perspectives for the future? Life (Basel) 13, 466. 10.3390/life13020466 36836823 PMC9965924

[B217] Talib W. H. Alsalahat I. Daoud S. Abutayeh R. F. Mahmod A. I. (2020). Plant-derived natural products in cancer research: extraction, mechanism of action, and drug formulation. Molecules 25, 5319. 10.3390/molecules25225319 33202681 PMC7696819

[B218] Tayarani-Najaran Z. Tayarani-Najaran N. Eghbali S. (2021). A review of auraptene as an anticancer agent. Front. Pharmacol. 12, 698352. 10.3389/fphar.2021.698352 34239445 PMC8258114

[B219] Tayeh M. Watanapokasin R. (2020). Antimetastatic potential of rhodomyrtone on human chondrosarcoma SW1353 cells. Evidence-based complementary Altern. Med. eCAM 2020, 8180261. 10.1155/2020/8180261 PMC7403900 32802134

[B220] Tian Z. Sun C. Liu J. (2022). Pelargonidin inhibits vascularization and metastasis of brain gliomas by blocking the PI3K/AKT/mTOR pathway. J. Biosci. 47, 64. 10.1007/s12038-022-00281-8 36226369

[B221] Tran S. Fairlie W. D. Lee E. F. (2021). BECLIN1: protein structure, function and regulation. Cells 10, 1522. 10.3390/cells10061522 34204202 PMC8235419

[B222] Tsai C.-H. Yang C.-W. Wang J.-Y. Tsai Y.-F. Tseng L.-M. King K.-L. (2013). Timosaponin AIII suppresses hepatocyte growth factor-induced invasive activity through sustained ERK activation in breast cancer MDA-MB-231 cells. Evidence-based complementary Altern. Med. eCAM 2013, 421051. 10.1155/2013/421051 PMC3708436 23878598

[B223] Tyagi A. Sharma S. Wu K. Wu S.-Y. Xing F. Liu Y. (2021). Nicotine promotes breast cancer metastasis by stimulating N2 neutrophils and generating pre-metastatic niche in lung. Nat. Commun. 12, 474. 10.1038/s41467-020-20733-9 33473115 PMC7817836

[B224] Verma A. K. Bharti P. S. Rafat S. Bhatt D. Goyal Y. Pandey K. K. (2021). Autophagy paradox of cancer: role, regulation, and duality. Oxidative Med. Cell. Longev. 2021, 8832541. 10.1155/2021/8832541 PMC7892237 33628386

[B225] Villareal M. O. Chaochaiphat T. Bejaoui M. Sato K. Isoda H. City T. (2020). Tara tannin regulates pigmentation by modulating melanogenesis enzymes and melanosome transport proteins expression. Planta Medica Int. Open 7 (01), e34–e44. 10.1055/a-1141-0151

[B226] Vodanovich D. A. M Choong P. F. (2018). Soft-tissue sarcomas. Indian J. Orthop. 52, 35–44. 10.4103/ortho.IJOrtho_220_17 29416168 PMC5791230

[B227] Wahi A. Bishnoi M. Raina N. Singh M. A. Verma P. Gupta P. K. (2023). Recent updates on nano-phyto-formulations based therapeutic intervention for cancer treatment. Oncol. Res. 32, 19–47. 10.32604/or.2023.042228 38188681 PMC10767243

[B228] Wang B.-F. Wang X.-J. Kang H.-F. Bai M.-H. Guan H.-T. Wang Z.-W. (2014a). Saikosaponin-D enhances radiosensitivity of hepatoma cells under hypoxic conditions by inhibiting hypoxia-inducible factor-1α. Cell. physiology Biochem. 33, 37–51. 10.1159/000356648 24401554

[B229] Wang F. Chang Z. Fan Q. Wang L. (2014b). Epigallocatechin-3-gallate inhibits the proliferation and migration of human ovarian carcinoma cells by modulating p38 kinase and matrix metalloproteinase-2. Mol. Med. Rep. 9, 1085–1089. 10.3892/mmr.2014.1909 24452912

[B230] Wang J. Li J. Cao N. Li Z. Han J. Li L. (2018). Resveratrol, an activator of SIRT1, induces protective autophagy in non-small-cell lung cancer via inhibiting Akt/mTOR and activating p38-MAPK. OncoTargets Ther. 11, 7777–7786. 10.2147/OTT.S159095 PMC6223384 30464525

[B231] Wang M. Yu F. Zhang Y. Chang W. Zhou M. (2022). The effects and mechanisms of flavonoids on cancer prevention and therapy: focus on gut microbiota. Int. J. Biol. Sci. 18, 1451–1475. 10.7150/ijbs.68170 35280689 PMC8898378

[B232] Wei L. Jin X. Cao Z. Li W. (2016). Evodiamine induces extrinsic and intrinsic apoptosis of ovarian cancer cells via the mitogen-activated protein kinase/phosphatidylinositol-3-kinase/protein kinase B signaling pathways. J. traditional Chin. Med. 36, 353–359. 10.1016/s0254-6272(16)30049-8 27468551

[B233] Wei M. Li J. Qiu J. Yan Y. Wang H. Wu Z. (2020). Costunolide induces apoptosis and inhibits migration and invasion in H1299 lung cancer cells. Oncol. Rep. 43, 1986–1994. 10.3892/or.2020.7566 32236584 PMC7160540

[B234] Weledji E. P. Orock G. E. (2015). Surgery for non-hodgkin’s lymphoma. Oncol. Rev. 9, 274. 10.4081/oncol.2015.274 26779310 PMC4698592

[B235] Wolfrum P. Fietz A. Schnichels S. Hurst J. (2022). The function of p53 and its role in Alzheimer’s and Parkinson’s disease compared to age-related macular degeneration. Front. Neurosci. 16, 1029473. 10.3389/fnins.2022.1029473 36620455 PMC9811148

[B236] Woo S.-M. Choi Y. K. Kim A. J. Cho S.-G. Ko S.-G. (2016). p53 causes butein-mediated apoptosis of chronic myeloid leukemia cells. Mol. Med. Rep. 13, 1091–1096. 10.3892/mmr.2015.4672 26676515 PMC4732842

[B237] Wróblewska-Łuczka P. Cabaj J. Bargieł J. Łuszczki J. J. (2023). Anticancer effect of terpenes: focus on malignant melanoma. Pharmacol. Rep. P. R. 75, 1115–1125. 10.1007/s43440-023-00512-1 PMC10539410 37515699

[B238] Wu D. Liu Z. Li J. Zhang Q. Zhong P. Teng T. (2019). Epigallocatechin-3-gallate inhibits the growth and increases the apoptosis of human thyroid carcinoma cells through suppression of EGFR/RAS/RAF/MEK/ERK signaling pathway. Cancer Cell Int. 19, 43. 10.1186/s12935-019-0762-9 30858760 PMC6394055

[B239] Wu H. Du J. Li C. Li H. Guo H. Li Z. (2022a). Kaempferol can reverse the 5-fu resistance of colorectal cancer cells by inhibiting PKM2-mediated glycolysis. Int. J. Mol. Sci. 23, 3544. 10.3390/ijms23073544 35408903 PMC8998549

[B240] Wu M.-F. Huang Y.-H. Chiu L.-Y. Cherng S.-H. Sheu G.-T. Yang T.-Y. (2022b). Curcumin induces apoptosis of chemoresistant lung cancer cells via ROS-regulated p38 MAPK phosphorylation. Int. J. Mol. Sci. 23, 8248. 10.3390/ijms23158248 35897820 PMC9367815

[B241] Xi H. Wang S. Wang B. Hong X. Liu X. Li M. (2022). The role of interaction between autophagy and apoptosis in tumorigenesis (Review). Oncol. Rep. 48, 208. 10.3892/or.2022.8423 36222296 PMC9579747

[B242] Xiao J. Gao M. Fei B. Huang G. Diao Q. (2020). Nature-derived anticancer steroids outside cardica glycosides. Fitoterapia 147, 104757. 10.1016/j.fitote.2020.104757 33069834

[B243] Xie X. Zhang L. Zhang W. Tayebee R. Hoseininasr A. Vatanpour H. (2020). Fabrication of temperature and pH sensitive decorated magnetic nanoparticles as effective biosensors for targeted delivery of acyclovir anti-cancer drug. J. Mol. Liq. 309, 113024. 10.1016/j.molliq.2020.113024

[B244] Yamada M. Iijima Y. Seo M. Hino S. Sano M. Sakagami H. (2023). Cancer chemotherapy-associated pigmentation of the oral mucosa. vivo (Athens, Greece) 37, 1880–1885. 10.21873/invivo.13280 PMC10347951 37369479

[B245] Yang J. Yang Y. Tian L. Sheng X.-F. Liu F. Cao J.-G. (2011). Casticin-induced apoptosis involves death receptor 5 upregulation in hepatocellular carcinoma cells. World J. gastroenterology 17, 4298–4307. 10.3748/wjg.v17.i38.4298 PMC3214705 22090786

[B246] Yang L. Zhang W. Chopra S. Kaur D. Wang H. Li M. (2020). The epigenetic modification of epigallocatechin gallate (EGCG) on cancer. Curr. drug targets 21, 1099–1104. 10.2174/1389450121666200504080112 32364072

[B247] Yao H. Chen Y. Zhang L. He X. He X. Lian L. (2014). Carnosol inhibits cell adhesion molecules and chemokine expression by tumor necrosis factor-α in human umbilical vein endothelial cells through the nuclear factor-κB and mitogen-activated protein kinase pathways. Mol. Med. Rep. 9, 476–480. 10.3892/mmr.2013.1839 24316968

[B248] You Y. Wang R. Shao N. Zhi F. Yang Y. (2019). Luteolin suppresses tumor proliferation through inducing apoptosis and autophagy via MAPK activation in glioma. OncoTargets Ther. 12, 2383–2396. 10.2147/OTT.S191158 PMC6445239 30992674

[B249] Yuan B. Yang R. Ma Y. Zhou S. Zhang X. Liu Y. (2017). A systematic review of the active saikosaponins and extracts isolated from Radix Bupleuri and their applications. Pharm. Biol. 55, 620–635. 10.1080/13880209.2016.1262433 27951737 PMC6130612

[B250] Yuan M. Zhang G. Bai W. Han X. Li C. Bian S. (2022). The role of bioactive compounds in natural products extracted from plants in cancer treatment and their mechanisms related to anticancer effects. Oxidative Med. Cell. Longev. 2022, 1429869. 10.1155/2022/1429869 PMC8863487 35211240

[B251] Yusuf A. Almotairy A. R. Z. Henidi H. Alshehri O. Y. Aldughaim M. S. (2023). Nanoparticles as drug delivery systems: a review of the implication of nanoparticles’ physicochemical properties on responses in biological systems. Polymers 15, 1596. 10.3390/polym15071596 37050210 PMC10096782

[B252] Zaafar D. Khalil H. M. A. Rasheed R. A. Eltelbany R. F. A. Zaitone S. A. (2022). Hesperetin mitigates sorafenib-induced cardiotoxicity in mice through inhibition of the TLR4/NLRP3 signaling pathway. PloS one 17, e0271631. 10.1371/journal.pone.0271631 35944026 PMC9362940

[B253] Zeng Q. Che Y. Zhang Y. Chen M. Guo Q. Zhang W. (2020). Thymol isolated from thymus vulgaris L. Inhibits colorectal cancer cell growth and metastasis by suppressing the wnt/β-catenin pathway. Drug Des. Dev. Ther. 14, 2535–2547. 10.2147/DDDT.S254218 PMC7335897 32669835

[B254] Zhang G. Li Z. Dong J. Zhou W. Zhang Z. Que Z. (2022). Acacetin inhibits invasion, migration and TGF-β1-induced EMT of gastric cancer cells through the PI3K/Akt/Snail pathway. BMC complementary Med. Ther. 22, 10. 10.1186/s12906-021-03494-w PMC8744305 35000605

[B255] Zhang L. Yang C. Huang Y. Huang H. Yuan X. Zhang P. (2021a). Cardamonin inhibits the growth of human osteosarcoma cells through activating P38 and JNK signaling pathway. Biomed. Pharmacother. = Biomedecine Pharmacother. 134, 111155. 10.1016/j.biopha.2020.111155 33370628

[B256] Zhang R. Hao J. Wu Q. Guo K. Wang C. Zhang W. K. (2020). Dehydrocostus lactone inhibits cell proliferation and induces apoptosis by PI3K/Akt/Bad and ERS signalling pathway in human laryngeal carcinoma. J. Cell. Mol. Med. 24, 6028–6042. 10.1111/jcmm.15131 32319208 PMC7294112

[B257] Zhang Y. Yuan B. Bian B. Zhao H. Kiyomi A. Hayashi H. (2021b). Cytotoxic effects of hellebrigenin and arenobufagin against human breast cancer cells. Front. Oncol. 11, 711220. 10.3389/fonc.2021.711220 34513690 PMC8427765

[B258] Zhao X. Liu Z. Ren Z. Wang H. Wang Z. Zhai J. (2020). Triptolide inhibits pancreatic cancer cell proliferation and migration via down-regulating PLAU based on network pharmacology of Tripterygium wilfordii Hook F. Eur. J. Pharmacol. 880, 173225. 10.1016/j.ejphar.2020.173225 32464191

[B259] Zhao X. Tao X. Xu L. Yin L. Qi Y. Xu Y. (2016). Dioscin induces apoptosis in human cervical carcinoma HeLa and SiHa cells through ROS-mediated DNA damage and the mitochondrial signaling pathway. Molecules 21, 730. 10.3390/molecules21060730 27271587 PMC6273920

[B260] Zhao Y. Pan H. Liu W. Liu E. Pang Y. Gao H. (2023). Menthol: an underestimated anticancer agent. Front. Pharmacol. 14, 1148790. 10.3389/fphar.2023.1148790 37007039 PMC10063798

[B261] Zhao Z. Jiang Y. Liu Z. Li Q. Gao T. Zhang S. (2021). Ampelopsin inhibits cell viability and metastasis in renal cell carcinoma by negatively regulating the PI3K/AKT signaling pathway. Evidence-based complementary Altern. Med. 2021, 4650566. 10.1155/2021/4650566 PMC8601800 34804180

[B262] Zhou B.-G. Wei C.-S. Zhang S. Zhang Z. Gao H.-M. (2018). Matrine reversed multidrug resistance of breast cancer MCF-7/ADR cells through PI3K/AKT signaling pathway. J. Cell. Biochem. 119, 3885–3891. 10.1002/jcb.26502 29130495

[B263] Zhou Q. Wang S. Zhang H. Lu Y. Wang X. Motoo Y. (2009). The combination of baicalin and baicalein enhances apoptosis via the ERK/p38 MAPK pathway in human breast cancer cells. Acta Pharmacol. Sin. 30, 1648–1658. 10.1038/aps.2009.166 19960010 PMC4007493

[B264] Zhou W. Cao W. Wang M. Yang K. Zhang X. Liu Y. (2023). Validation of quercetin in the treatment of colon cancer with diabetes via network pharmacology, molecular dynamics simulations, and *in vitro* experiments. Mol. Divers. 10.1007/s11030-023-10725-4 37747647

[B265] Zhu M.-L. Zhang P.-M. Jiang M. Yu S.-W. Wang L. (2020). Myricetin induces apoptosis and autophagy by inhibiting PI3K/Akt/mTOR signalling in human colon cancer cells. BMC complementary Med. Ther. 20, 209. 10.1186/s12906-020-02965-w PMC7336643 32631392

[B266] Zubor P. Dankova Z. Kolkova Z. Holubekova V. Brany D. Mersakova S. (2020). Rho GTPases in gynecologic cancers: in-depth analysis toward the paradigm change from reactive to predictive, preventive, and personalized medical approach benefiting the patient and healthcare. Cancers 12, 1292. 10.3390/cancers12051292 32443784 PMC7281750

