# Supplementary material for: Prospects of compounds of herbal plants as anticancer agents: a comprehensive review from molecular pathways
Source: Front Pharmacol. 2024 Jul 22;15:1387866. doi: 10.3389/fphar.2024.1387866 (PMC11298448; doi:10.3389/fphar.2024.1387866)
Supplement: Supplementary file 1 [file Table1.docx]

**Supplementary Table 1.** Phytochemical compounds of flavonoids, alkaloids, terpenoids, steroids, saponins, phenol and tannins.

| Classification | Compounds | Molecular Formula | Chemical structures | Type of cancer | References |
| --- | --- | --- | --- | --- | --- |
| Flavonoids | Apigenin | C_15_H_10_O_5_ | 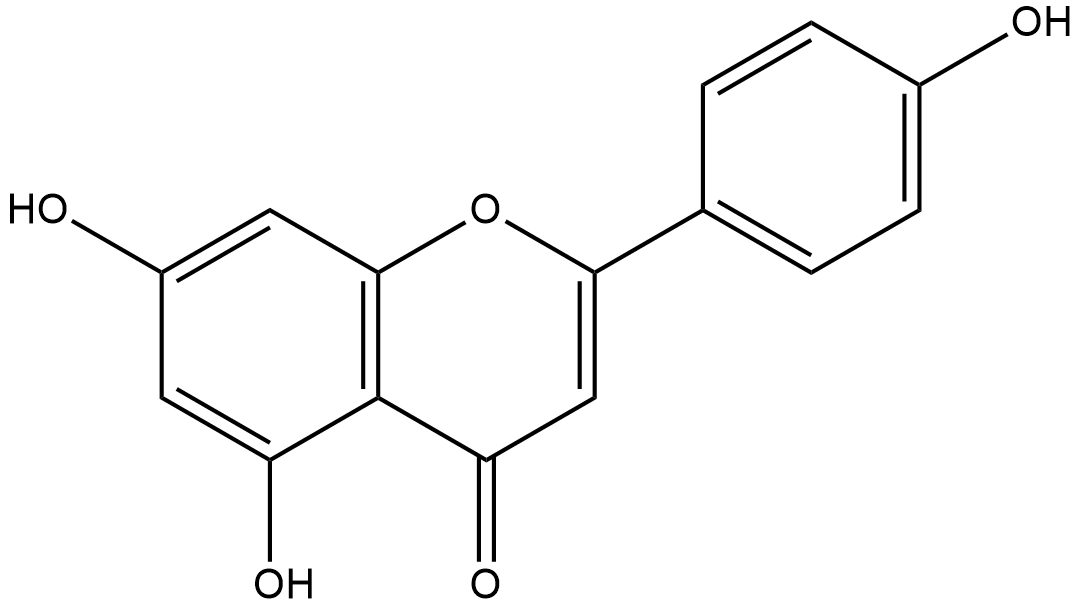 | Lung, Prostate, osteosarcoma | (Nozhat et al., 2021) |
|  | Catechin | C_15_H_14_O_6_ | 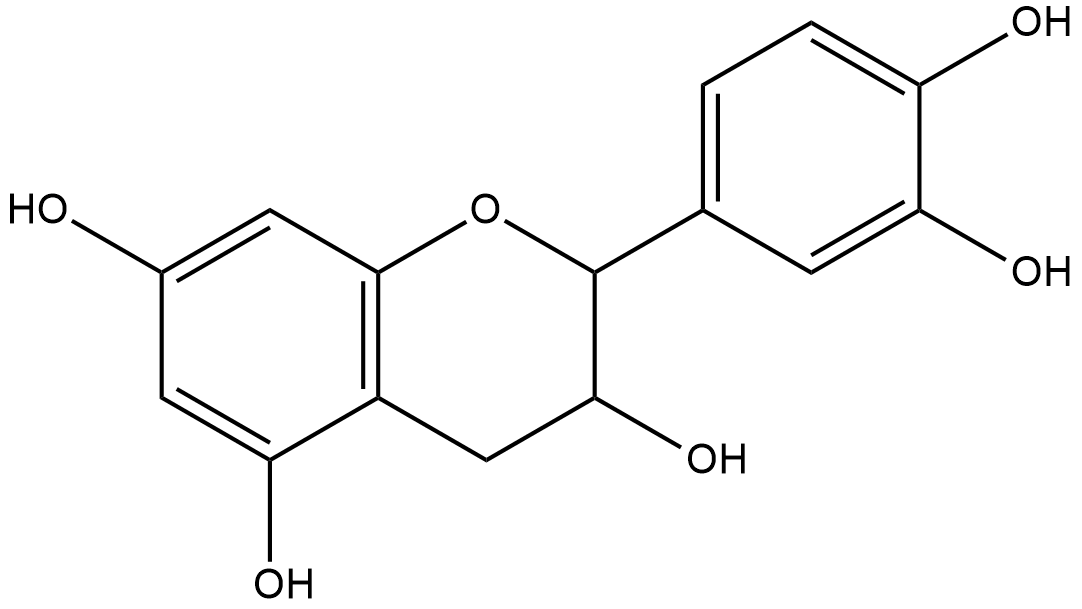 | Lung, Ovarian, Breast, Prostate | (Musial et al., 2020) |
|  | Cyanidin | C_15_H_11_O_6_^+^ | 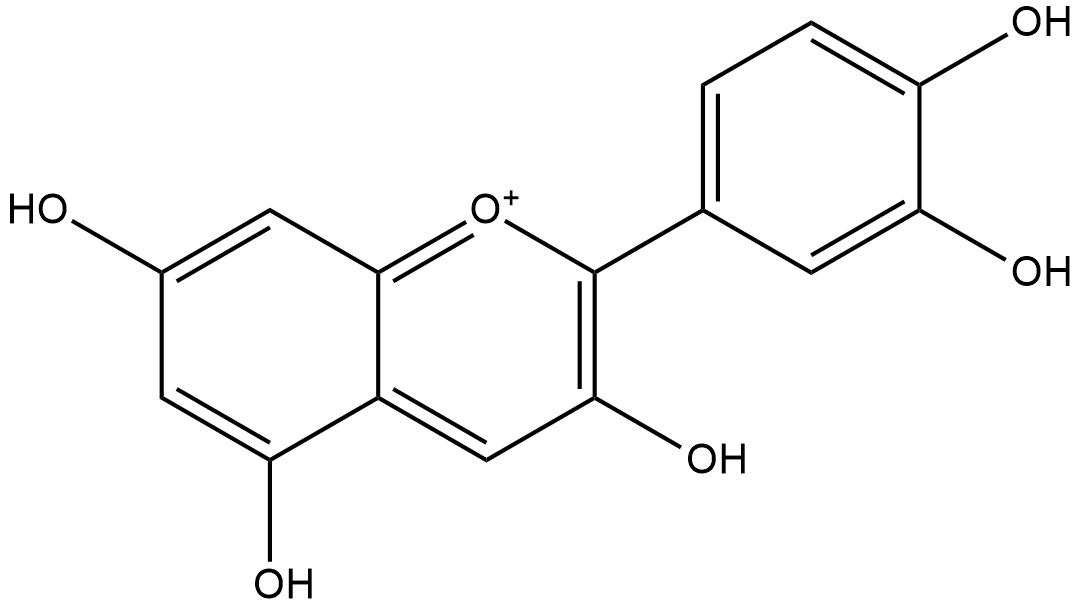 | Colorectal | (Lee et al., 2020a) |
|  | Delphinidin | C_15_H_11_ClO_7_ | 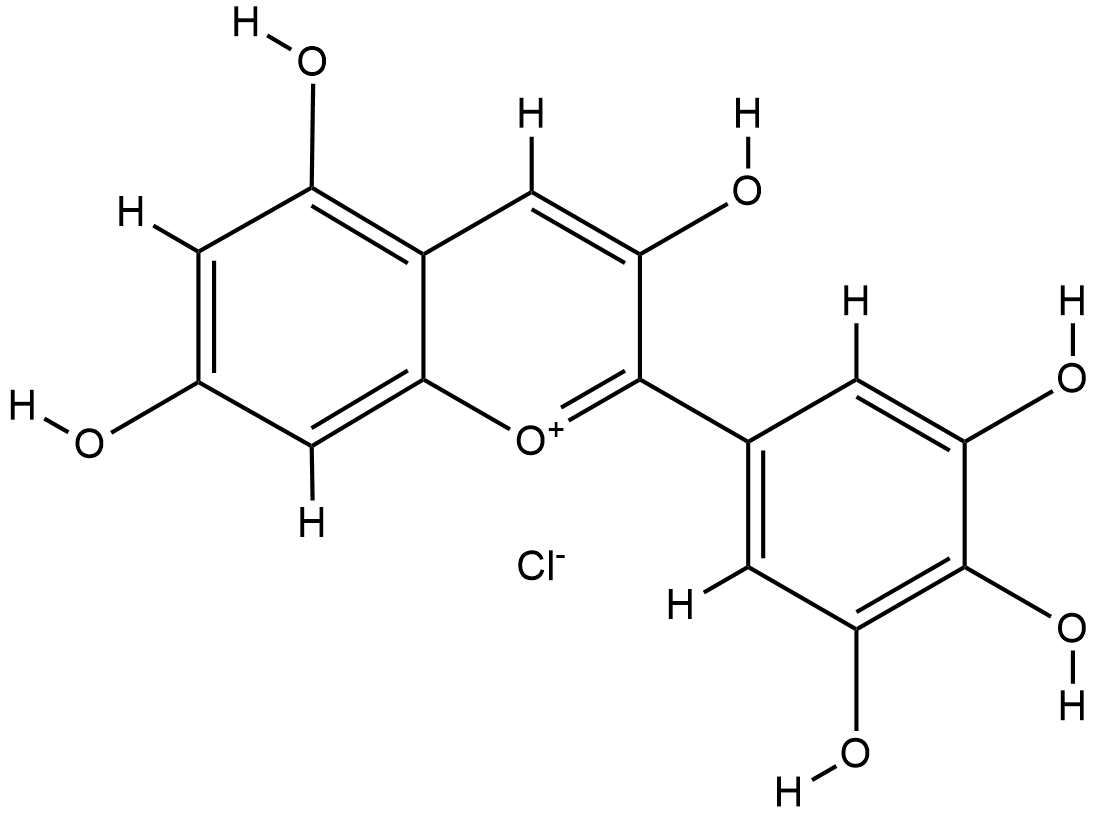 | Lung | (Kang et al., 2020) |
|  | EGCG | C_22_H_18_O_11_ | 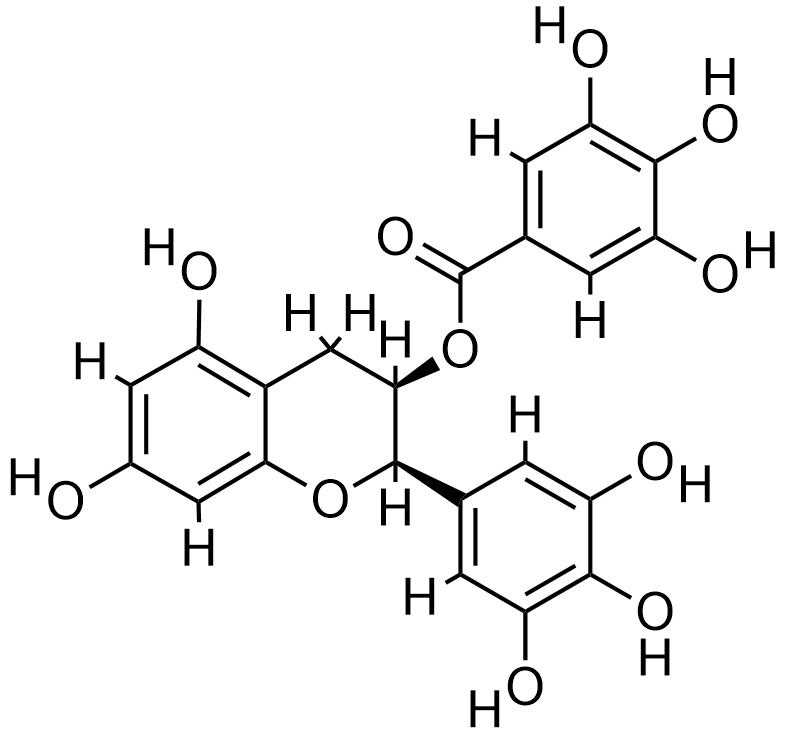 | Leukemia, Glioblastoma | (Yang et al., 2020) |
|  | Galangin | C_15_H_10_O_5_ | 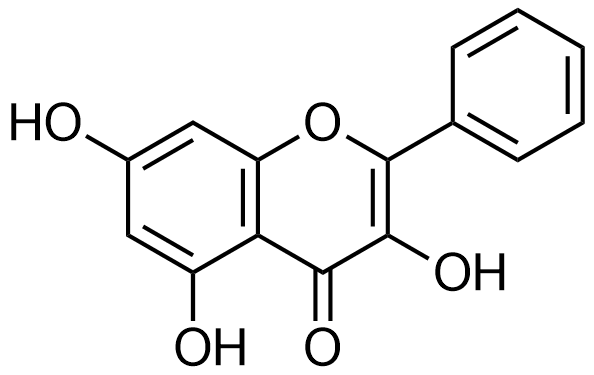 | Kidney, Gastric | (Liang et al., 2021) |
|  | Genistein | C_15_H_10_O_5_ | 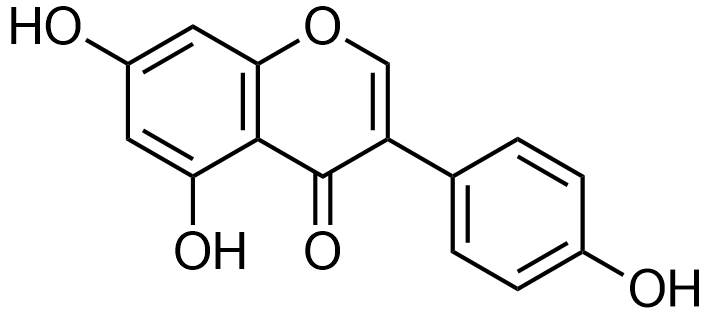 | Breast | (Bhat et al., 2021) |
|  | Hesperetin | C_16_H_14_O_6_ | 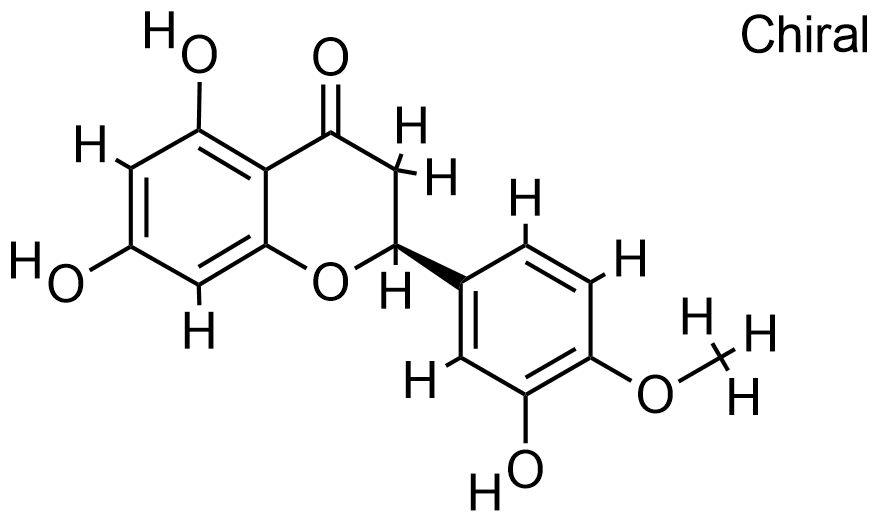 | Lung, Ovarian, Breast, Prostate | (Sohel et al., 2022) |
|  | Kaempferol | C_15_H_10_O_6_ | 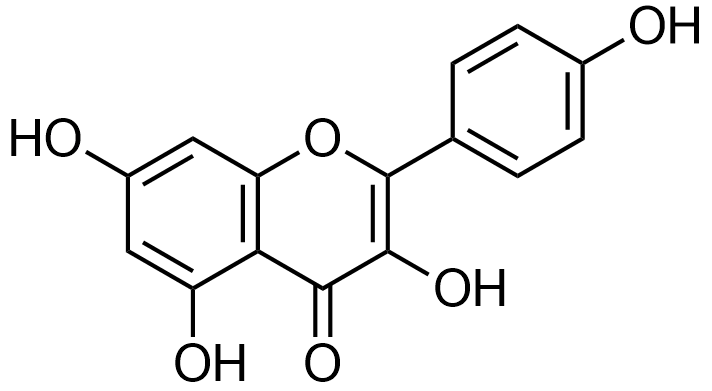 | Colorectal | (Wu et al., 2022a) |
|  | Luteolin | C_15_H_10_O_6_ | 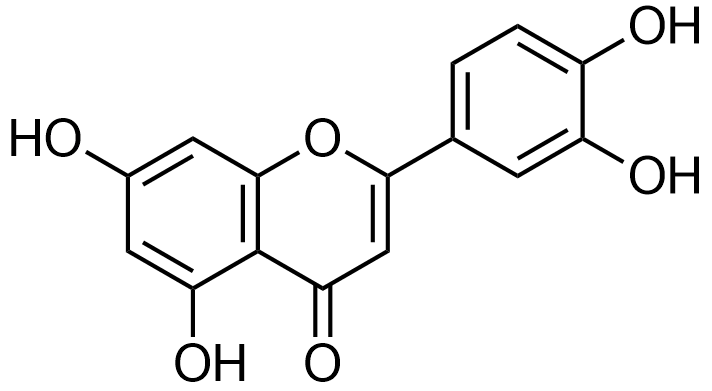 | Colorectal, breast, Glioblastoma | (Hussain et al., 2021) |
|  | Myricetin | C_15_H_10_O_8_ | 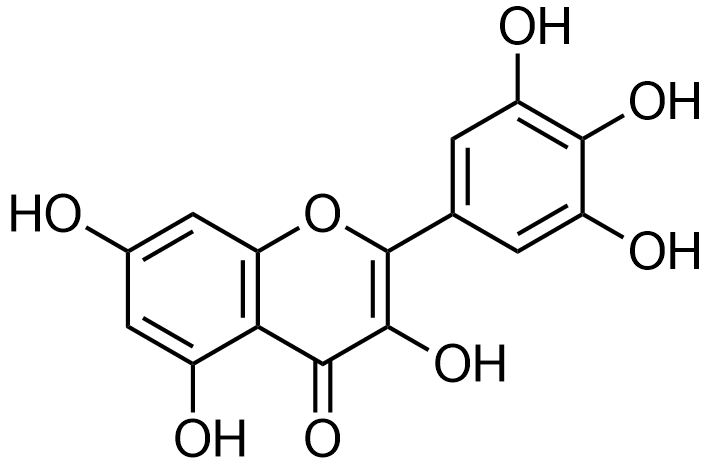 | Lung, Ovarian, Breast, Prostate | (Rahmani et al., 2023) |
|  | Naringenin | C_15_H_12_O_5_ | 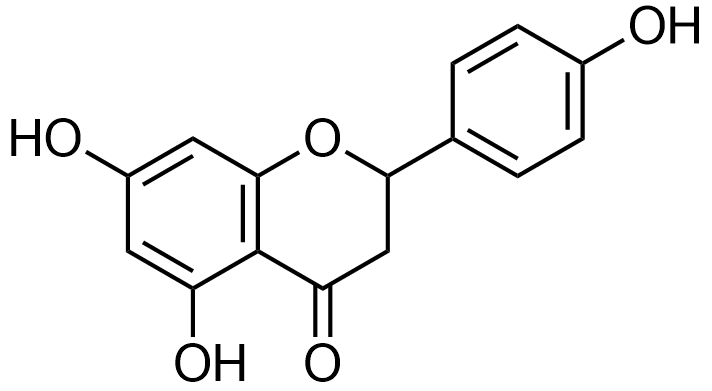 | Prostate, Breast, Cervix | (Madureira et al., 2023) |
|  | Tangeritin | C_20_H_20_O_7_ | 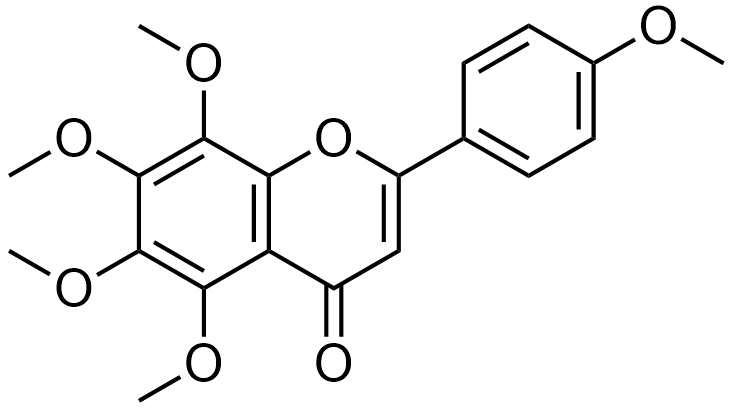 | Oral cancer cells | (Gv et al., 2023) |
|  | Quercetin | C_15_H_10_O_7_ | 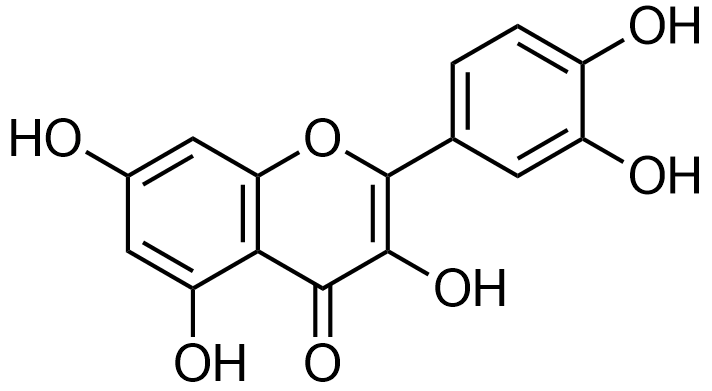 | Lung, Ovarian, Breast, Prostate | (Asgharian et al., 2022) |
| Alkaloids | Arecoline | C_8_H_13_NO_2_ | 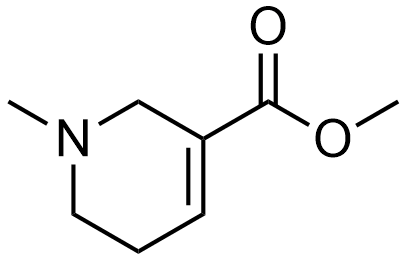 | Oral cancer cells | (Ko et al., 2023) |
|  | Cephaeline | C_28_H_38_N_2_O_4_ | 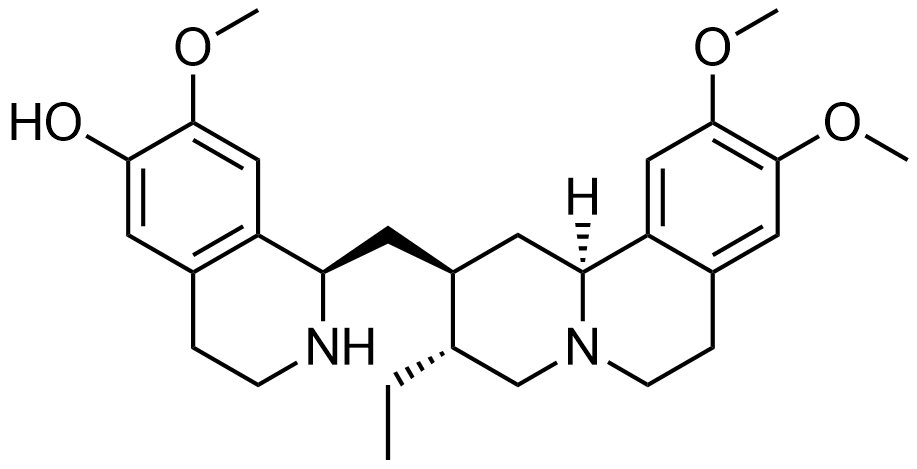 | carcinoma cancer | (Silva et al., 2022) |
|  | Cinchonine | C_19_H_22_N_2_O | 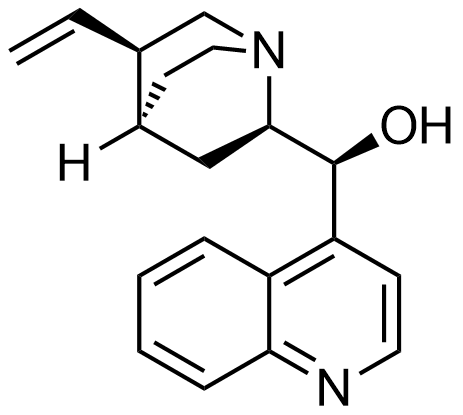 | Pancreatic | (Liang et al., 2023) |
|  | Hygrine | C_8_H_15_NO | 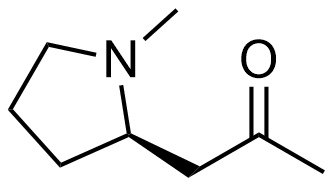 | Breast, Prostate | (Olofinsan et al., 2023) |
|  | Morphine | C_17_H_19_NO_3_ | 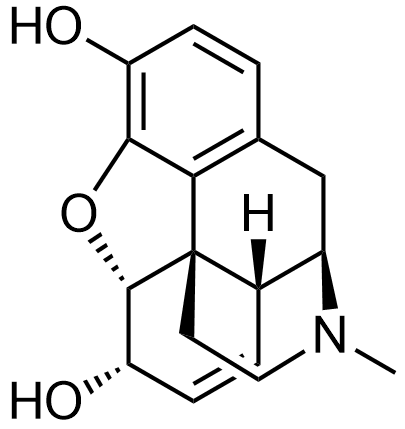 | Lung, Ovarian | (Ho et al., 2020) |
|  | Nicotine | C_10_H_14_N_2_ | 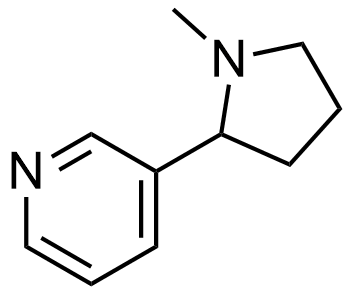 | Lung, Breast | (Tyagi et al., 2021) |
|  | Reserpine | C_33_H_40_N_2_O_9_ | 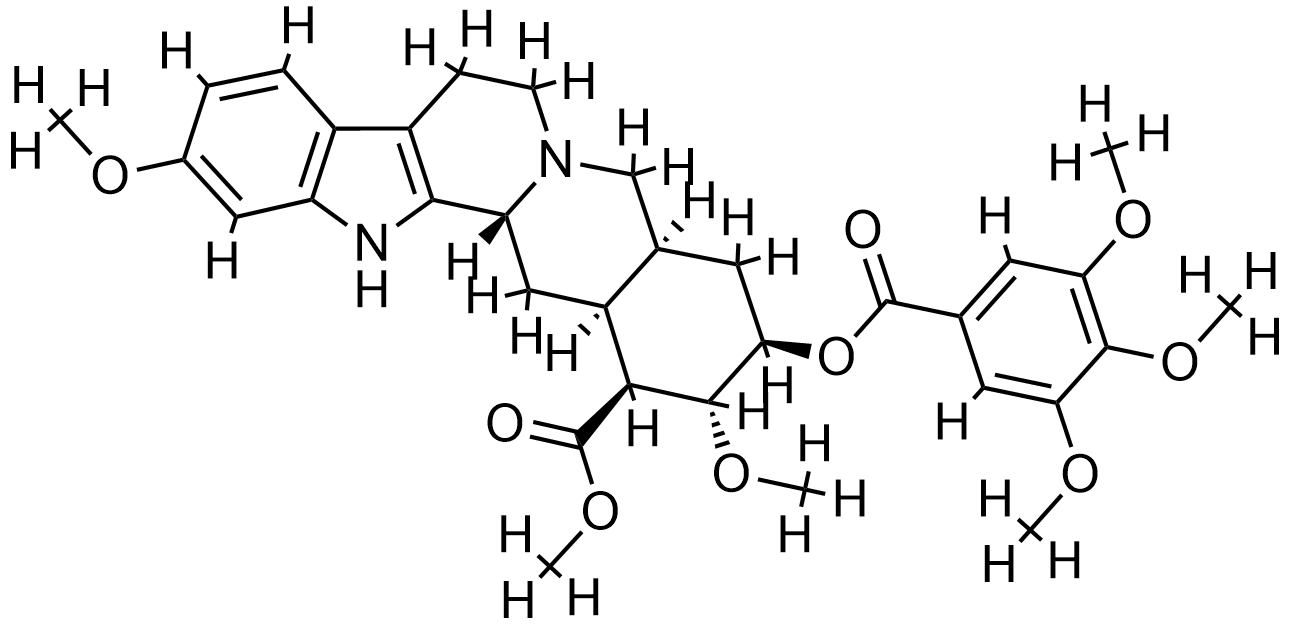 | Lung | (Senthamizh et al., 2020) |
|  | Serotonin | C_10_H_12_N_2_O | 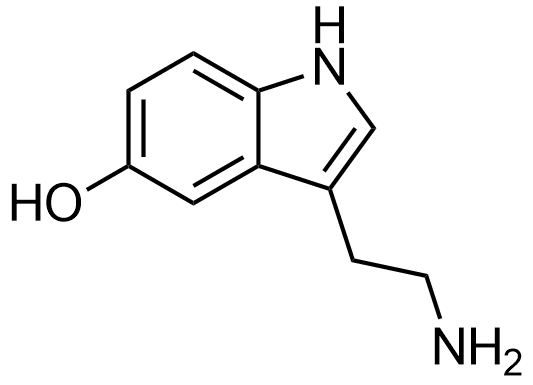 | Cervix, Lung, Ovarian, Breast, Prostate | (Balakrishna et al., 2021) |
|  | Trigonelline | C_7_H_7_NO_2_ | 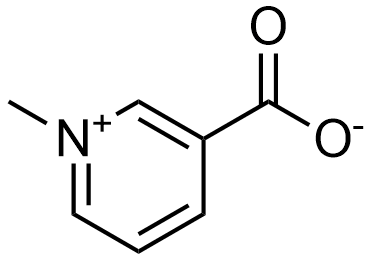 | Colon | (Pirpour Tazehkand et al., 2020) |
|  | Vincristine | C_46_H_56_N_4_O_10_ | 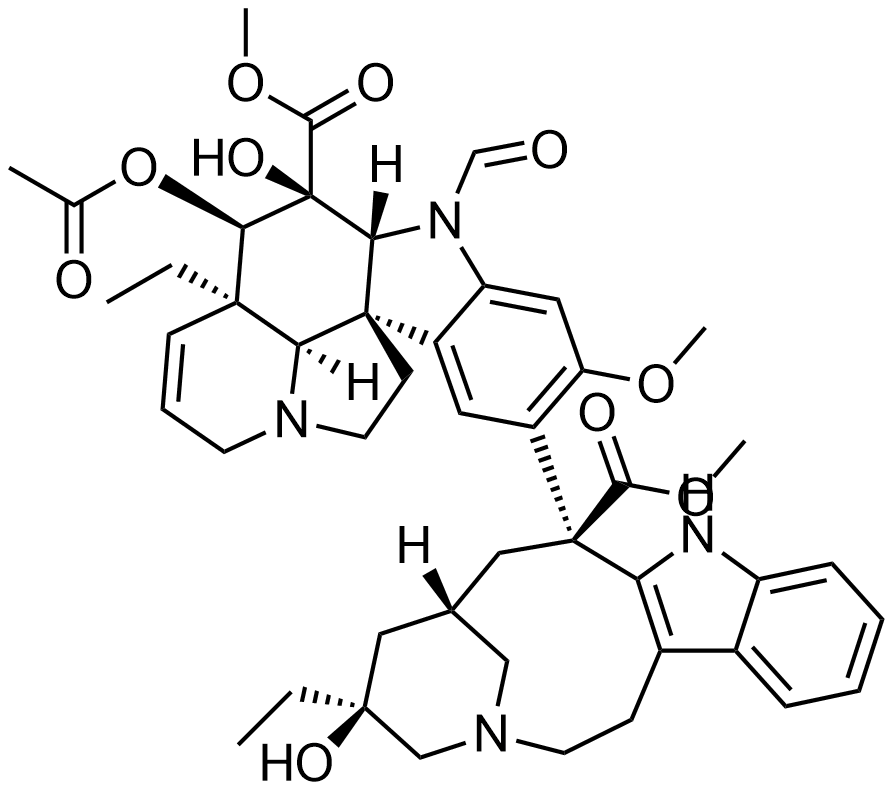 | Cervix, Ovarian, Breast | (Škubník et al., 2021) |
| Terpenoids | Ambrosin | C_15_H_18_O_3_ | 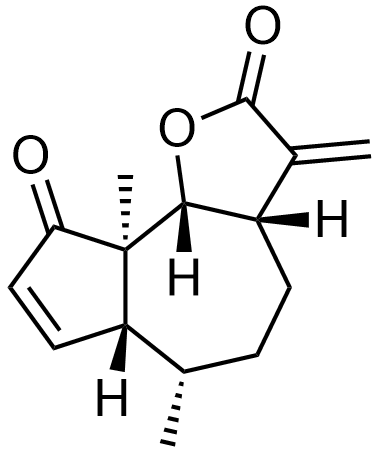 | Breast | (Meng and Shao, 2021) |
|  | Ascaridole | C_10_H_16_O_2_ | 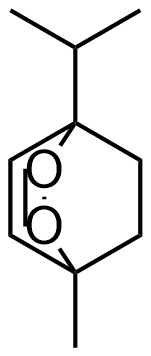 | Cervix, Lung, Ovarian, Breast | (PATEL and Patel, 2021) |
|  | Auraptene | C_19_H_22_O_3_ | 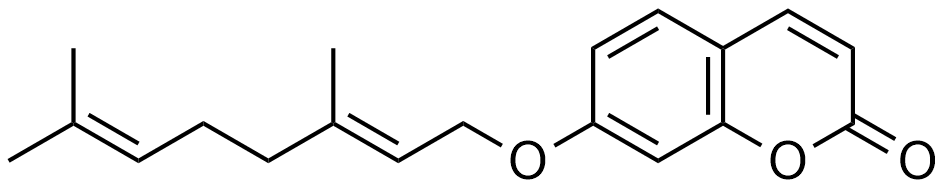 | Prostate, Breast, Cervix | (Tayarani-Najaran et al., 2021) |
|  | Betulinic acid | C_30_H_48_O_3_ | 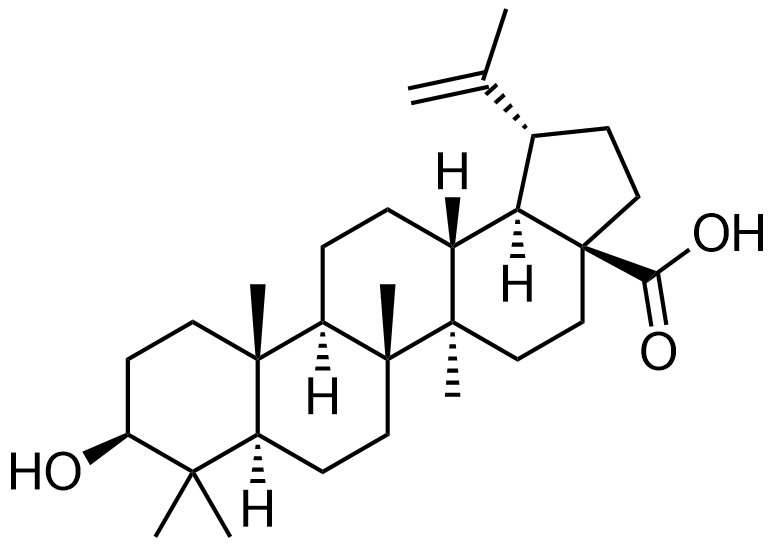 | human leukemia cells | (Park et al., 2021) |
|  | Carvacrol | C_10_H_14_O | 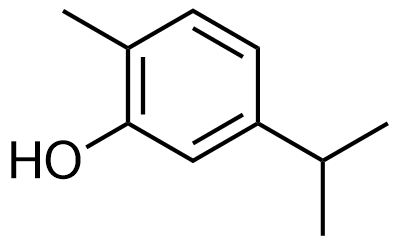 | Breast | (Mari et al., 2021) |
|  | Costunolide | C_15_H_20_O_2_ | 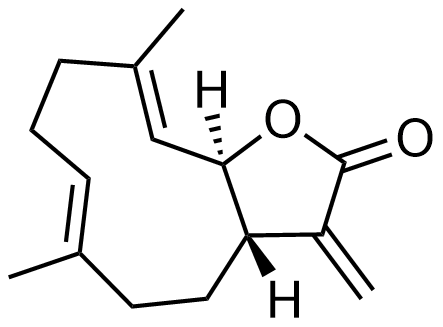 | Lung | (Wei et al., 2020) |
|  | Dehydrocostus lactone | C_15_H_18_O_2_ | 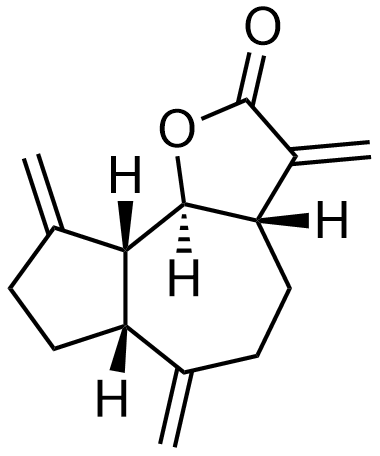 | Laryngeal carcinoma | (Zhang et al., 2020) |
|  | Limonene | C_10_H_16_ | 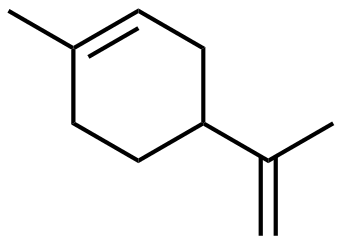 | Breast | (Chebet et al., 2021) |
|  | Menthol | C_10_H_20_O | 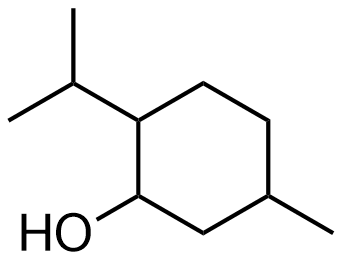 | Cervix, Ovarian, Breast | (Zhao et al., 2023) |
|  | Thymol | C_10_H_14_O | 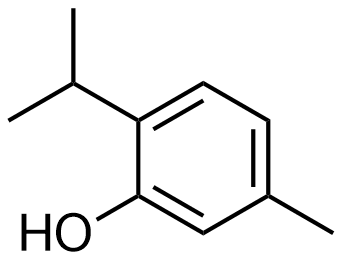 | Colorectal | (Zeng et al., 2020) |
|  | Thymoquinone | C_10_H_12_O_2_ | 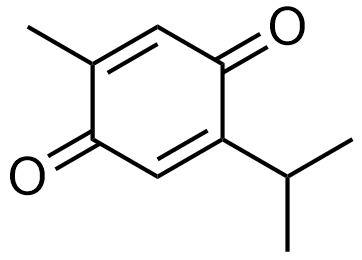 | Prostate, Breast, Cervix, Lung | (Almatroodi et al., 2020) |
|  | Triptolide | C_20_H_24_O_6_ | 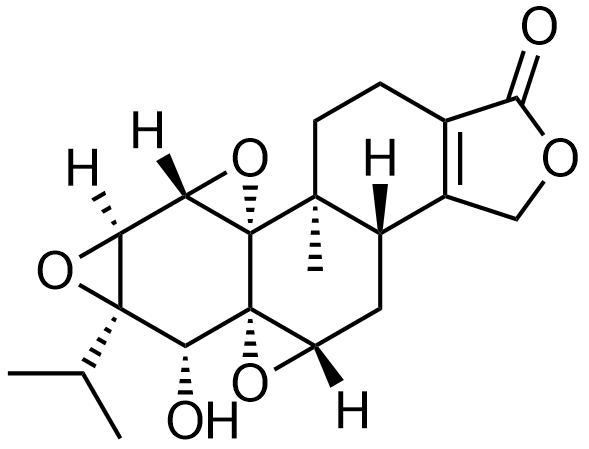 | Pancreatic | (Zhao et al., 2020) |
|  | Ursolic acid | C_30_H_48_O_3_ | 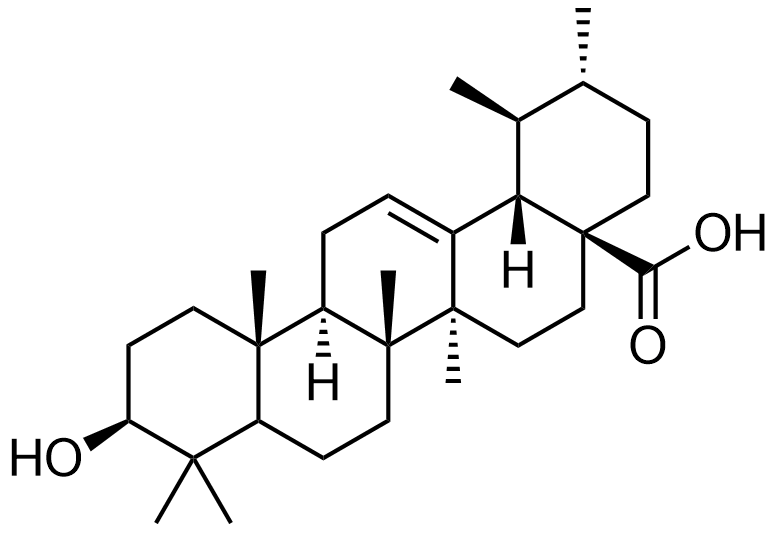 | Prostate, Breast, Lung | (Miatmoko et al., 2021) |
|  | β-Elemene | C_15_H_24_ | 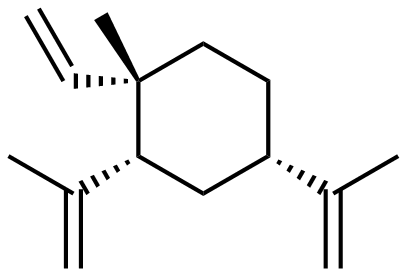 | Bladder | (Gan et al., 2020) |
| Steroids | Aglaia sterols | C_27_H_44_O | 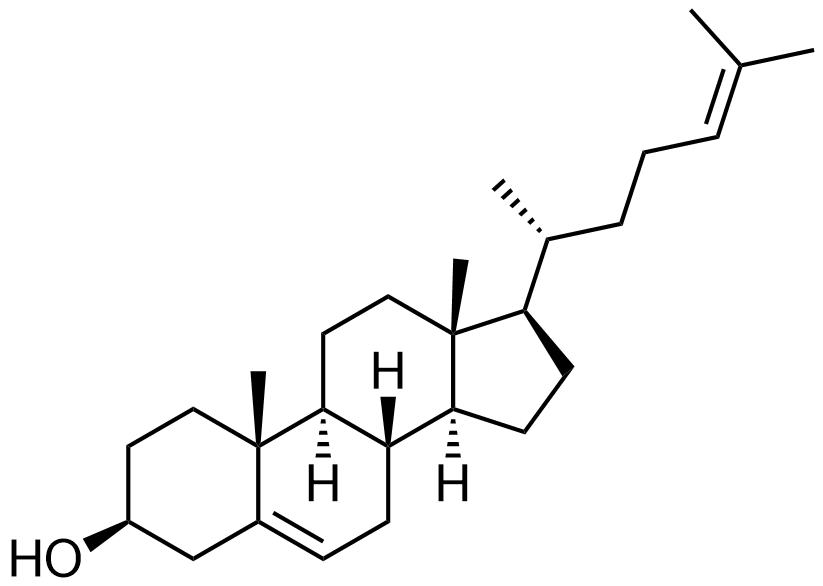 | Cervix | (Kurniasih et al., 2021) |
|  | Arenobufagin | C_24_H_32_O_6_ | 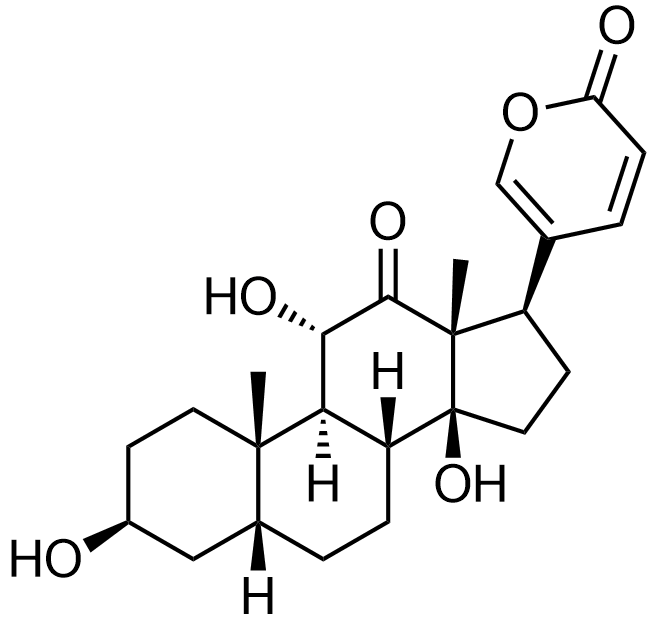 | Breast | (Zhang et al., 2021b) |
|  | Alocasgenol | C_21_H_32_O_5_ | 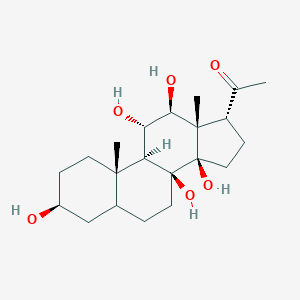 | Prostate, Breast, Lung | (Xiao et al., 2020) |
|  | Cholest-4-ene-3,6-dione | C_27_H_42_O_2_ | 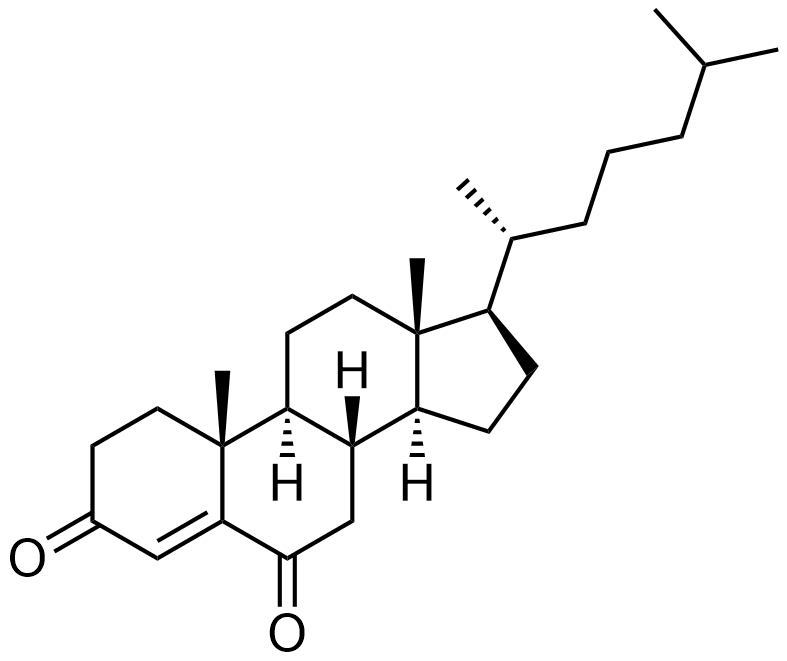 | Breast | (Sali et al., 2020) |
|  | Eburicol | C_31_H_52_O | 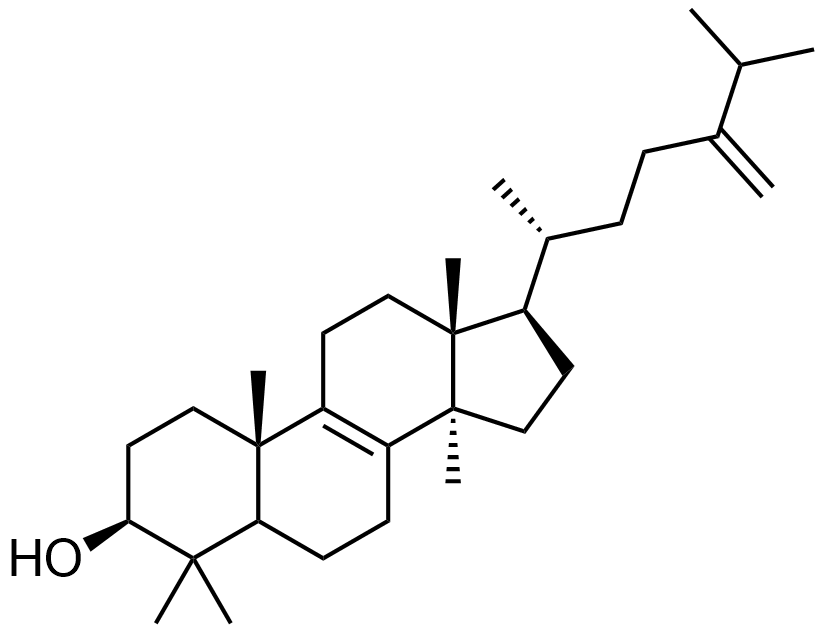 | Hepatocarcinoma | (Ray et al., 2022) |
|  | Entanutilin T | C_38_H_45_NO_12_ | 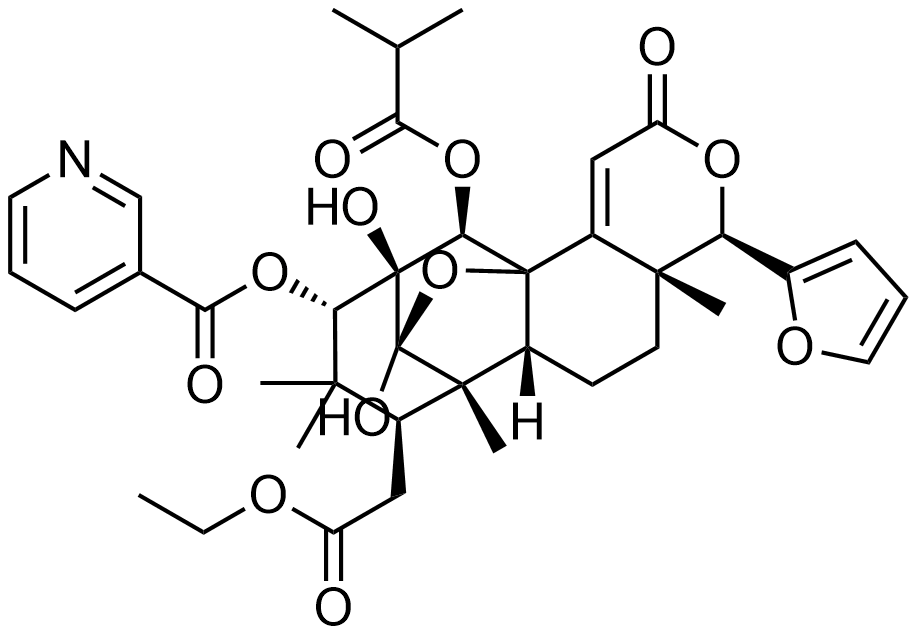 | Sarcoma, pancreatic cancer, prostate cancer, lymphocytic leukemia, myeloid leukemia, | (Dembitsky et al., 2021) |
|  | Ganodermaside E | C_28_H_40_O_2_ | 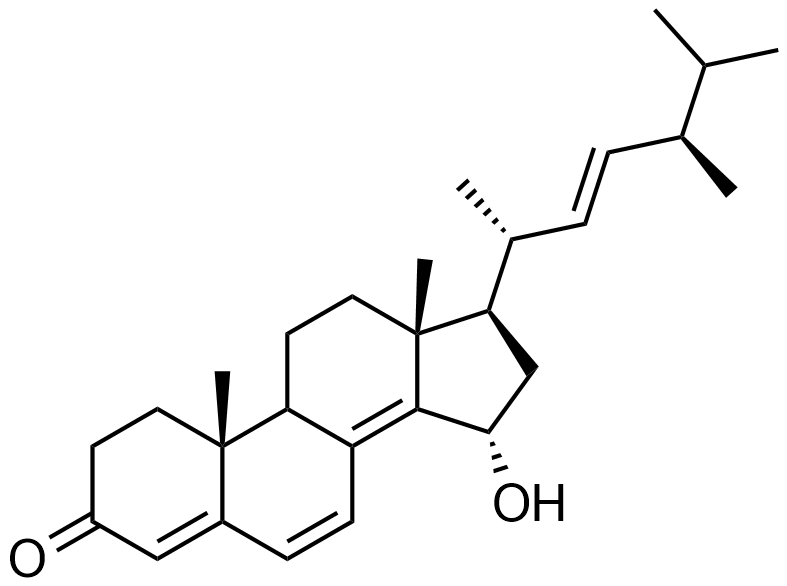 | Breast | (Xiao et al., 2020) |
|  | Klyflaccisteroid J | C_29_H_48_O_3_ | 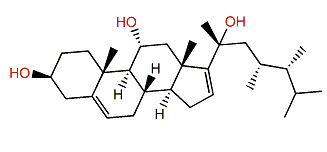 | Breast | (Reddy et al., 2020) |
|  | Verrucorosterone | C_29_H_44_O_5_ | 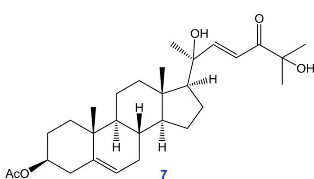 | Breast | (Xiao et al., 2020) |
| Saponins | Diosgenin | C_27_H_42_O_3_ | 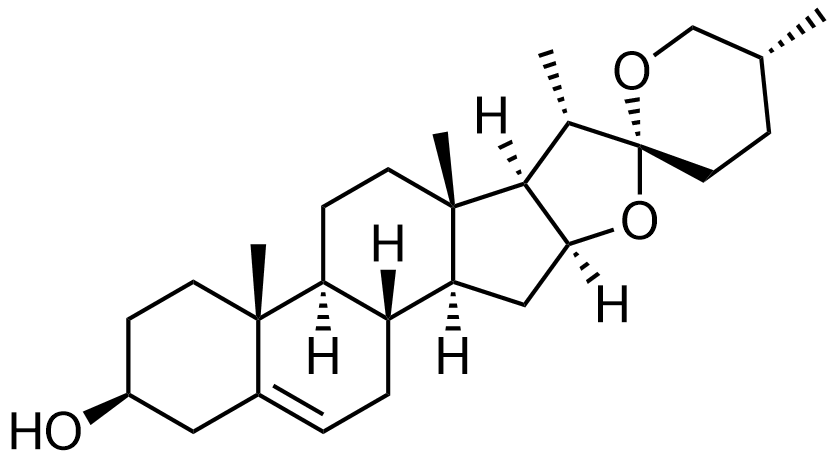 | Breast | (Liu et al., 2005). |
|  | Dioscin | C_45_H_72_O_16_ | 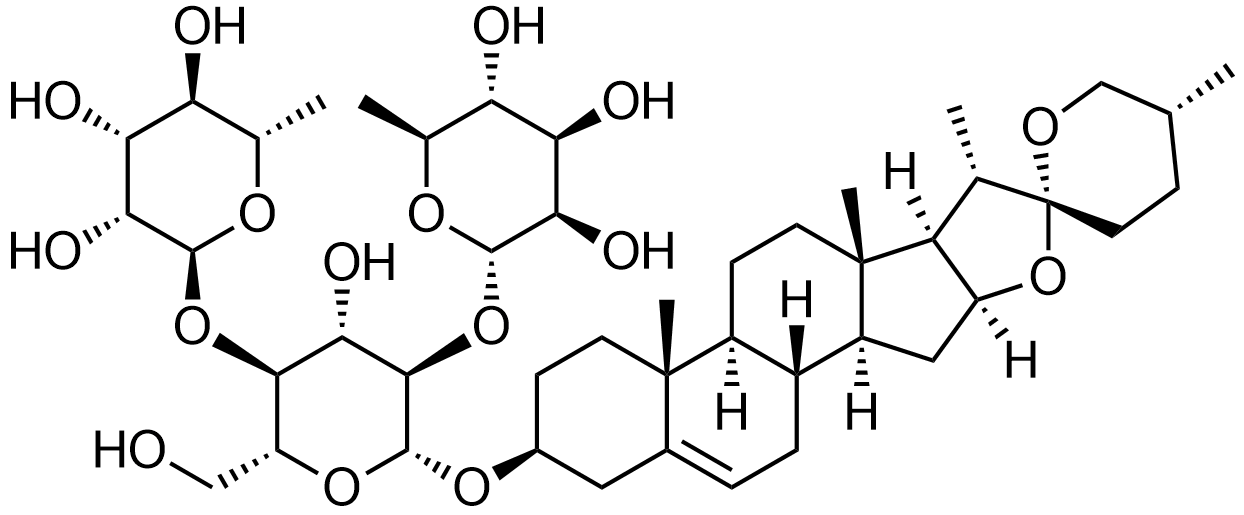 | Lung, Leukimia, Cervix | (Zhao et al., 2016). |
|  | Polyphyllin D | C_44_H_70_O_16_ | 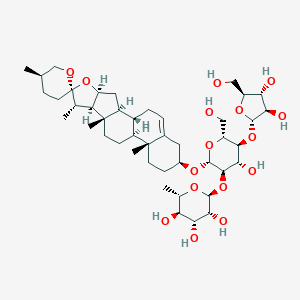 | Ovarian, Breast | (Lee et al., 2005) |
|  | Oleandrin | C_32_H_48_O_9_ | 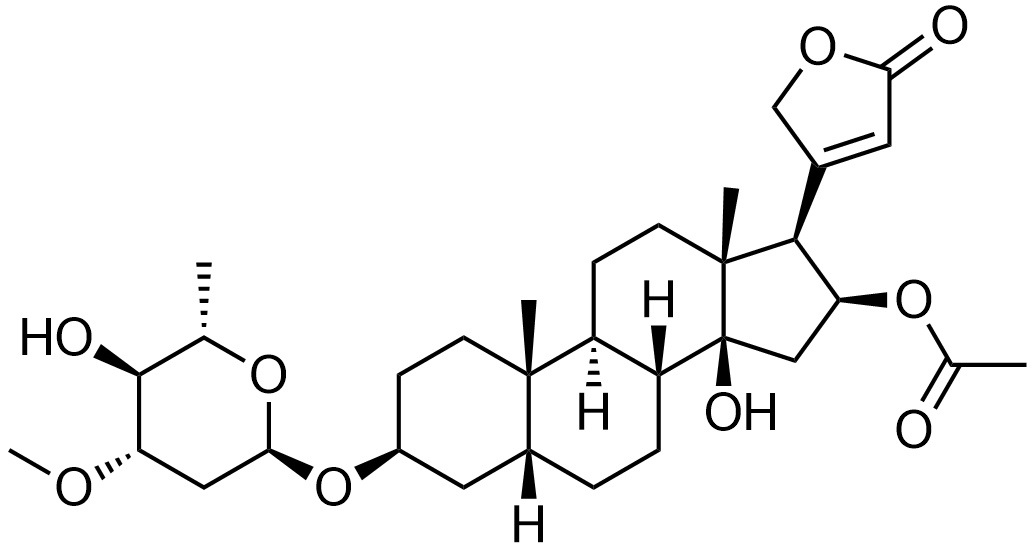 | Pancreatic, Prostate, Breast | (Kanwal et al., 2020) |
|  | Ginsenoside Rh2 | C_36_H_62_O_8_ | 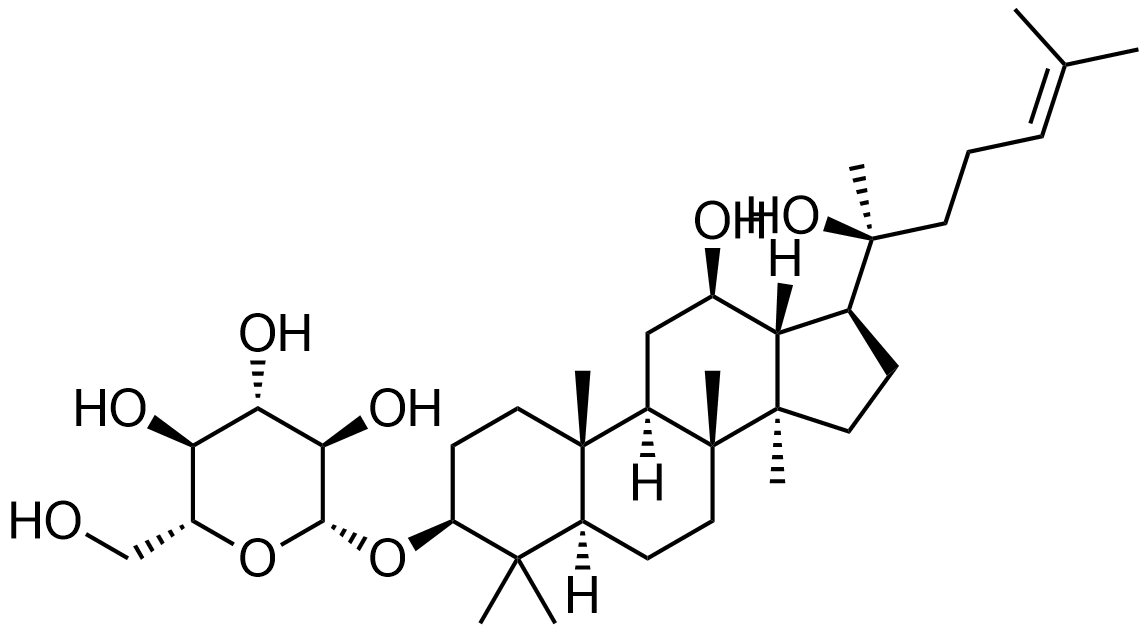 | Colon, Lung, Gastric | (Yuan et al., 2017) |
|  | Saikosaponin A | C_42_H_68_O_13_ | 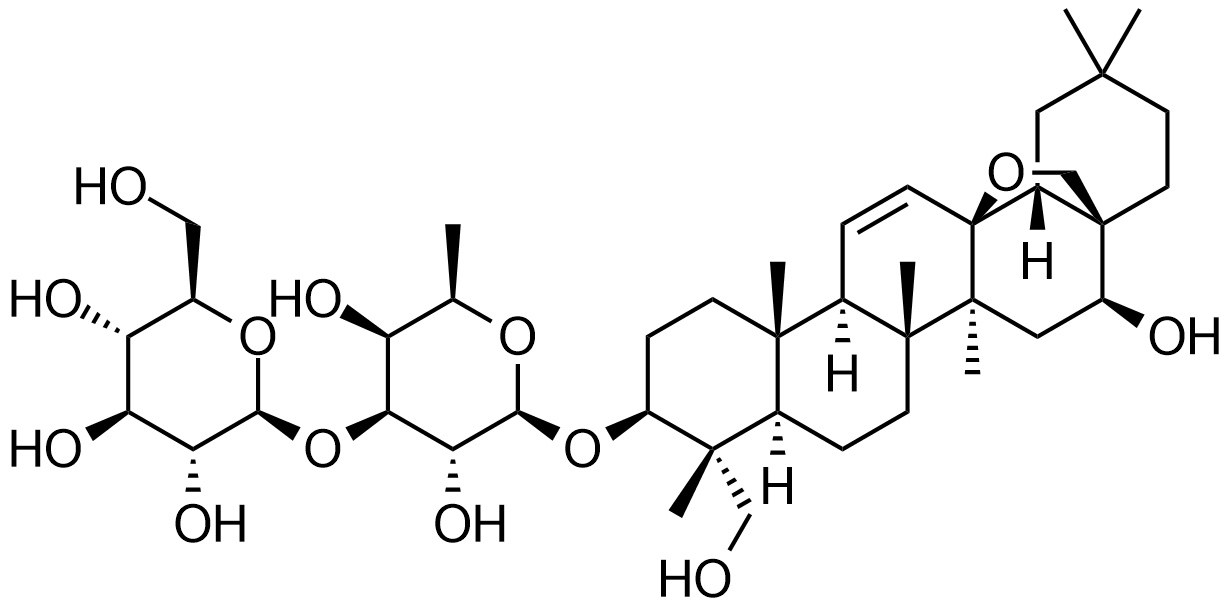 | Colon, Breast | (Yuan et al., 2017) |
|  | Saikosaponin D | C_42_H_68_O_13_ | 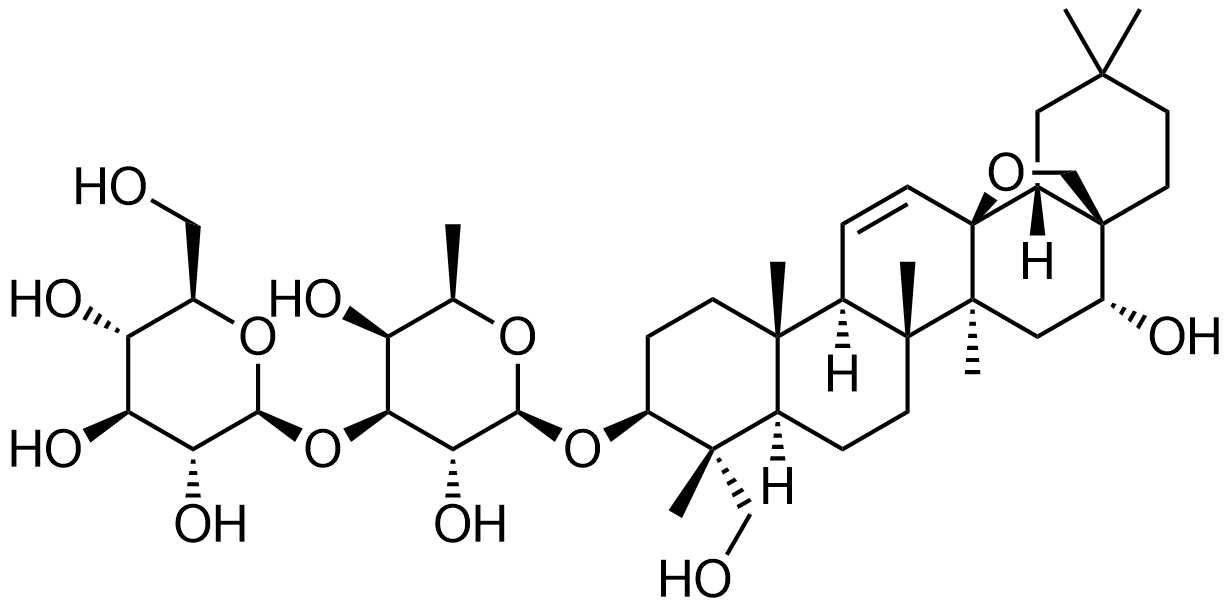 | Lung, Hepatoceluler | (Wang et al., 2014a) |
|  | Timosaponin AIII (TAIII) | C_39_H_64_O_13_ | 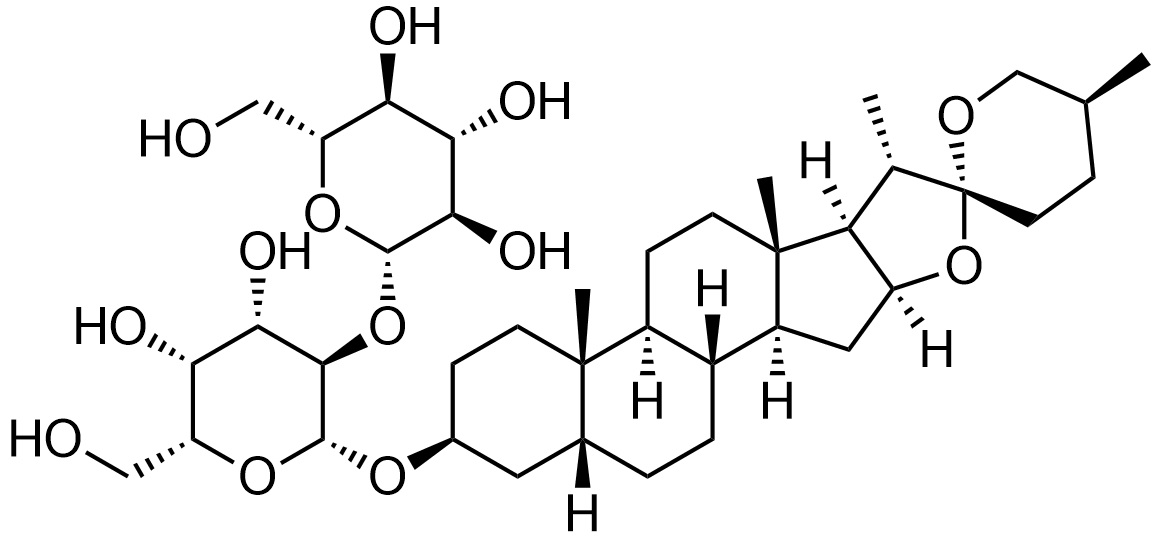 | Breast, Prostate | (Tsai et al., 2013) |
| Phenolic | Caffeic acid | C_9_H_8_O_4_ | 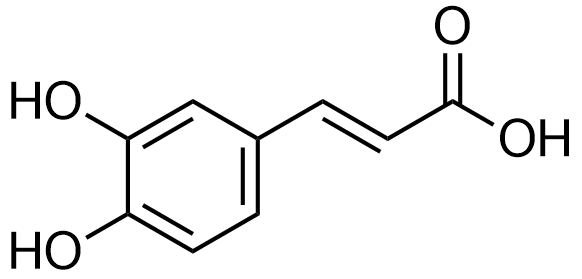 | Renal carcinoma | (Jung et al., 2007) |
|  | Curcumin | C_21_H_20_O_6_ | 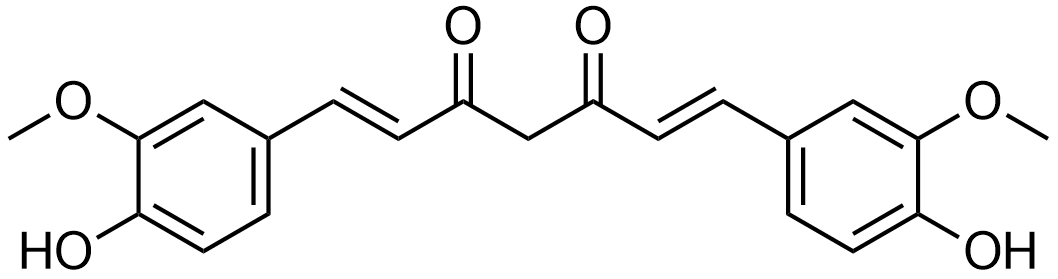 | Breast | (Kim et al., 2001)s |
|  | Gallic acid | C_7_H_6_O_5_ | 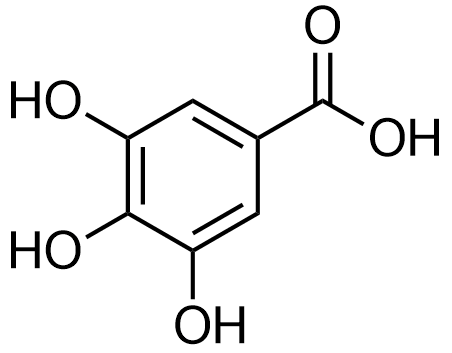 | Lung,  Colorectal Cancer | (Sanchez-Martin et al., 2022) |
|  | Rosmarinic acid | C_18_H_16_O_8_ | 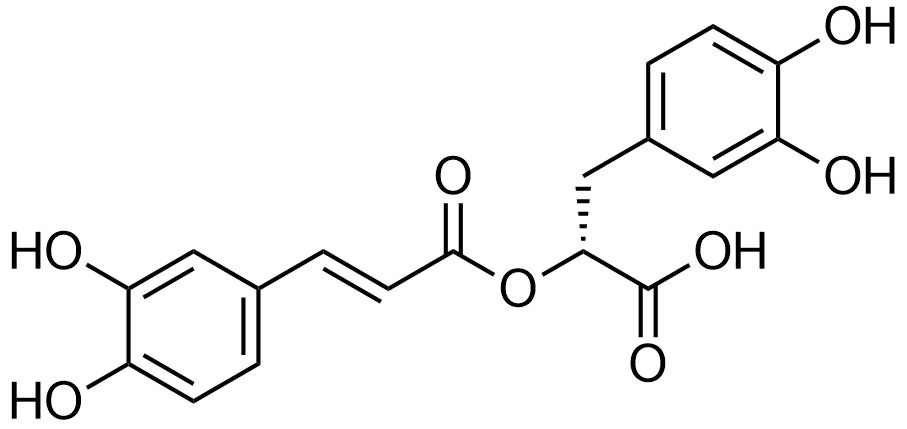 | Breast | (Mahmoud et al., 2021) |
|  | Sinapic acid | C_11_H_12_O_5_ | 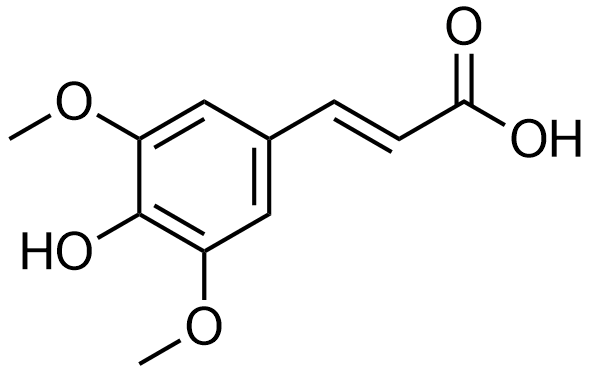 | Colon | (Balaji et al., 2014) |
|  | Quercetine | C_15_H_10_O_7_ | 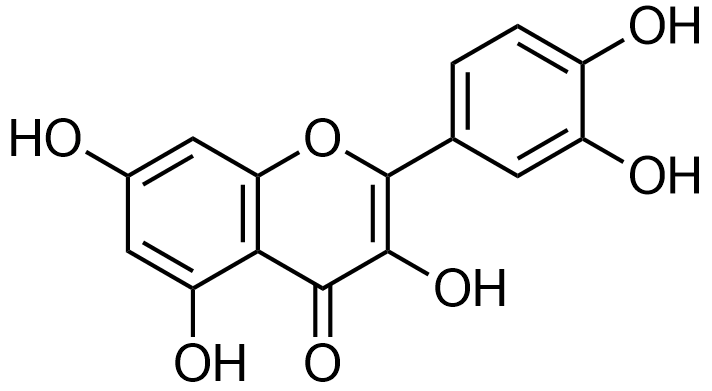 | Prostate | (Noori-Daloii et al., 2011) |
| Tannins | Brivudin | C_11_H_13_BrN_2_O_5_ | 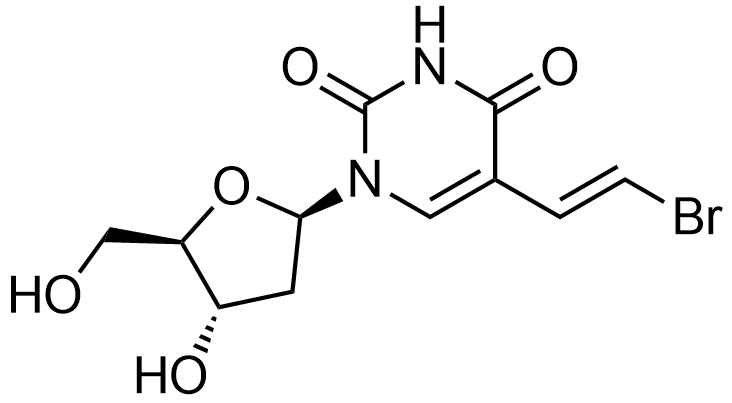 | Oral Mucosa | (Yamada et al., 2023) |
|  | Acyclovir | C_8_H_11_N_5_O_3_ | 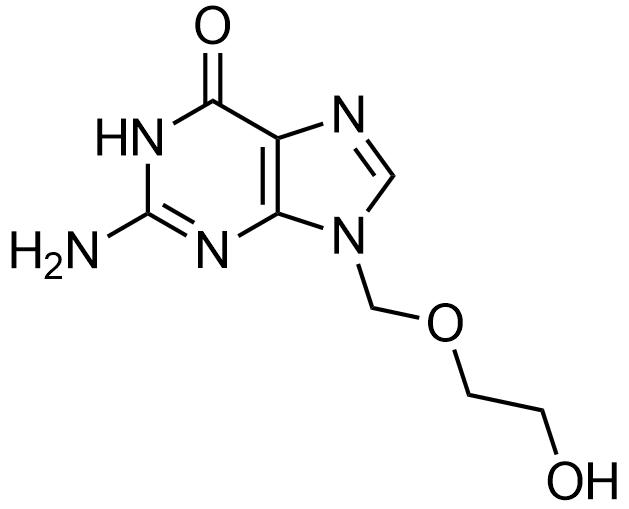 | Breast | (Xie et al., 2020) |
|  | Caffeic-tannins | C_16_H_18_O_9_ | 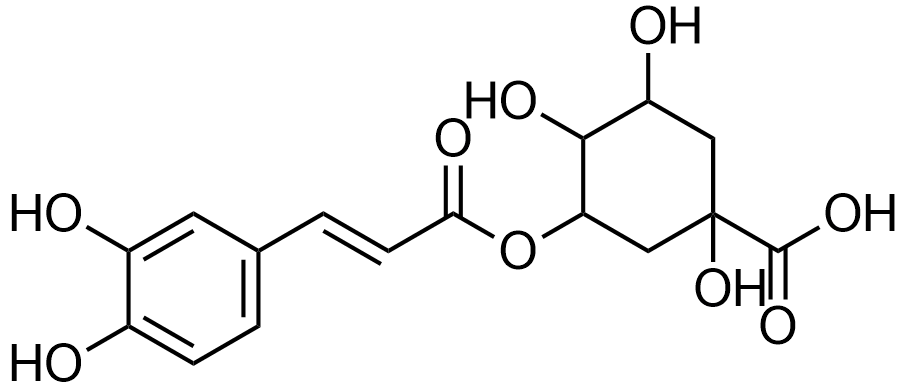 | Breast | (Pizzi, 2021) |
|  | Ganciclovir | C_9_H_13_N_5_O_4_ | 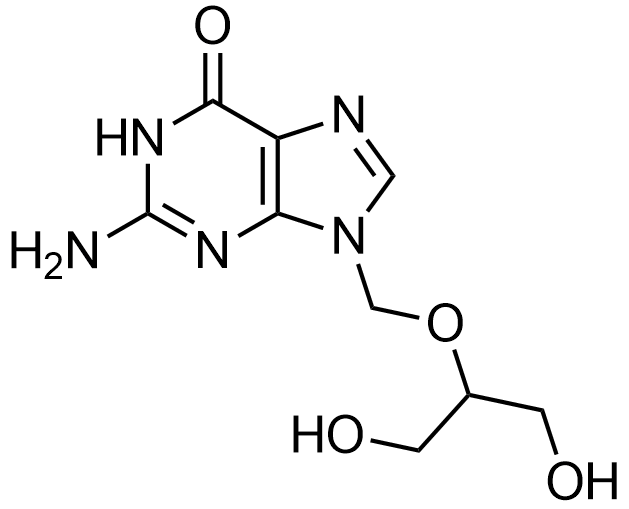 | Prostate | (Djavan and Nasu, 2001) |
|  | Ribavirin | C_8_H_12_N_4_O_5_ | 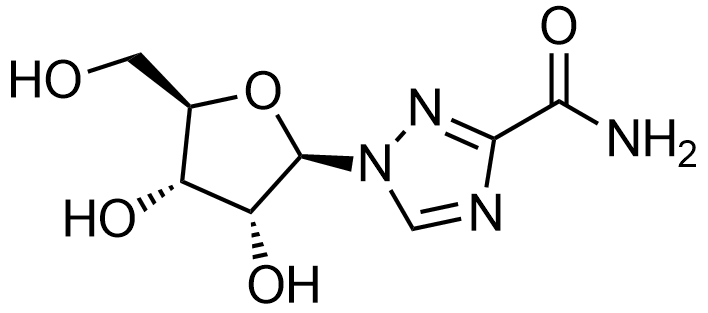 | Pancreatic, prostate, lymphocytic leukemia | (Kast, 2003) |
|  | Tara tannins | C_42_H_32_O_26_ | 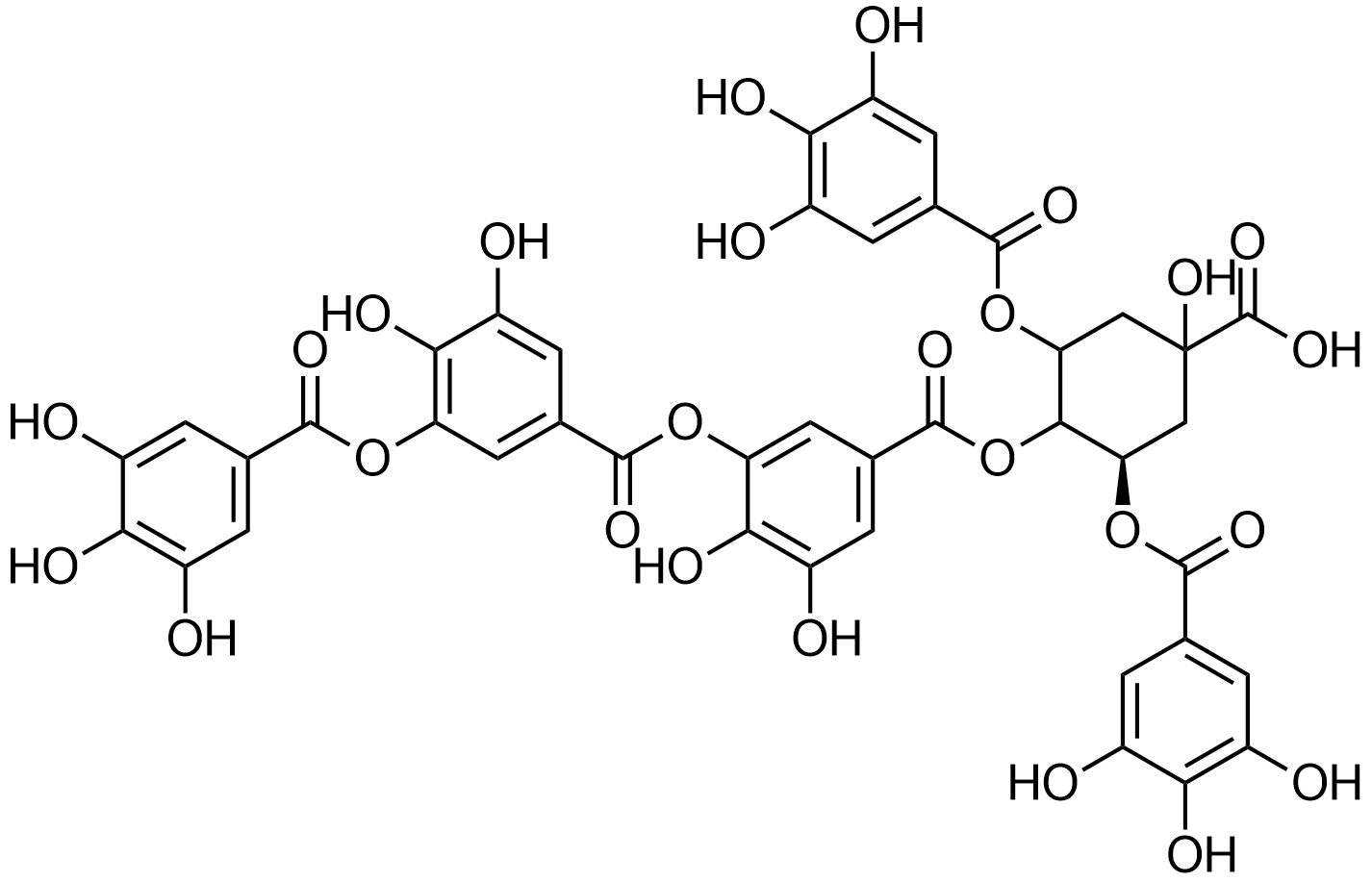 | Melanoma | (Villareal et al., 2020) |
|  | Vescalin | C_27_H_20_O_18_ | 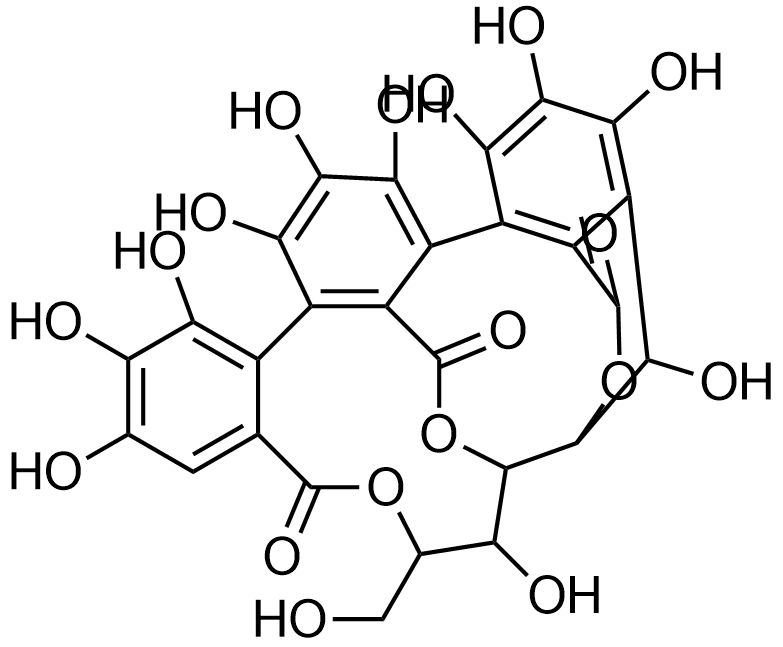 | Neuroblastoma Cells | (Quideau et al., 2005) |
